# Secondary metabolite biosynthetic diversity in the fungal family *Hypoxylaceae* and *Xylaria hypoxylon*

**DOI:** 10.1016/j.simyco.2021.100118

**Published:** 2021-08-26

**Authors:** E. Kuhnert, J.C. Navarro-Muñoz, K. Becker, M. Stadler, J. Collemare, R.J. Cox

**Affiliations:** 1 Centre of Biomolecular Drug Research (BMWZ), Institute for Organic Chemistry, Leibniz University Hannover, Schneiderberg 38, 30167, Hannover, Germany; 2 Westerdijk Fungal Biodiversity Institute, Uppsalalaan 8, 3584 CT, Utrecht, The Netherlands; 3 Department Microbial Drugs, Helmholtz Centre for Infection Research (HZI), German Centre for Infection Research (DZIF), partner site Hannover-Braunschweig, Inhoffenstrasse 7, 38124, Braunschweig, Germany

**Keywords:** Azaphilones, Binaphthalenes, Biosynthesis, Comparative genomics, Metabolomics, Natural products, *Xylariales*

## Abstract

To date little is known about the genetic background that drives the production and diversification of secondary metabolites in the *Hypoxylaceae*. With the recent availability of high-quality genome sequences for 13 representative species and one relative (*Xylaria hypoxylon*) we attempted to survey the diversity of biosynthetic pathways in these organisms to investigate their true potential as secondary metabolite producers. Manual search strategies based on the accumulated knowledge on biosynthesis in fungi enabled us to identify 783 biosynthetic pathways across 14 studied species, the majority of which were arranged in biosynthetic gene clusters (BGC). The similarity of BGCs was analysed with the BiG-SCAPE engine which organised the BGCs into 375 gene cluster families (GCF). Only ten GCFs were conserved across all of these fungi indicating that speciation is accompanied by changes in secondary metabolism. From the known compounds produced by the family members some can be directly correlated with identified BGCs which is highlighted herein by the azaphilone, dihydroxynaphthalene, tropolone, cytochalasan, terrequinone, terphenyl and brasilane pathways giving insights into the evolution and diversification of those compound classes. *Vice versa*, products of various BGCs can be predicted through homology analysis with known pathways from other fungi as shown for the identified ergot alkaloid, trigazaphilone, curvupallide, viridicatumtoxin and swainsonine BGCs. However, the majority of BGCs had no obvious links to known products from the *Hypoxylaceae* or other well-studied biosynthetic pathways from fungi. These findings highlight that the number of known compounds strongly underrepresents the biosynthetic potential in these fungi and that a tremendous number of unidentified secondary metabolites is still hidden. Moreover, with increasing numbers of genomes for further *Hypoxylaceae* species becoming available, the likelihood of revealing new biosynthetic pathways that encode new, potentially useful compounds will significantly improve. Reaching a better understanding of the biology of these producers, and further development of genetic methods for their manipulation, will be crucial to access their treasures.

## Introduction

**Fig. 1:**
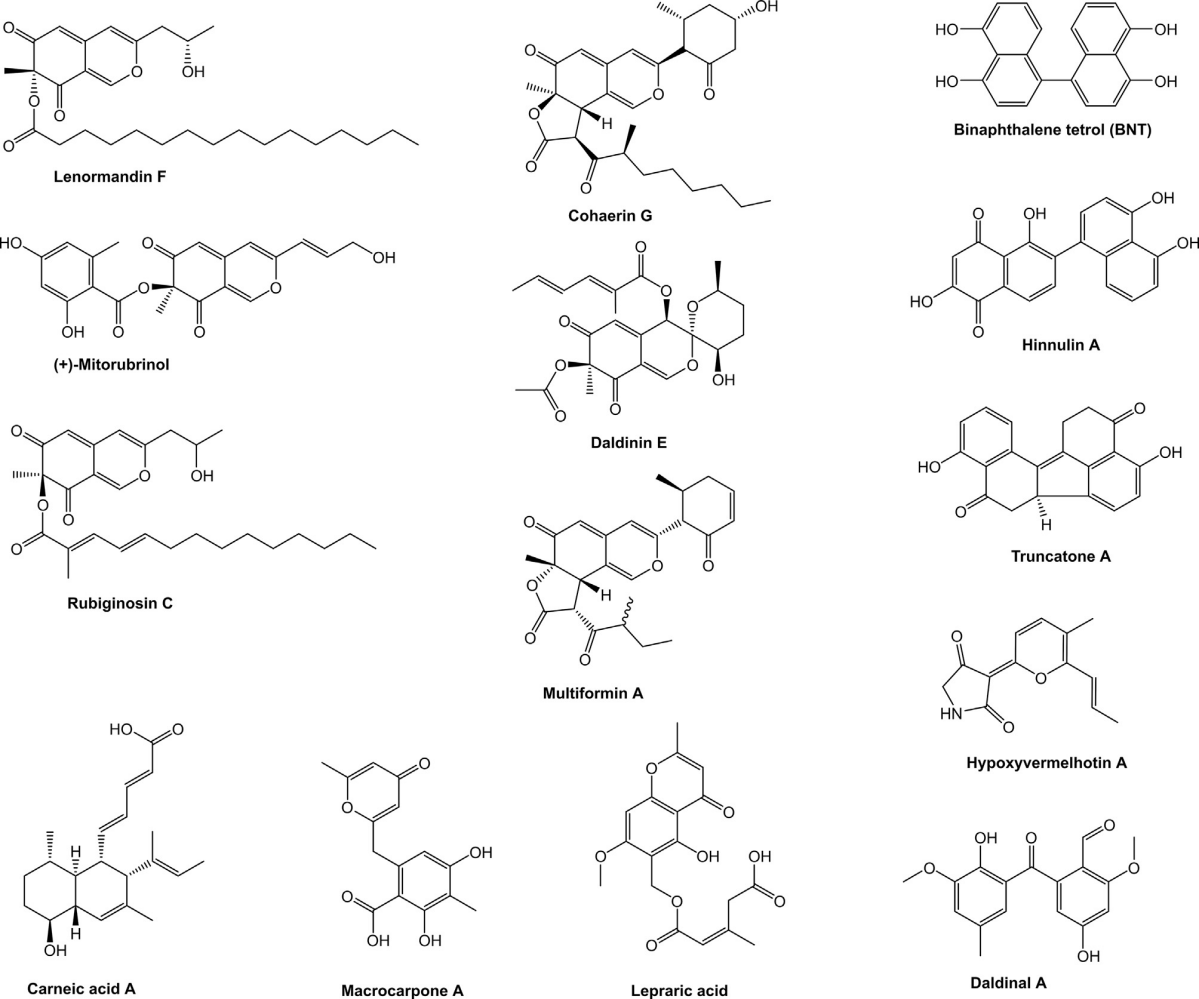
Structural diversity of pigments stored in the stromata of *Hypoxylaceae* species.

The *Xylariales* is one of the most diverse fungal orders in the *Sordariomycetes* with a world-wide distribution that currently comprises 22 families, and 56 genera with uncertain placement (Wijayawardene 2020). The largest family by far in the *Xylariales* is the *Xylariaceae*, which has been recently segregated into various families based on polyphasic taxonomic concepts (Wendt *et al.* 2018, Daranagama *et al.* 2018). The *Hypoxylaceae* is one of the families that have been established by this approach and this family includes species that often form ascomata embedded into stromatal tissue on dead wood. Additionally, many members of the family are characterised by the presence of a layer of pigment granules surrounding the perithecia which can be dissolved upon treatment with potassium hydroxide solution (KOH) to give species-specific colour reactions, which is an important character for species discrimination (Wendt *et al.* 2018). Systematic analysis of the KOH-extractable pigments by chromatographic and structure elucidation methods has revealed an unprecedented diversity of secondary metabolites, the occurrence of which can significantly vary between the investigated species (Hellwig *et al.* 2005, Helaly *et al.* 2018). Depending on the species, up to more than 20 different, but often related compounds can be observed in stromatal extracts, most of which belong to the classes of azaphilones or binaphthalenes (Becker *et al.* 2021a) (Fig. 1). Interestingly, the production of specific stromatal metabolites is highly conserved within a species, independent of its origin and environmental conditions making the analysis of secondary metabolite profiles a reliable tool for taxonomic purposes (Kuhnert *et al.* 2015c).

**Fig. 2:**
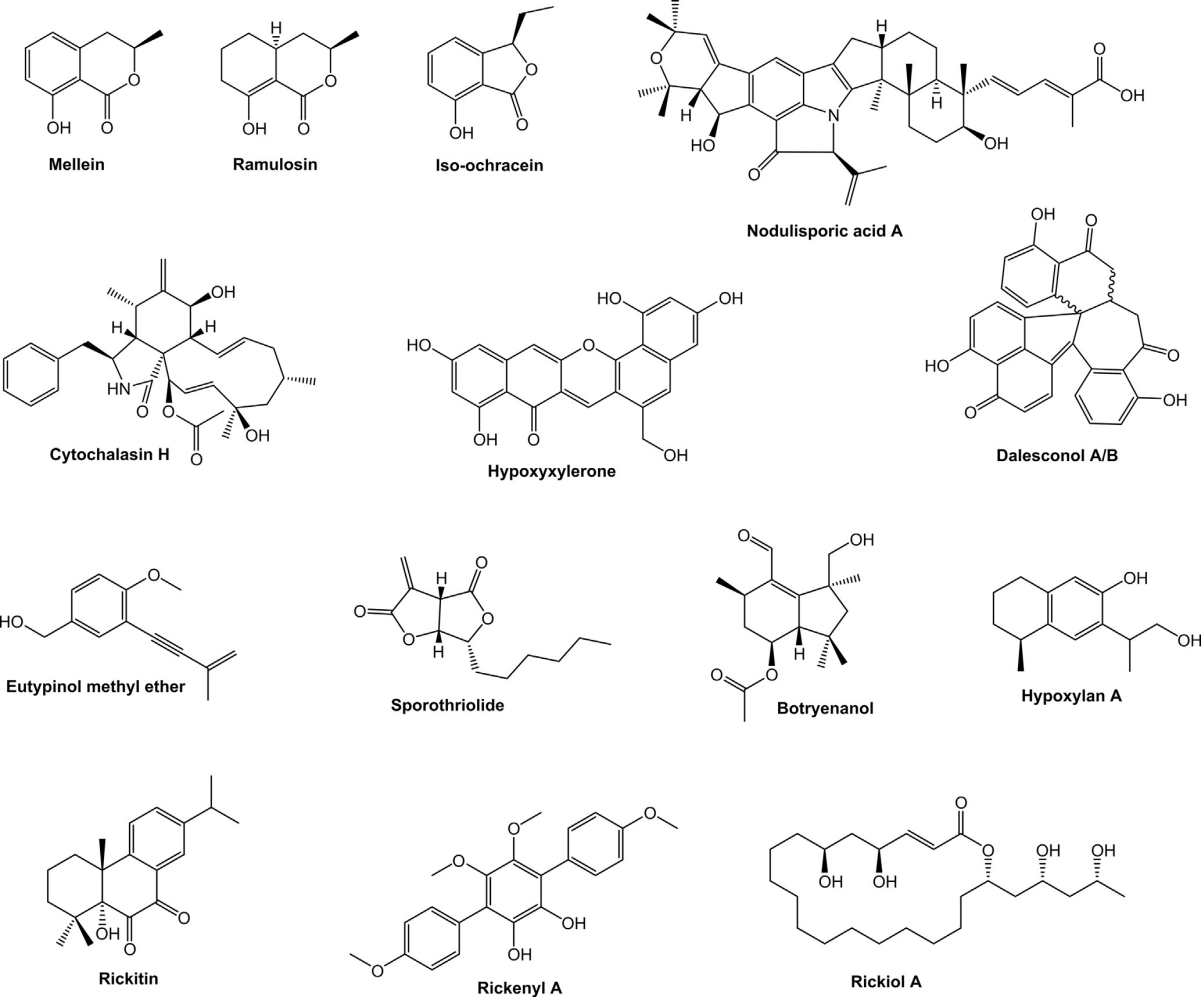
Structural diversity of of secondary metabolites produced in cultures of *Hypoxylaceae* species.

Characterisation of compounds produced by *Hypoxylaceae* species already started in the late 1950s with the isolation of binaphthalene tetrol (BNT) from the stromata of *Daldinia concentrica* and related monomeric naphthalenes from the respective cultures (Allport and Bu’Lock 1958, 1960). Later on, cultures of various species were systematically screened, revealing a diversity of simple polyketides such as mellein, ramulosin and iso-ochracein (Anderson *et al.* 1983). With increasing interest in endophytic fungi and their assumed potential for the production of medically relevant compounds, *Hypoxylaceae* taxa were frequently encountered in respective screenings. In combination with our efforts to systematically survey stromatal pigments of family members more than 200 different natural products have been characterised from the family so far with many of them showing interesting biological activities (Helaly *et al.* 2018, Becker & Stadler 2021). A prominent example are the nodulisporic acids, a group of indole diterpenoids with strong insecticidal activity produced by *Hypoxylon pulicicidum* that have been developed into a drug candidate for veterinary medicinal applications (Bills *et al.* 2012). Other compounds such as the fatty acid derived antifungal sporothriolides from *Hypomontagnella* spp. (Surup *et al.* 2014, Tian *et al.* 2020), various potent cytotoxic cytochalasans from *H. fragiforme* and *Daldinia* spp. (Ondeyka *et al.* 1992, Stadler *et al.* 2001a, Wang *et al.* 2019b), the topoisomerase I inhibitor hypoxyxylerone from *H. fragiforme* (Piettre *et al.* 2002), the immunosuppressive polyketides dalesconols A and B from *D. eschscholtzii* (Zhang *et al.* 2008), and phytotoxic eutypine derivatives from *Phylacia sagrana* (Bitzer *et al.* 2008) further add to the structural diversity of biosynthesised natural products in this family (Fig. 2). While many of the investigated strains were only superficially analysed for the presence of new compounds, often as part of bioactivity guided screening approaches, a more thorough investigation of *Hypoxylon rickii* demonstrated the hidden potential of the family members. A 70 L bioreactor fermentation yielded thirty-one compounds derived from eight different core scaffolds including various sesquiterpenoids, a diterpene, macrolactones, terphenyls and aromatic polyketides (Kuhnert *et al.* 2015a,b, Surup *et al.* 2015, 2018a, Wiebach *et al.* 2016) (Fig. 2). The same fungus also contains various mitorubrin-type azaphilones in its stromata, which are not produced under laboratory conditions (Kuhnert *et al.* 2015a).

The extraordinary diversity of the *Hypoxylaceae* in terms of secondary metabolite production raised the question about how much of it has been uncovered. Most enzymatic assembly lines responsible for the formation of individual molecules in fungi are encoded by so-called biosynthetic gene clusters (BGCs), *i.e.* genes involved in the biosynthesis of the same compound are located in close proximity to each other and are often co-regulated (Keller 2019). Thus, they can be identified by systematic genome-mining approaches, giving a good estimation of the production capabilities of a given fungal strain. In addition, the majority of known natural products are derived from a few common structurally conserved genes, such as those encoding polyketide synthases (PKS), nonribosomal peptide synthetases (NRPS), terpene cyclases and hybrid synthases (PKS-NRPS, NRPS-PKS). In most cases these core genes can be easily located by homology searches once a genome sequence is available (Keller 2019). Due to the significance of secondary metabolites for survival and adaptation (Spiteller 2015), investigating the diversity of biosynthetic pathways also gives insights in the complexity of chemical interactions of the *Hypoxylaceae* with their environment. Even though hundreds of species have been described to date, little is known about these fungi in this regard.

We recently obtained the genome sequences of 13 representatives of the *Hypoxylaceae* including *Annulohypoxylon truncatum*, *Daldinia concentrica*, *Daldinia* sp. (deposited as *Entonaema liquescens*), *Hypomontagnella monticulosa*, *Hypom. spongiphila*, *Hypom. submonticulosa*, *Hypoxylon fragiforme*, *H. lienhwacheense*, *H. pulicicidum*, *H. rickii*, *H. rubiginosum*, *Jackrogersella multiformis* and *Pyrenopolyporus hunteri* as well as the related species *Xylaria hypoxylon* (*Xylariaceae*), covering most of the family lineages according to a polyphasic study by Wendt *et al.* (2018). Various comparative genomic approaches were used to analyse the basic gene level differences between the species showing that even closely related species differ significantly in their gene content (Wibberg *et al.* 2021). Due to the high quality of the genome sequences (with N50 values between 1.2 Mbp and 5.0 Mbp and a contig count ranging from 16 to 88), the data offered a very good opportunity to obtain a comprehensive picture about the biosynthetic arsenal encoded by the selected *Hypoxylaceae* members based on our current understanding of fungal biosynthesis, which we attempted herein. Additionally, we aimed to link as many BGCs as possible with known or predicted products either based on experimental evidence or homology analysis with other already characterised fungal biosynthetic pathways. We complemented the study by secondary metabolite screening approaches using a selection of 10 different media to identify predicted pathway products.

## Material & methods

### Strains and genome sequences

Previously acquired genome sequences of 13 *Hypoxylaceae* species and one *Xylaria hypoxylon* strain were analysed (Wibberg *et al.* 2021). Details about the strains and respective genome sequences are listed in Table 1. The strain ATCC 46302 is deposited as *Entonaema liquescens* but the genome sequence raised serious concern about the authenticity (see Wibberg *et al.* 2021 for detailed discussion on this subject). Due to its phylogenetic affinity with the genus *Daldinia* the strain is herein referred to as *Daldinia* sp.

**Table 1:** Details of strains and respective genome sequences of selected *Xylariales* analysed in this study. ILU – Illumina, ONT – Oxford Nanopore Technology, PB – PacBio. Reproduced from Wibberg *et al.* (2021).

Organism	Strain	Sequencing method	Genome size [bp]	Contigs	N50 [bp]	Annotated genes^1^	GC [%]
*Annulohypoxylon truncatum*	CBS 140778	ONT/ILU	38 511 861	64	1 760 563	11 384	46.5
*Daldinia concentrica*	CBS 113277	ONT/ILU	37 605 921	69	2 728 111	11 205	43.8
*Daldinia sp. (“Entonaema liquescens”)*	ATCC 46302	ONT/ILU	39 197 785	31	3 541 465	10 384	43.4
*Hypomontagnella monticulosa*	MUCL 54604	ONT/ILU	42 889 121	30	3 439 634	12 475	46.0
*Hypom. spongiphila*	MUCL 57903	ONT/ILU	42 173 915	16	5 039 066	12 622	46.2
*Hypom. submonticulosa*	DAOMC 242471	ONT/ILU	41 374 079	123	657 615	11 692	46.3
*Hypoxylon fragiforme*	MUCL 51264	PB	38 198 373	36	3 581 784	10 557	46.2
*H. lienhwacheense*	MFLUCC 14-1231	ONT/ILU	35 785 595	61	1 602 745	9 942	45.4
*H. pulicicidum*	ATCC 74245	ONT/ILU	43 543 700	24	3 855 590	12 174	44.8
*H. rickii*	MUCL 53309	ONT/ILU	41 846 710	81	3 963 481	11 101	46.4
*H. rubiginosum*	MUCL 52887	PB	48 274 337	70	1 165 420	13 276	44.1
*Jackrogersella multiformis*	CBS 119016	ONT/ILU	38 501 162	20	4 087 316	11 271	45.8
*Pyrenopolyporus hunteri*	MUCL 49339	ONT/ILU	40 356 773	50	2 611 040	9 720	44.9
*Xylaria hypoxylon*	CBS 122620	ONT/ILU	54 341 593	88	3 886 849	12 704	40.7

1 As predicted by the GeneMark tool

### Biosynthetic gene cluster identification and analysis

For BGC prediction and annotation we applied a manual search strategy based on the Blastp algorithm (Altschul *et al.* 1997) using template protein sequences of known biosynthetic core enzymes against protein databases of the targeted organism. Blastp searches were conducted with the software package Geneious v. 9.1.8 using standard parameters (Max E-value 1e-1, BLOSUM62 matrix, gap cost 11 1). The respective template protein sequences to target polyketide, peptide, terpene, alkyl citrate, alkaloid and hybrid pathways are shown in Table 2. Genomic loci containing predicted biosynthetic core genes were further analysed for the presence of putative co-localised biosynthetic genes including tailoring, regulatory, transport and resistance genes, whose functions were predicted by Blastp homology search of the encoded protein sequences against the Swiss-Prot database (Boeckmann *et al.* 2003). Borders of BGCs were defined when the encoded proteins of three consecutive genes did not show similarity to known BGC-related enzymes in the Swiss-Prot database. The identified BGCs can be accessed under 10.6084/m9.figshare.14534784.

**Table 2:** Templates used for the identification of biosynthetic core genes.

Protein Acc. No.	Protein name	Organism	BGC target	Reference
APH07629	orsellinic acid synthase PKS1	*Agaricomycetes* sp.	type I PKS, PKS-NRPS	(Braesel *et al.* 2017)
XP_960427	chalcone synthase	*Neurospora crassa*	type III PKS	(Galagan *et al.* 2003)
S3DQP3	A1 domain of nonribosomal peptide synthetase GloA	*Glarea lozoyensis*	NRPS, NRPS-like, PKS-NRPS, NRPS-PKS	(Chen *et al.* 2013)
AHY23922	1,8-cineole synthase	*Hypoxylon* sp.	Terpene	(Shaw *et al.* 2015)
AWM95795	humulene synthase Asr6	*Sarocladium* sp.		(Schor *et al.* 2018)
Q6WP50	presilphiperfolan-8-beta-ol synthase Bot2	*Botrytis cinerea*		(Pinedo *et al.* 2008)
QOE88883	brasilane synthase BraA	*Annulohypoxylon truncatum*		(Feng *et al.* 2020)
C9K2Q3	fusicoccadiene synthase	*Alternaria brassicicola*		(Minami *et al.* 2009)
A0A1B4XBG5	cycloaraneosene synthase SdnA	*Sordaria araneosa*		(Kudo *et al.* 2016)
B2DBF1	copalyl diphosphate synthase	*Diaporthe amygdali*		(Toyomasu *et al.* 2008)
P38604	lanosterol synthase Erg7	*Saccharomyces cerevisiae*		(Corey *et al.* 1994)
QOV03404	citrate synthase SpoE	*Hypomontagnella monticulosa*	Alkyl citrate	(Tian *et al.* 2020)
D4D449	tryptophan dimethylallyltransferase	*Trichophyton verrucosum*	Alkaloid	(Wallwey *et al.* 2012)
AMR44282	phomopsin precursor PhomA	*Diaporthe leptostromiformis*	RiPP	(Ding *et al.* 2016)
n/a	omphalotin precursor OphMA	*Omphalotus olearius*		(Ramm *et al.* 2017)
ACB30126	epichloëcyclin precursor GigA	*Epichloë festucae*		(Johnson *et al*. 2015)

### Homology analysis of biosynthetic gene clusters

For the identification of homologous published BGCs, a Blastp analysis of the core enzymes of the template BGCs against the Swiss-Prot database was performed under standard settings, and BGC information of the best hits was retrieved from public databases [NCBI, MIBiG (Kautsar *et al.* 2019)]. In cases where fully annotated BGCs were not deposited in public databases, the information was directly acquired from the respective genome sequences. BGC synteny was assessed by the Artemis comparison tool (Carver *et al.* 2005) which uses the tblastx algorithm, and clinker (Gilchrist & Chooi 2021), which compares the sequence similarity of the encoded proteins. Homology analysis between multiple BGCs was conducted with clinker. Visualisation of synteny was achieved with clustermap.js which is implemented in the clinker tool (Gilchrist & Chooi 2021). Large scale identification of homologous BGCs across annotated fungal genomes stored in the NCBI genome databases was accomplished with the cblaster tool (Gilchrist *et al.* 2021).

### Networking analysis

The identified and manually curated BGCs (except type III PKS BGCs) of the 14 fungal genomes were used for creating a BGC similarity network. This dataset was complemented with characterised fungal BGCs from the MIBiG database (v. 2.0) (Kautsar *et al.* 2019), as well as characterised BGCs from literature that are not yet in the database. All input BGCs were annotated by antiSMASH v. 5.1.2 (Blin *et al.* 2019).

BiG-SCAPE v. 1.0 was used to create the similarity networks (Navarro-Muñoz *et al.* 2020). Briefly, all-*vs*-all cluster similarity is calculated per major biosynthetic class in the form of distance, which is a combination of three indices (Jaccard Index, JI; Domain Sequence Identity, DSI, and Adjacency Index, AI) using the set of predicted domains in each BGC. Only distances below a specified cutoff distance are kept. Similarity distances range from 0 (complete similarity) to 1 (not similar at all). Finally, a clustering algorithm is applied to all resulting subnetworks.

BiG-SCAPE was run with version 34 of the Pfam database (Mistry *et al.* 2021) and four domains added to the "anchor_domains" file (PF00285, Citrate_synt; PF19086, Terpene_syn_C_2; PF01040, UbiA and PF11991, Trp_DMAT). The domains in this file are given extra weight during calculation of the DSI index. Four cutoff values were used: 0.3 (default), 0.4, 0.5 and 0.6. Other parameters used: --include_singletons --mix --clans-off --mode global. The last option makes BiG-SCAPE employ all domains in each BGC-pair similarity calculation. This will make results more sensitive but was chosen due to the input BGCs being curated and fungal BGCs being more likely to present gene rearrangements that might interfere with the extension algorithm in glocal mode.

The clustering step on subnetworks in BiG-SCAPE was designed to deal with over-connectedness of prokaryote BGCs having a high density of genes with similar domain content. As this is not the case in fungal BGCs, the analysis of the present dataset focused on the created subnetworks to prevent legitimate groups of similar clusters to be broken down by the clustering step. Therefore, in this work we used subnetworks as gene cluster families (GCF). The main analysis was focused on the "mix" class which includes all BGC types (index weights: JI=0.2, DSI=0.75, AI=0.05). Finally, all subnetworks comprised only by characterised BGCs were pruned from the network files.

Networks were visualised with cytoscape (v. 3.7.1, https://cytoscape.org/). A table containing information about the subnetworks is provided in the Supplementary Information (Network Files).

### Phylogenetic analysis

For phylogenetic reconstruction of the FAD-dependent monooxygenase (FMO) involved in azaphilone biosynthesis, the respective protein sequences from the *Hypoxylaceae* genomes were aligned with 23 characterised FMO and four TropB homologs identified in the *Hypoxylaceae* tropolone BGCs using MAFFT v. 7.407 [--reorder, (Katoh 2002)]. A detailed list of the used FMO sequences can be found in the Supplementary Information (Table S1). Poorly aligned regions were removed using trimAl v. 1.2rev59 [-automated1, (Capella-Gutierrez *et al.* 2009)]. A maximum likelihood phylogenetic tree was built using IQ-TREE v. 1.6.8 [-m MFP -bb 1000 -alrt 1 000 -abayes -nt AUTO, (Nguyen *et al.* 2015)]. Protein model was selected by ModelFinder (Kalyaanamoorthy *et al.* 2017). Branch support was calculated with the ultrafast bootstrap approximation (Hoang *et al.* 2018), Shimodaira-Hasegawa-like approximate likelihood ratio test (Guindon *et al.* 2010), and approximate Bayes test (Anisimova *et al.* 2011). The tree was visualised and annotated with iTOL (Letunic & Bork 2019). The FAD-dependent urate oxidase from *Klebsiella pneumonia* (UniProtKB/Swiss-Prot: A6T923.1) was used as outgroup.

Phylogenetic analysis of NRPS-like enzymes was conducted with the protein sequences of 16 enzymes identified from the *Hypoxylaceae* genomes and ten sequences of previously characterised NRPS-like proteins retrieved from the Swiss-Prot database, a detailed list of which can be found in the Supplementary Information (Table S2). Sequence alignment and calculation of the phylogenetic tree was performed as described for the FMO. The piperazine synthase LnaA from *Aspergillus flavus* (UniProtKB/Swiss-Prot: B8NTZ9.1) was used as outgroup.

Homologues of the Ace1 PKS-NRPS hybrid from *Pyricularia oryzae* Guy11 were retrieved from the NCBI nr database using Blastp search (default parameters). Sequences annotated as partial were not included, as well as sequences with large insertions or deletions. The EqxS hybrid from *Fusarium heterosporum* and ACLA_023380 from *Aspergillus clavatus* were included as known outgroup (Khaldi *et al.* 2008). Selected sequences from *Colletotrichum higginsianum*, *Chaetomium globosum*, *Daldinia eschscholtzii* EC12 from JGI MycoCosm (Grigoriev *et al.* 2014) were also included because of previous phylogenetic analyses (Khaldi *et al.* 2008, Moore *et al.* 2014). Sequence alignment and calculation of the phylogenetic tree was performed as described for the FMO.

All trimmed alignments and tree files are provided in the Supplementary Information (Alignments and tree files).

### Screening and secondary metabolite analysis

For analysis of the secondary metabolites produced by the strains (except for *H. rubiginosum, Hypom. spongiphila,* and *Hypom. submonticulosa*) seven liquid and three solid growth media were used. Details about the media and their composition can be found in the Supplementary Information (Table S3). Initially, seed cultures of each strain were grown in 250 mL shaking flasks containing 50 mL SMY-A medium at 220 rpm and 25 °C in an incubation shaker. Screening was conducted in 60 mL glass tubes (Rotilabo®-screw neck ND24 vials, SN LC94.1, Carl Roth GmbH, Karlsruhe, Germany), which were sealed with apertured screw caps (SN 29 227 09 02, Duran group, Mainz, Germany) covered with PTFE membranes (SN PM3010, Porex Technologies, Fairburn, GA/USA). From each medium, 12 mL were transferred into the tubes and then autoclaved (if not stated otherwise in Table S3). Inoculation was accomplished by adding 0.5 mL of seed culture to the tubes. Submerged cultures were incubated at 220 rpm and 23 °C with a 30° inclination using inclinable racks. Solid cultures were incubated under the same conditions, but omitting agitation. The fermentation was terminated after 10 d.

Secondary metabolites were extracted by adding 12 mL of methylethylketone (MEK) to each tube (solid media were disrupted in advance) and shaking the mixtures at 220 rpm for 3 h. Extracts from solid media were first filtered by vacuum filtration to remove debris followed by addition of 12 mL of distilled H_2_O and MEK each. After phase separation, the organic phase was gradually transferred to brown glass vials and dried under nitrogen at 40 °C. Remaining water was removed by lyophilisation and the crude extracts were dissolved in 1 mL of dimethylsulfoxide (DMSO).

Samples were analysed by HPLC-HRMS using an Agilent 1200 Infinity Series HPLC (Agilent Technologies) coupled to a maXis ESI-TOF-MS (Bruker). For HPLC, a C18 Acquity UPLC BEH column (50 × 2.1 mm, 1.7 mm; Waters) was used as stationary phase. The mobile phase was composed of H_2_O + 0.1 % formic acid (solvent A) and acetonitrile + 0.1 % formic acid (solvent B) applying the following gradient: 5 % B for 0.5 min, increasing to 100 % B over 19.5 min, isocratic conditions for 5 min. The flow rate was adjusted to 0.6 mL/min and UV/vis absorption was measured in a range of 190–600 nm. MS parameters were set as follows: scan range: 50−2 500 m/z, ion polarity: positive, capillary voltage: 4 500 V, nebuliser pressure: 4.0 bar, dry heater: 200 °C, dry gas flow: 10 L/min, collision energy: 10 eV.

## Results

### Biosynthetic classes in the *Hypoxylaceae*

In order to identify as many biosynthetic pathways as possible in the genomes of the selected *Hypoxylaceae* and *X. hypoxylon*, a manual search strategy was applied based on sequence similarity. We specifically looked for the following types of core proteins by using appropriate search templates: polyketide synthases (PKS); nonribosomal peptide synthetases (NRPS) or NRPS-like enzymes; PKS-NRPS and NRPS-PKS hybrid systems; mono-, sesqui-, di- and triterpene cyclases; citrate synthases, tryptophan dimethylallyltransferases; and ribosomally synthesised and post-translationally modified leader peptides (RiPP) (Table 2). Borders of BGCs were defined when no obvious biosynthetic genes could be located in the vicinity of the core gene. For simplicity reasons, loci only consisting of a core biosynthetic gene are also referred to as BGC. In some cases, tailoring genes can be located at a distant locus, which however, cannot be further evaluated in this study for BGCs with unknown product or without characterised homologous BGCs.

Within the 14 analysed genomes, we identified a total of 783 putative BGCs corresponding to an average of 56 BGCs per species (Table 3). The highest number of BGCs was found in *X. hypoxylon* (83) and lowest number in *H. lienhwacheense* (25). Unclustered NRPS-like enzymes with an adenylation (A), thiolation (T) and reductase (R) domain structure were excluded from the counting as it is not clear if they are part of secondary metabolism. For instance, each genome contained a highly conserved NRPS-like gene with A-T-R-R structure, homologs of which have already been characterised as glycine betaine reductase in a previous study (Hai *et al.* 2019), thus belonging to primary metabolism. Roughly half of the identified BGCs corresponds to polyketide pathways that can be further subdivided into nonreducing PKS (nrPKS), highly reducing PKS (hrPKS), partially reducing PKS (prPKS), collaborative PKS (BGCs with two or more PKS genes), type III PKS and truncated/disintegrated PKS (Cox 2007, Chooi & Tang 2012). HrPKS were the dominating type of PKS which are present in 69 % of all PKS BGCs.

**Table 3:**
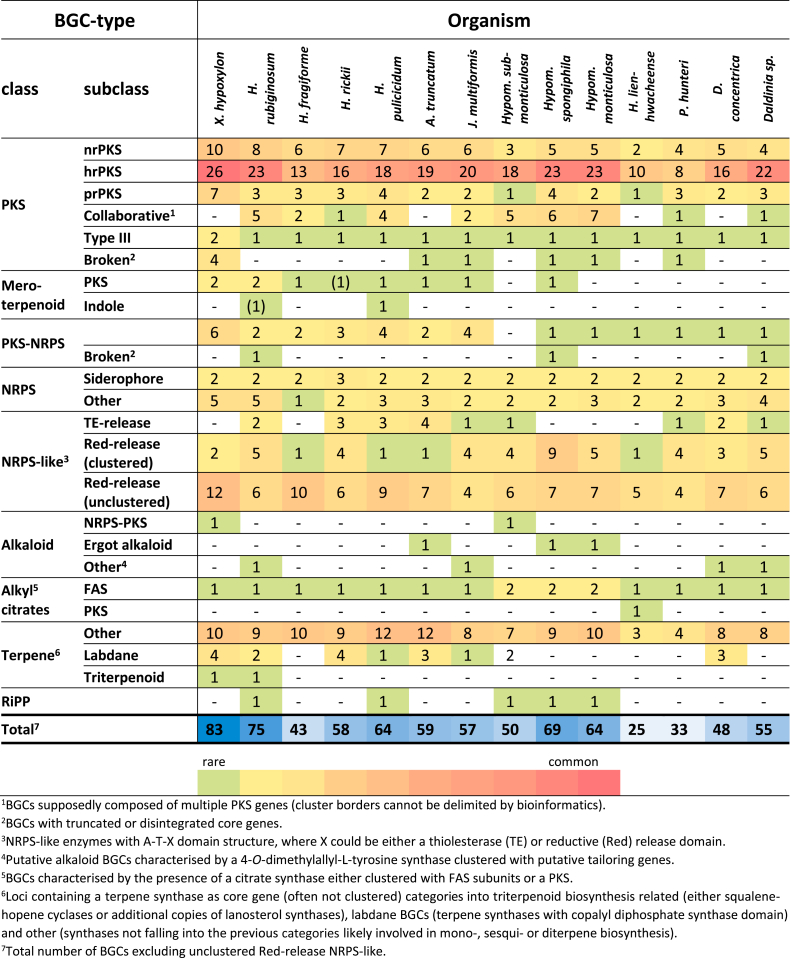
Classes of biosynthetic gene clusters (BGC) and number of representatives identified in the genomes of *X. hypoxylon* and selected *Hypoxylaceae* by manual genome mining. Frequency and total number of BGCs are colour coded (green to red – rare to common, light blue to dark blue – low number to high number).

Up to seven BGCs with co-localised PKS genes were identified per species (collaborative PKS). Biosynthetic pathways that require two PKS genes for product formation are frequently encountered in fungi and have been elucidated on multiple occasions including those responsible for azanigerone, asperfuranone, squalestatin and sorbicillin formation (Chiang *et al.* 2009, Zabala *et al.* 2012, Fahad *et al.* 2014, Bonsch *et al.* 2016). Only three collaborative PKS BGCs in our dataset could be assigned to specific product families (see azaphilone section for additional information) based on homology analysis. However, it is important to mention that individual BGCs can occur in close proximity to each other or do even intertwine as observed for the fumagillin/pseurotin supercluster in *Aspergillus fumigatus* (Wiemann *et al.* 2013). Therefore, the predicted collaborative PKS BGCs could also in some cases represent two independent BGCs.

In some genomes, apparently defective PKS genes that lacked several domains, often clustered with other biosynthetic genes (but no other core genes), were found. These presumably “dead” biosynthetic pathways were nevertheless included in the BGC count. Four of such truncated PKS pathways were for instance located in the *X. hypoxylon* genome. In addition, on a few occasions BGCs with disintegrated PKS genes were discovered, where a complete set of domains was distributed across various genes. Such a BGC was for example found in *X. hypoxylon*, where a hrPKS was split into two subunits with the first one carrying the complete KS, AT, DH, C-Met and ER domains and a second one (being located 8 kb downstream of the first partial PKS gene) containing the missing KR and ACP domains. There are currently no reports about split fungal PKS that retain functionality. Therefore, it cannot be deduced if such BGCs are able to form a product. Furthermore, each *Hypoxylaceae* genome contained a single copy of an unclustered type III PKS gene, while two unclustered copies were located in the *X. hypoxylon* genome.

PKS BGCs containing a prenyltransferase and/or terpene cyclase gene were assigned to meroterpenoid pathways (see meroterpenoid section for more details). Corresponding clusters were identified in *ca.* 50 % of the analysed genomes. In *H. lienhwacheense* a hrPKS gene was clustered with a citrate synthase and therefore assigned to alkyl citrate pathways. Related BGCs have been reported from *Byssochlamys fulva* and shown to be involved in the biosynthesis of maleidrides such as byssochlamic acid (Williams *et al.* 2016). Further highly conserved alkyl citrate pathways with unknown products, consisting of a citrate synthase encoded alongside dedicated fatty acid synthase components as core were found in all *Hypoxylaceae* genomes. Species of the genus *Hypomontagnella* contained an additional related cluster which we recently showed to be responsible for sporothriolide production in these taxa (Tian *et al.* 2020). *Xylaria hypoxylon* has a homologous BGC likely involved in piliformic acid biosynthesis (see alkyl citrate section for further details) (Tian *et al.* 2020).

All genomes except *Hypom. submonticulosa* contained at least one hybrid PKS-NRPS BGC with a maximum number of six BGCs in *X. hypoxylon* and a total number of 29 across all analysed genomes. The only known products of such pathways in the *Hypoxylaceae* and *X. hypoxylon* are cytochalasans, which can be correlated to their respective BGCs (see respective section). Except for a PKS-NRPS BGC from *J. multiformis*, which was highly similar to the curvupallide (*cpa*) BGC from *Curvularia pallescens* (Yokoyama *et al.* 2017) (Fig. S1), the majority of identified PKS-NRPS BGCs did not show similarity to known BGCs and therefore their products cannot be predicted.

The number of NRPS BGCs varies between two and seven with each strain possessing at least two siderophore BGCs. Siderophores were rarely observed under the standard screening conditions, but were found in large excess when beech chips were added to YMG medium (see Table S3 for recipe). Under these conditions, the siderophores coprogen (Keller-Schierlein & Diekmann 1970) and derivatives as well as dimerumic acid (Burt 1982) were produced by all screened strains except *X. hypoxylon* (Fig. S2). Between two and six modules are present in the individual NRPSs. No peptide except for diketopiperazines (Surup *et al.* 2015) have been reported from the *Hypoxylaceae* to date, preventing prediction of the BGC products. In addition, various NRPS-like BGCs were found in the genomes. Two types of NRPS-like enzymes were distinguished. The first type has a characteristic A-T-TE domain architecture where the release of the product is catalysed by the thiolesterase (TE) domain. Related pathways are known to be responsible for the formation of terphenyls and indolequinones and can be correlated with known compounds produced by the *Hypoxylaceae* (see section “Compounds derived from NRPS-like enzymes”) and up to four different BGCs can be found in the studied species. The second type features an A-T-R domain structure where R refers to a reductive release mechanism. These types of enzymes are located in some PKS BGCs of the *Hypoxylaceae* where they likely function as carboxylic acid reductases, a reaction that has already been described for various PKS biosynthetic pathways (Araki *et al.* 2019, Huang *et al.* 2020). In addition, NRPS-like genes can form individual BGCs as it has been demonstrated for the biosynthesis of piperazine alkaloids, such as herquline and related compounds (Forseth *et al.* 2013, Yu *et al.* 2016), or mycosporine-like amino acids (Miyamoto *et al.* 2014). Homologs of the latter BGCs can be found in most *Hypoxylaceae* genomes, in addition to other uncharacterised NRPS-like BGCs.

Search for alkaloid BGCs revealed the presence of a few pathways, two of which contain a NRPS-PKS core gene (see swainsonine section for details). In addition, ergot alkaloid BGCs were located in the genomes of *A. truncatum*, *Hypom. monticulosa* and *Hypom. spongiphila* with the *A. truncatum* BGC being characterised by the presence of two NRPS genes (see ergot alkaloid section for details). In four cases a tryptophan dimethylallyltransferase (DMAT) was clustered with other biosynthetic genes, such as various types of monooxygenases and oxidoreductases. Even though the products of these pathways cannot be deduced, we predict them to be alkaloids.

The number of identified terpene BGCs significantly varied between the organisms and ranged from 15 in *X. hypoxylon* and *A. truncatum* down to three in *H. lienhwacheense*. In general, it is not possible to distinguish mono- and sesquiterpene cyclases due to their structural similarities. At least half of these identified terpene cyclases were not surrounded by obvious tailoring genes, indicating that either these genes are at a distant locus or the respective enzyme products are not modified and these systems may well be involved in the production of volatiles.

For diterpene cyclases, two different mechanisms of cyclisation are known, which correspond to their chemical structures. Monofunctional diterpene cyclases resemble sesquiterpene cyclases but can sometimes be coupled to a geranylgeranyl pyrophosphate synthase (GGPPS) as shown for ophiobolin biosynthesis (Schmidt-Dannert 2015). Genes encoding GGPPS-coupled diterpene synthases were found in *H. fragiforme*, *H. pulicicidum* and *H. rubiginosum,* all of which were not surrounded by tailoring genes. Bifunctional diterpene cyclases mediate terpene cyclisation *via* a bicyclic copalyl (labdane) intermediate and are for instance involved in the gibberellin or pleuromutilin assembly (Schmidt-Dannert 2015). As the latter type of diterpene cyclases is structurally distant from other known terpene synthases, it can be easily recognised and is therefore listed separately. The presence and diversity of labdane pathways were species dependent. For instance, four labdane BGCs were located in the genome of *H. rickii*, while the closely related fungus *H. fragiforme* lacked respective BGCs. Their occurrence also varied in the genus *Hypomontagnella*, where *Hypom. submonticulosa* had two BGCs and *Hypom. monticulosa* and *Hypom. spongiphila* were devoid of such.

For triterpenoid pathways, two types of core enzymes are reported in fungi to date. The first type corresponds to lanosterol synthases, which use oxidosqualene to form the tetracyclic lanosterol or structurally related backbones as found in helvolic acid and fusidic acid (Hu *et al.* 2020), while fernane synthases use the same substrate to yield pentacyclic fernanes, the precursor of compounds such as enfumafungin or polytolypin (Kuhnert *et al.* 2018). Fungi contain at least one copy of the lanosterol synthase gene as its product is required for ergosterol biosynthesis. Herein, the ergosterol pathway is assigned to primary metabolism as it is an essential structural component of the cell membrane, and it was therefore excluded from the list (Hu *et al.* 2020). Identification of other triterpene pathways thus relies on the presence of additional triterpene cyclase genes in predicted BGCs. This was only the case in the genomes of *X. hypoxylon* and *H. rubiginosum* (see triterpene section for details).

A putative RiPP BGC with similarity to the ustiloxin BGC (*ust*) (Ding *et al.* 2016) was found in all *Hypomontagnella* species, *H. rubiginosum* and *H. pulicicidum* (Fig. S3). As studies about fungal RiPPs are still scarce, the identification of RiPP pathways depends on the similarity of the precursor peptide sequences with those that have already been characterised. Therefore, it is currently not possible to estimate if the identified RiPP BGCs cover the actual diversity of RiPP pathways in the *Hypoxylaceae* and *X. hypoxylon*.

**Fig. 3:**
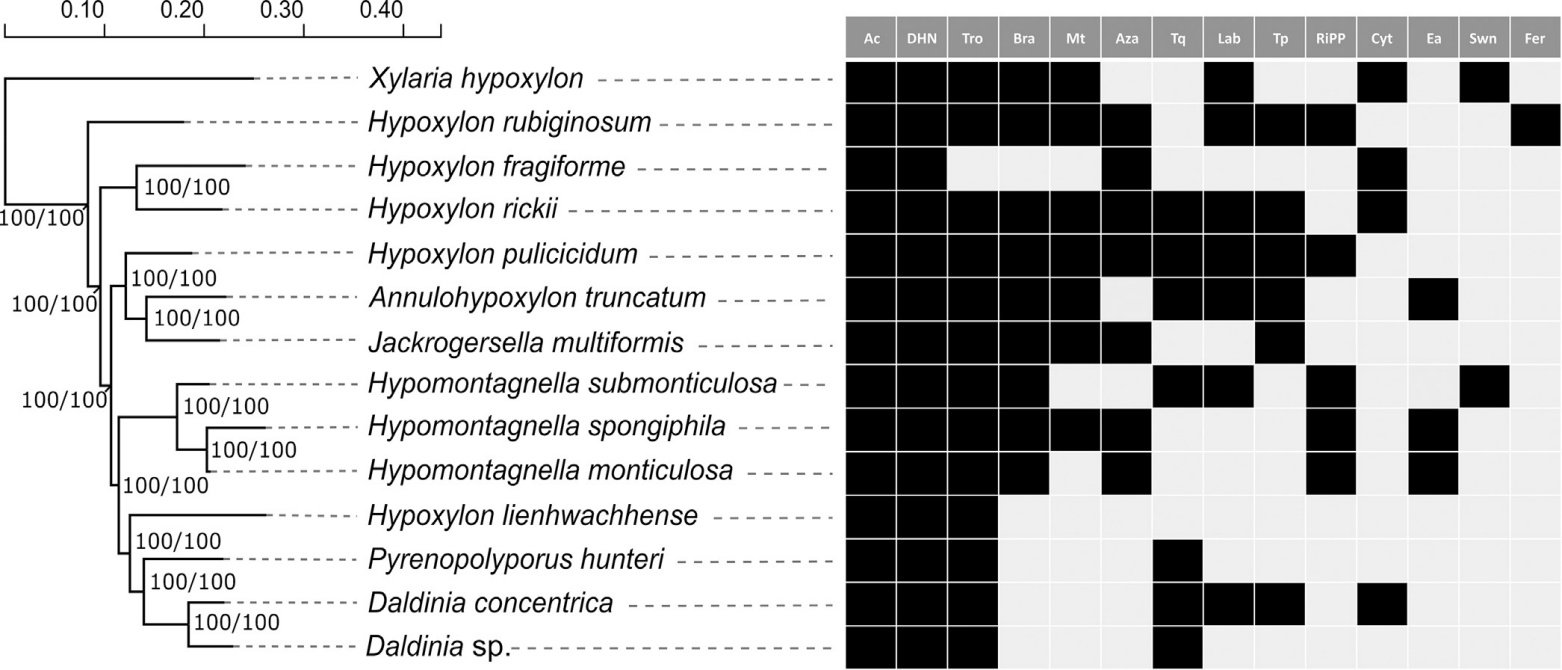
Absence/presence matrix of selected biosynthetic families across the *Hypoxylaceae*. Phylogenomic maximum likelihood tree of the *Hypoxylaceae* and *Xylaria hypoxylon* inferred from a protein sequence supermatrix approach of 4 912 curated concatenated protein alignments (replicated from Wibberg *et al.* 2021). Bootstrap and SH-aLRT support values of 1 000 replicates are given on nodes for the inferred consensus tree. Scale bar indicates nucleotide substitution rates. The right panel indicates the occurrence of selected biosynthetic gene cluster (BGC) families in the sequenced species. Ac: alkyl citrate BGC, Aza: azaphilone BGC, Bra: brasilane sesquiterpenoid BGC, Cyt: cytochalasan BGC, DHN: dihydroxynaphthalene BGC, Ea: ergot alkaloid BGC, Fer: fernane-type triterpenoid BGC, Lab: labdane-type BGC, Mt: meroterpenoid BGC, RiPP: ribosomally synthesised and post-translationally modified peptide BGC, Swn: swainsonine NRPS-PKS BGC, Tp: terphenyl or other NRPS-like BGC with A-T-TE domain structure other than terrequinone, Tq: terrequinone-type NRPS-like BGC, Tro: tropolone BGC.

We mapped the distribution of various families of BGC against the phylogenomic tree of analysed taxa showing that they are rarely conserved (Fig. 3). Often pathways appear in multiple linages of the *Hypoxylaceae* but then can be restricted to certain species within a lineage as seen for terrequinone, azaphilone and cytochalasan BGCs. Some BGC families such as the fernane, ergot alkaloid and swainsonine BGC are rare in our dataset, but in some cases there are indications that they will turn out to be more common, once more genome sequences of other family members are available (see ergot alkaloid section). In addition, previous population studies of the important human pathogen *Aspergillus fumigatus* have shown that the presence or absence of certain BGCs (*e.g.* helvolic acid, fumiquinazoline, various uncharacterised pathways) is a population or even strain-dependent phenomenon (Lind *et al.* 2017). Therefore, it cannot be deduced whether our analysed isolates are typical representatives of the respective species in terms of BGC content. Further strains of the particularly common species need to be genome-sequenced in the future, in order to properly address this question.

### BiG-SCAPE network analysis

**Fig. 4:**
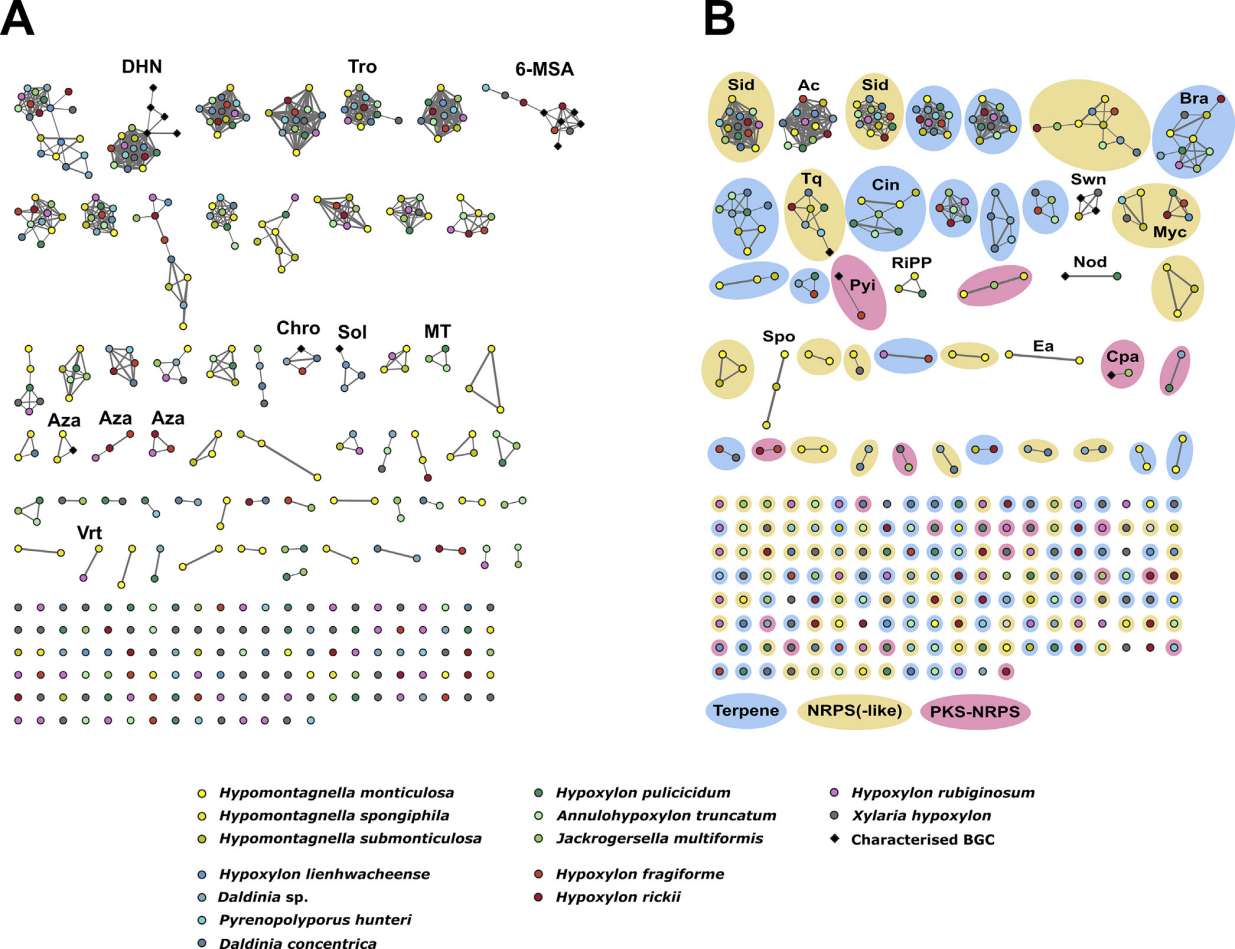
Gene cluster family (GCF) network of the 768 identified biosynthetic gene cluster (BGC) from 13 *Hypoxylaceae* genomes and *Xylaria hypoxylon* calculated by the BiG-SCAPE pipeline (cutoff value 0.4) and visualised with Cytoscape. PKS GCFs are shown on the left (A) and non-PKS GCFs are shown on the right (B). Each dot represents an identified BGC (including unclustered core genes). Characterised reference BGCs were included in the analysis (black rhombs). All BGC pairs with a distance equal or lower than the cutoff value are connected with links (thickness proportional to closeness). The layout of the figure was created according to Cytoscape's default layout algorithm ('Prefuse Force Directed Layout'), with the largest subnetworks at the top, and 'singletons', potentially unique BGCs not linked to any other BGC, shown at the bottom. The order of the singletons is random. Colors refer to species with closely related species having similar color codes. Known or predicted GCFs are labelled (6-MSA: 6-methylsalicyclic acid, Ac: alkyl citrate, Aza: azaphilone, Bra: brasilane, Chro: chromane, Cin: 1,8-cineole, Cpa: curvupallide, DHN: dihydroxynaphthalene, Ea: ergot alkaloid, MT: meroterpenoid, Myc: mycosporine-like, Nod: nodulisporic acid, Pyi: pyrichalasine, RiPP: ribosomally synthesised peptides, Sid: siderophore, Sol: solanapyrone, Spo: sporothriolide, Swn: swainsonine, Tq: terrequinone, Tro: tropolone).

To estimate the number of different biosynthetic pathways that are encoded by the 768 identified BGCs (excluding type III PKS), a gene cluster family (GCF) networking analysis using the BiG-SCAPE pipeline (Navarro-Muñoz *et al.* 2020) was conducted. BiG-SCAPE groups BGCs into GCFs based on the similarity of the predicted domain structures of the encoded proteins and visualises distances by similarity networks (see Network Analysis method section for details). 317 literature known fungal BGCs, stored in the MIBiG database (279) or manually extracted (38, labelled as FNP in the Supplementary Information), were included to infer relationships with known pathways. In total, 375 GCFs were predicted with 277 singletons (Fig. 4). Forty GCFs were comprised of at least four BGCs and ten GCFs contained previously characterised pathways.

Within the PKS dataset (Fig. 4A) most of the GCFs (172) could not be correlated to a specific product or product family. Among those PKS GCFs where product prediction is feasible is a large network composed of nrPKS BGCs known to be responsible for naphthalene formation (Fulton *et al*., 1999, Zhou *et al.* 2019) and their homologs from the *Hypoxylaceae* and *X. hypoxylon* genomes (see DHN section for detailed discussion). Another PKS GCF contained multiple literature-described prPKS involved in the biosynthesis of 6-methylsalicylic acid or mellein, and compounds derived thereof (Fujii *et al*., 1996, Lu *et al*., 2005, Chooi *et al.* 2015). These BGCs clustered with other prPKS pathways from *X. hypoxylon*, *H. fragiforme*, *H. rickii* and *P. hunteri*. Mellein derivatives are frequently encountered in cultures of *Hypoxylon* species (Bitzer *et al.* 2008) and were also identified in *X. hypoxylon* cultures by our screening, which suggests that the BGCs in this cluster could be involved in their formation.

Another PKS GCF was formed by three BGCs of *D. concentrica*, *Daldinia* sp. and *H. fragiforme* that were linked to a characterised chromane BGC of *Daldinia eschscholtzii*, which is part of the dalmanol biosynthetic pathway (Zhou *et al.* 2019). Chromanes, in particular 5-hydroxy-2-methyl-chromone, are reliably produced by *Daldinia* species (Bitzer *et al.* 2008) and it is therefore likely that the clustered BGCs are responsible for their biosynthesis. Related compounds were not found in cultures of *Hypoxylon* spp. and *X. hypoxylon* (Bitzer *et al.* 2008), which correlates well with the lack of chromane BGCs in these species. Furthermore, a BGC (*sol*) from the plant pathogen *Alternaria solani*, which encodes the biosynthesis of the phytotoxin solanapyrone (Kasahara *et al.* 2010), clustered with homologous BGCs from *D. concentrica*, *Daldinia* sp. and *H. lienhwacheense* (Fig. S4). While solanapyrones have not been reported from the *Hypoxylaceae*, a related molecule, dalsymbiopyrone, has been isolated from cultures of *D. hawksworthii* (Pažoutová *et al.* 2013). We identified dalsymbiopyrone in the screening extracts of all three species (Fig. S5) that for most parts can be explained by the *sol* BGC homologs. The biosynthesis of dalsymbiopyrone requires a polyketide chain with a different reduction pattern as the Sol1 product to prevent cycloaddition and a methylation step catalysed by an *O*-methyltransferase, which is not encoded in the BGCs of *Daldinia* sp. and *D. concentrica* (Fig. S4). However, a respective gene could be present on a different locus as homologs of the *O*-methyltransferase Sol2 are encoded throughout both genomes.

The final characterised PKS BGC that appeared in the PKS network map is the trigazaphilone BGC from *Trichoderma guizhouense* (Pang *et al.* 2020), which formed a GCF with a BGC from *Hypom. monticulosa* and *Hypom. spongiphila* (see azaphilone section for further details). Additional PKS GCFs that can be correlated to known products or can be associated with product families are those responsible for tropolone (*tro*), azaphilone (*aza*), meroterpenoid (*mt*) and viridicatumtoxin (*vrt*) biosynthesis. A detailed analysis of the respective BGCs can be found in the following sections.

The BiG-SCAPE network analysis of non-PKS BGCs (Fig. 4B) revealed 195 GCFs. Within the NRPS and NRPS-like subset (82 GCF) the two largest clusters are composed of the highly conserved siderophore (*sid*) BGCs. Another subset harbours the terrequinone BGC (*tq* or *tdi*) from *Aspergillus terreus* (Balibar *et al.* 2007) and six homologous BGCs from the *Hypoxylaceae* (for detailed analysis see NRPS-like section). Two clades with each carrying four BGCs can be associated to the biosynthesis of yet unidentified mycosporine-like compounds (*myc*) (Miyamoto *et al.* 2014). Additional NRPS(-like) GCFs with predicted products include a ergopeptine BGC from *A. truncatum* and the rickenyl BGC from *H. rickii*, which are represented by singletons (see respective section for further details).

For terpene pathways, 76 terpene GCFs were found. Known terpene GCFs with more than one BGC are involved in brasilane glycoside (*bra*) (Feng *et al.* 2020) assembly (see brasilane section) and biosynthesis of 1,8-cineole. The latter GCF consists of unclustered monoterpene cyclases that share significant sequence similarity with the 1,8-cineole synthase (*cin*, AHY23922.1) from an endophytic *Hypoxylon* species (Shaw *et al.* 2015). Among the terpene GCF singletons a triterpene BGC from *H. rubiginosum*, belonging to the fernane glycoside family (see triterpene section), is the only BGC that can be further classified.

PKS-NRPS hybrid pathways are subdivided into 21 GCFs with four of them representing cytochalasan BGCs. Within the cytochalasan GCFs the BGC from *H. fragiforme* clustered with the characterised pyrichalasin H pathway from *Pyricularia grisea* (Wang *et al.* 2019a) (see cytochalasan section for an in-depth evaluation). The BiG-SCAPE analysis further revealed a PKS-NRPS GCF composed of the curvupallide BGC from *Curvularia pallescens* (Yokoyama *et al.* 2017) and a BGC from *J. multiformis*. The high structural similarity between both BGCs suggests the production of as yet unidentified curvupallide-type compounds in *J. multiformis*. Related compounds such as the phaeosphaerides and phyllostictines show potent phytoxic activities and have been investigated as potential herbicides indicating that the curvupallide pathway in *J. multiformis* yields products with similar functions (Trenti & Cox 2017, Poluektova *et al.* 2018). The remaining 16 PKS-NRPS GCFs cannot be assigned to a specific product family.

Within the non-PKS BGC dataset 15 GCFs did not fall into any of the categories mentioned before. Six of these GCFs formed clusters, all of which can be further classified. This includes an alkyl citrate GCF (ac), the swainsonine-type NRPS-PKS GCF (*swn*), a RiPP GCF, the nodulisporic acid GCF (*nod*, note that the reference and identified BGC are identical), the sporothriolide GCF (*spo*) and the ergot alkaloid GCF (*ea*). A detailed analysis of these GCFs (except RiPP) can be found in the following sections.

The results of the BiG-SCAPE analysis demonstrate that only very few BGCs are conserved across the different lineages of the *Hypoxylaceae* and that the majority of BGCs are unique (singletons). Furthermore, only few previously characterised BGCs appear in the GCFs preventing product prediction for most of the GCFs. However, our analysis also showed that BiG-SCAPE was in some instances not able to link obvious homologous BGCs. For example, the viridicatumtoxin BGC (*vrt*) from *Penicillium aethiopicum* (MIBiG accession BGC0000168) did not appear in our analysis. This is mainly due to the gene cluster information provided in the MIBiG database, as it also contains several genes outside of the actual BGC, which have a strong weight in global mode settings of BiG-SCAPE. When trimming the viridicatumtoxin BGC from MIBiG to its actual size, it appears as a subnetwork (cutoff value: 0.4) with the *vrt* BGCs from the *Hypoxylaceae* (see Fig. S6). Similarly, the previously characterised ergopeptine BGC from *Claviceps purpurea* was not clustered with the homologous BGC from *A. truncatum* as the additional NRPS copies in the former had a strong impact on the assessment of GCFs. In addition, the similarity of the predicted domains also determines whether two BGCs are clustered together. This prevented the linking of the cytochalasan BGCs from *H. rickii* and *X. hypoxylon*, which share almost the same set of genes, but showed a comparably low protein level similarity. In particular, the similarity threshold is tightly bound to proper gene and open reading frame predictions, which has to be considered as a potential source for errors, especially in eukaryotic systems. Therefore, the network analysis has to be treated with care when estimating the number of new biosynthetic pathways. Nevertheless, BiG-SCAPE reliably recognised closely related BGCs within our *Hypoxylaceae* dataset and thus can serve as a useful approximation of the BGC diversity.

## Biosynthetic diversity of selected pathways in the *Hypoxylaceae*

### Polyketide pathways

#### Azaphilones

Azaphilones are a highly diverse group of fungal PKS-derived pigments, which are characterised by a bicyclic pyranoquinone core (Gao *et al.* 2013). Due to their economical significance as dyes and food additives, in particular in East Asia, azaphilones have become a target for biosynthetic studies and pathway engineering (Chen *et al.* 2017, 2019a). These studies discovered a minimal set of genes necessary for backbone assembly composed of a non-reducing PKS with an SAT-KS-AT-PT-ACP-(*C*-MeT)-R domain structure, an FAD-dependent monooxygenase (FMO) and a ketoreductase (Zabala *et al.* 2012, Chen *et al.* 2017). Later steps in the pathway are catalysed by many different tailoring enzymes, the presence of which greatly varies between azaphilone producing fungal species, and thus leads to extensive diversification of the compound family. One of the key differences between the azaphilone subfamilies is the structure of the attached side chain at the C-8 oxygen. This side chain is either derived from a dedicated cluster-encoded fungal fatty acid synthase (fFAS) (Chen *et al.* 2017), an unclustered fFAS (likely originating from primary metabolism) (Becker *et al.* 2021a), a cluster-encoded highly-reducing PKS (hrPKS) (Zabala *et al.* 2012, Winter *et al.* 2012) or a cluster-encoded non-reducing PKS (nrPKS, often orsellinic acid synthase) (Becker *et al.* 2021a). The fatty acid or polyketide is usually transferred to the bicyclic core by a specialised acyltransferase.

**Fig. 5:**
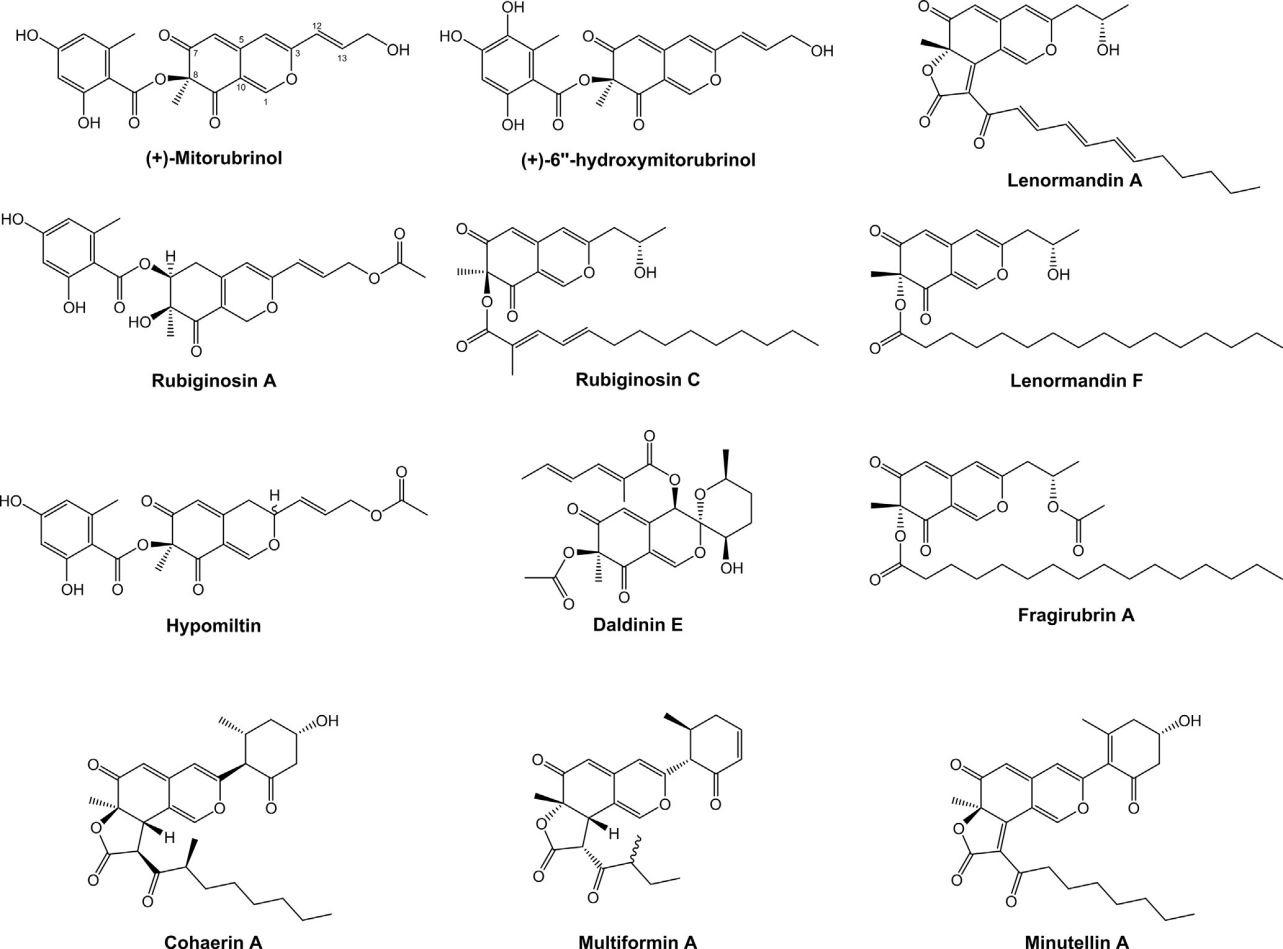
Representative structures of various azaphilone families known from the *Hypoxylaceae*.

Many species within the *Hypoxylaceae*, in particular of the genera *Hypoxylon* and *Jackrogersella*, have been found to contain large quantities of azaphilone mixtures in their stromata (Stadler & Fournier 2006, Helaly *et al.* 2018). In contrast, these pigments were never reported from axenic cultures of the respective producers, indicating that gene expression is mainly coupled to the stromatal ontogeny. So far, more than 60 different azaphilones have been described from the family members that can be roughly divided into subgroups based on the architecture of the side chain (Fig. 5) and the presence of dimeric forms (Helaly *et al.* 2018, Chen *et al.* 2020, Becker *et al*., 2021a). Mitorubrins (Sir *et al.* 2015, Becker *et al.* 2021a), rubiginosins (Quang *et al.* 2004b), hypomiltin (Hellwig *et al.* 2005) and entonaemins A/B (Hashimoto & Asakawa 1998) are characterised by their appended orsellinic acid moieties, while cohaerins (Quang *et al.* 2005a, 2006, Surup *et al.* 2013) and multiformins (Quang *et al.* 2005c) contain short highly reduced methylated polyketide chains at this position. Lenormandins (Kuhnert *et al.* 2015c) and fragirubrins (Surup *et al.* 2018b, Becker *et al*., 2021a) carry long-chain fatty acids while minutellins (Kuhnert *et al.* 2017a) possess shorter unmethylated acyl chains. Daldinins strongly vary from the other azaphilones by having an acetyl moiety at the C-8 oxygen and a further side chain attached to an unusual C-4 oxygen (Hashimoto *et al.* 1994, Quang *et al.* 2004a). Rutilins (Quang *et al.* 2005b, Surup *et al.* 2018b), entonaemin C (Hashimoto & Asakawa 1998) and hybridorubrins (Becker *et al.* 2021a) make up different groups of dimeric azaphilones composed of monomers from the previously mentioned subfamilies (mainly mitorubrins, fragirubrins and lenormandins).

Among the genome sequenced species five are known to produce azaphilones, which includes *H. fragiforme* (mitorubrins, fragirubrins, lenormandins, rutilins, hybridorubrins) (Becker *et al.* 2021a), *H. pulicicidum* (unknown cohaerin-type azaphilones) (Bills *et al.* 2012), *H. rickii* (mitorubrins) (Kuhnert *et al.* 2015a), *H. rubiginosum* (rubiginosins) (Quang *et al.* 2004b) and *J. multiformis* (multiformins) (Quang *et al.* 2005c). There has also been a report about a set of azaphilones isolated from stromata of "*D. concentrica*" (Hashimoto *et al.* 1994). However, at the time of the publication *D. concentrica* comprised a species complex and the respective material under the current definition represented *D. childiae* (see Stadler *et al.* 2014 for an in-depth discussion on this topic). Based on a previous study, where we already correlated two BGCs (*hfaza1*, *hfaza2*) with the production of azaphilones in *H. fragiforme* (Becker *et al.* 2021a), we searched for homologous BGCs in the other species by using the core PKS Hfaza1A as a template. As expected, candidate BGCs were found for all known producers. Potential azaphilone BGCs were also located in the two very closely related species *Hypom. monticulosa* and *Hypom. spongiphila*, but not in their sister taxon *Hypom. submonticulosa*. All other species did not contain obvious azaphilone forming BGCs consistent with the lack of azaphilone pigments in the stromata of these organisms.

##### Group A azaphilones

**Fig. 6:**
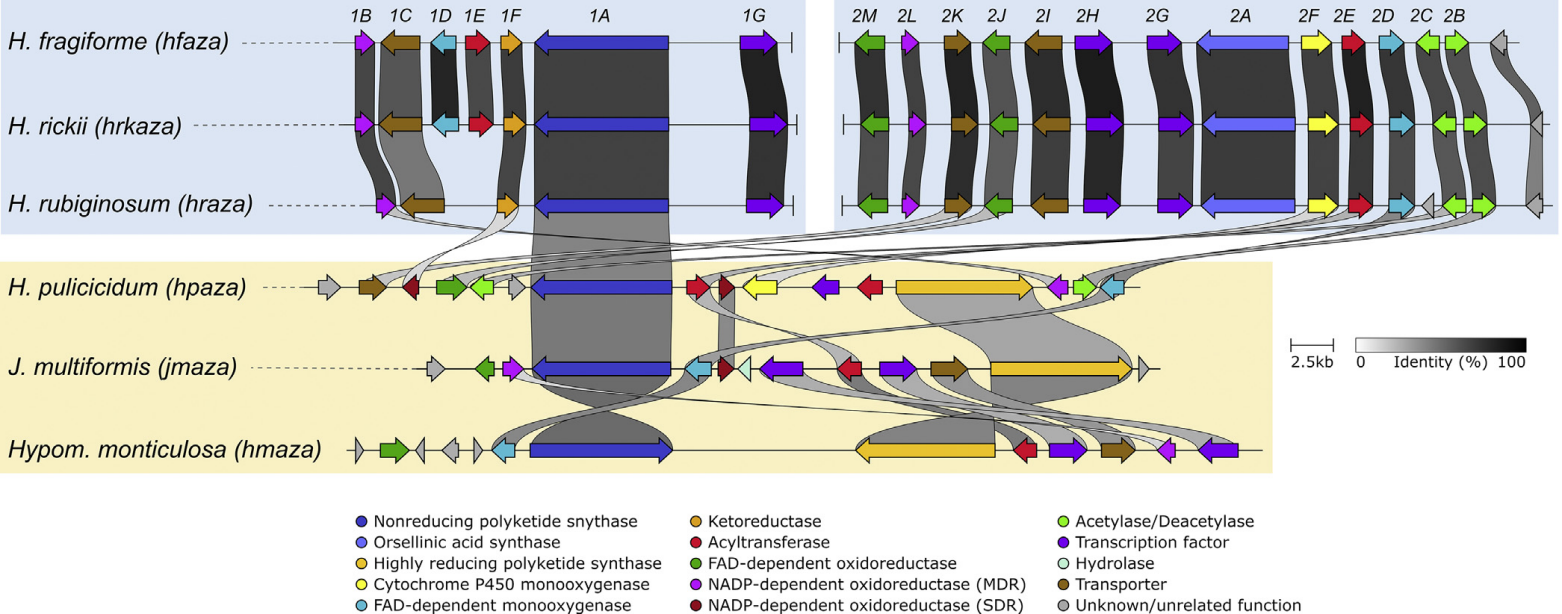
Synteny analysis of azaphilone biosynthetic gene clusters (BGC) identified in different species of the *Hypoxylaceae*. Note that for *hfaza*, *hrkaza* and *hraza* the separate clusters have been drawn in a consecutive order, while in reality they are located on different chromosomes. Group A and B azaphilone biosynthetic gene cluster are highlighted in blue and yellow, respectively. Only the best link (highest protein sequence similarity) for each gene is depicted per BGC pair.

**Fig. 7:**
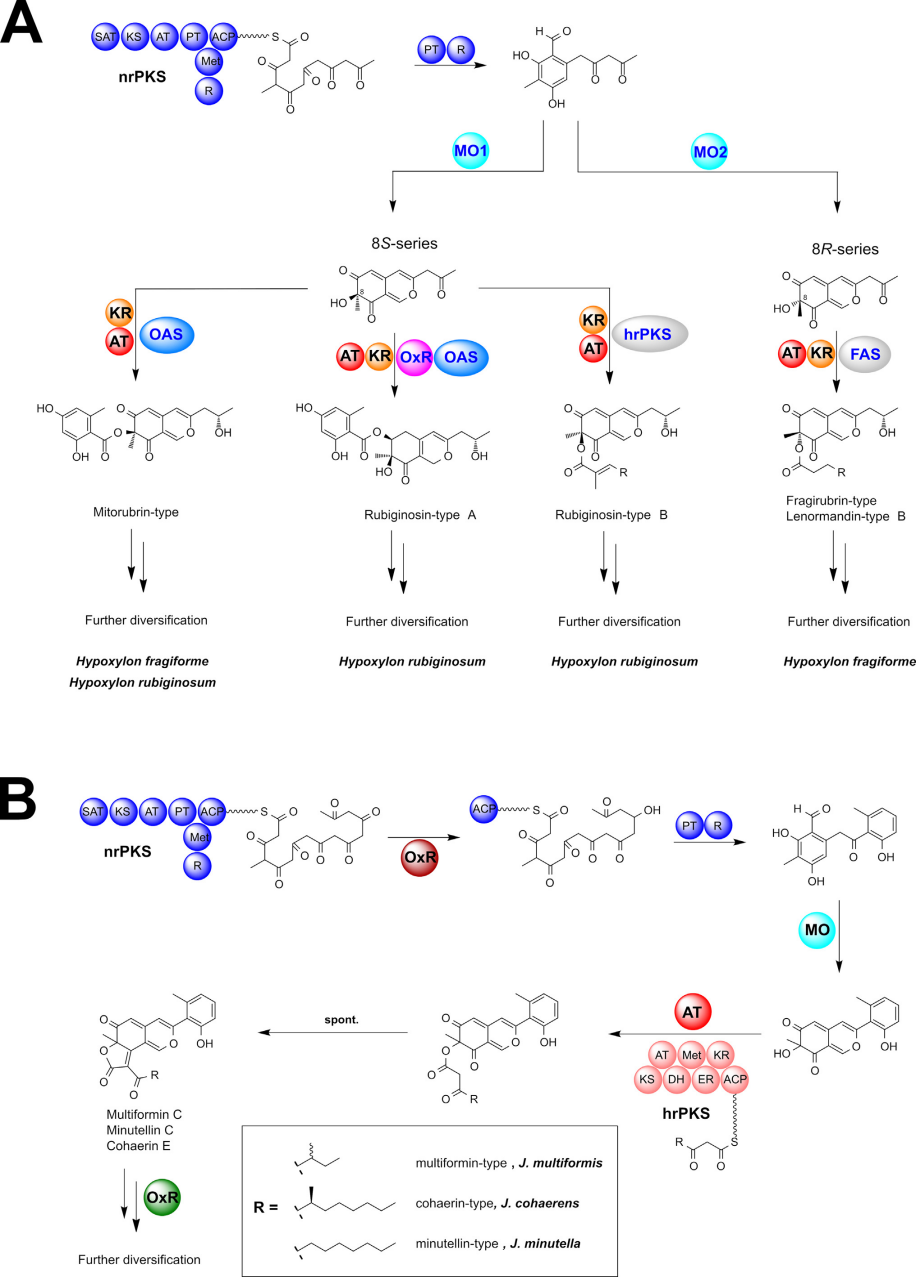
Proposed biosynthetic pathway for azaphilone diversification in the *Hypoxylaceae*. A; group A azaphilone biosynthesis. Enzymes responsible for diversification are highlighted in blue fonts. Lenormandin-type B refers to long-chain lenormandins lacking a third ring. B; cohaerin-type azaphilone biosynthesis. AT: acyltransferase, FAS: fatty acid synthase, hrPKS: highly reducing polyketide synthase, KR: *trans*-ketoreductase, MO: FAD-dependent monooxygenase, nrPKS: non-reducing polyketide synthase, OAS: orsellinic acid synthase, OxR: oxidoreductase.

The identified azaphilone BGCs formed two groups based on the presence of core synthases. Group A azaphilone BGCs consists of two individual BGCs, each of which contain an nrPKS and occur in *H. fragiforme* (*hfaza1/2*), *H. rubiginosum* (*hraza1/2*) and *H. rickii* (*hrkaza1/2*). A synteny analysis shows a high degree of conservation of the respective genes and gene order (Fig. 6). In fact, the clusters of *H. fragiforme* and *H. rickii* are identical in terms of gene manifest, while in *H. rubiginosum* two genes are missing in the first BGC. These missing genes are an FMO and an acyltransferase (close homologs of which are not located elsewhere in the genome). Based on our previous hypothesis about azaphilone biosynthesis in *H. fragiforme* (Becker *et al.* 2021a), we hypothesise that these two genes are responsible for creating a branch-point in the pathway leading to a diverse group of azaphilones with fatty acid side chains (Becker *et al.* 2021a). This theory is further supported by the observation that *H. rubiginosum* stromatal extracts do not contain such type of azaphilones. Despite the presence of a single acyltransferase copy in the azaphilone BGCs of *H. rubiginosum*, this fungus is able to form a variety of azaphilones with different C-8 substitutions. While most azaphilones of this fungus carry an orsellinic acid moiety, rubiginosins C is characterised by a methylated acyl side chain. As this side chain of rubiginosin C also appears as a free acid (termed rubiginosic acid) in the stromatal extracts (Quang *et al.* 2004b), it is likely derived from an unidentified pathway-independent hrPKS. Incorporation of the sidechains could either be facilitated by the acyltransferase located in *hraza2*, which then has a rather broad substrate promiscuity, or another acyltransferase encoded outside of the BGCs (Fig. 7).

We also suggested that two copies of an FMO will establish different stereoisomers at C-8 of the backbone in *H. fragiforme*, which correlates well with the identified groups of azaphilones in this fungus (orsellinic acid containing azaphilones always appear in 8*S*-configuration, while those with fatty acids possess 8*R*-configuration) (Becker *et al.* 2021a). Therefore, a single configuration would be expected for azaphilones produced by *H. rubiginosum*. Initially, this prediction was contradicted by the original reference about the rubiginosins, as rubiginosin A and C apparently vary in their stereochemistry at C-8 (Quang *et al.* 2004b). However, we recently reinvestigated the published data and showed that the stereochemistry at C-8 is indeed *S*-configured across all azaphilones produced by the fungus (Becker *et al.* 2021b). The differences between the *hraza1* BGC and *hfaza1/hrkaza1* BGC can be either explained by a gene loss event in *H. rubiginosum* (or an ancestor) or gain of genes in an ancestor of *H. fragiforme* and *H. rickii*. However, from an evolutionary point of view gene loss is more likely as, in contrast to *hfaza1/hrkaza1*, *hraza1* by itself is not able to establish an azaphilone core structure.

In order to assess whether the FMO genes in the *H. fragiforme* and *H. rickii* azaphilone BGCs are derived from each other by gene duplication or have an independent origin, a phylogenetic tree with characterised fungal FMOs and those identified herein was calculated (Fig. S7). The additional enzyme copies in *H. fragiforme* (Hfaza1D) and *H. rickii* (Hrkaza1D) clustered with insignificant ultrafast bootstrap support together with their homologues from *M. ruber* (MrPigN) (Chen *et al.* 2017) and *A. niger* (AzaH) (Zabala *et al.* 2012), and SorC from the sorbicillinoid pathways but not with CazL from the chaetoviridin BGC (*caz*) of *Chaetomium globosum* (Winter *et al.* 2012). In contrast, the conserved FMOs in the *H. fragiforme*, *H. rickii* and *H. rubiginosum* BGCs (Hfaza2D, Hrkaza2D, Hraza2D) formed a distant subclade, suggesting that Hfaza1D/Hrkaza1D and Hfaza2D/Hrkaza2D do not originate from a recent duplication event. Other recent examples of the convergent recruitment of FMOs in fungi comes from the biosynthesis of the sorbicillinoids in *Penicillium chrysogenum* and *Trichoderma reesei* (Kahlert *et al.* 2020a,b).

As a result of the additional FMO in the cluster, *H. fragiforme* is able to generate a larger diversity of azaphilones as compared to *H. rubiginosum*. Despite the very high similarity between the *H. rubiginosum* azaphilone BGCs and those of *H. fragiforme*, it is remarkable that rubiginosins were not found in *H. fragiforme*, while mitorubrins are present in *H. rubiginosum*. Two possible scenarios could explain this discrepancy. First, each cluster contains an NADP-dependent oxidoreductase gene with no predicted function in the biosynthetic pathway. These genes could encode proteins, which are involved in reductive processes of the backbone, which seemingly only happen during rubiginosin (and entonaemin) biosynthesis. As for the azaphilone BGCs in *H. fragiforme*, the respective homologs might be inactive due to mutations in the promotor region or the coding sequence, preventing further backbone reduction in the mitorubrins. Unfortunately, such questions cannot be easily addressed in the absence of gene expression under laboratory conditions. A second explanation could be a simple interference of the biosynthetic pathway by unknown proteins encoded outside of the cluster.

##### Group B azaphilones

Group B azaphilone BGCs found in *H. pulicicidum* (*hpaza*), *Hypom. monticulosa* (*hmaza*), *Hypom. spongiphila* and *J. multiformis* (*jmaza*) are characterised by a single locus that includes an nrPKS and an hrPKS, an arrangement that is similar to the azanigerone BGC in *Aspergillus niger* (Zabala *et al.* 2012). Compared to the highly conserved clusters of group A, BGCs of group B show more rearrangements and a higher variety of individual genes. The core set of conserved genes within these BGCs encodes two PKSs, an FAD-dependent monooxygenase, an acyltransferase and an FAD-dependent oxidoreductase. Transporter and transcription regulator genes are also present in all clusters, but they show much less sequence similarity. One of the striking features of all group B BGCs is the lack of a ketoreductase gene (*i.e.* homologues to Hfaza1D). This gene encodes a protein, which is essential for azaphilone backbone assembly during the biosynthesis of *Monascus* pigments. Disruption of the respective *Monascus* gene led to the formation of shunt-metabolites without a pyranoquinone ring due to spontaneous aldol cyclisation (Chen *et al.* 2017). In the case of the multiformins a ketoreduction step appears unlikely based on the structural features. All multiformin-type compounds that are known to date (includes cohaerins and minutellins) feature an additional 6-membered ring at the “tail” of the backbone. We assume this is caused through an extended polyketide chain released by the respective nrPKS which spontaneously forms the ring through a Knoevenagel condensation. This structural feature likely prevents aldol cyclisation after chain-release and thus abolishes requirement of a ketoreductase (Fig. 5). An extended nrPKS product has already been observed during the biosynthesis of the azaphilones preasperpyranone and chaetoviridin, which in both cases is the result of an hrPKS derived tetra- or triketide starter unit (Winter *et al.* 2012, Huang *et al.* 2020). Therefore, it is also possible that the multiformin nrPKS uses an alternative starter unit in the form of a partially reduced polyketide (such as 6-methyl salicylic acid) as building block, but a respective PKS is not encoded in the cluster. However, the precise biosynthetic chemistry in this series requires further investigation.

Additional enzymes encoded in the multiformin BGC include different types of oxidoreductases that putatively play a role during the processing of the additional ring. The presence of azaphilones in *H. pulicicidum* stromata has so far only been observed by HPLC-MS analysis and the respective recorded masses did not match any known compound, but pointed towards structures similar to cohaerins (Bills *et al.* 2012). The identified cluster is consistent with this hypothesis, but the presence of further genes encoding a cytochrome P450 monooxygenase, an additional acyltransferase and homologs of the acetylase/deacetylase pair known from the biosynthesis of *Monascus* pigments (Chen *et al.* 2017), indicate that these azaphilones might have different structural features compared to the cohaerins. In contrast, the azaphilone BGC from *Hypom. monticulosa* and *Hypom. spongiphila* is very similar to the multiformin BGC and mainly differs by the lack of an NADP-dependent oxidoreductase (besides the complete rearrangement of the genes). The BiG-SCAPE analysis revealed that *hmaza* and its homolog from *Hypom. spongiphila* are much closer related to the trigazaphilone BGC from *Trichoderma guizhouense* (Pang *et al.* 2020). A synteny analysis with the clinker tool confirmed their similarity and showed that the BGCs contain the same set of genes with protein level similarities for the biosynthetic enzymes between 68 and 82 % (Fig. S8). This implies that the product of *hmaza* is identical or related to trigazaphilones. In *T. guizhouense* the production of these compounds is triggered upon confrontation with *Fusarium oxysporum f. sp. cubense*. The trigazaphilones did not show antifungal activity, but were demonstrated to have an important role in the reduction of oxidative stress in the presence of hydrogen peroxide (Pang *et al.* 2020). *Hypomontagnella* species are potentially also able to induce azaphilone production under oxidative stress, which we will test in the future. The results of Pang *et al.* (2020) also indicate that the biological function of stromatal azaphilones could be related to reduction of oxidative stress. Most of the respective azaphilones have not been tested for related activities so far.

The occurrence of azaphilone BGCs across various lineages of the *Hypoxylaceae* and the observation of these pigments in other non-hypoxyloid taxa of the *Xylariales*, such as *Creosphaeria sassafras* (*Lopadostomataceae*), *Biscogniauxia formosana* (*Graphostromataceae*) and *Microdochium bolleyi* (*Microdochiaceae*) (Helaly *et al.* 2018) implies that these pathways are ancient and already existed in ancestral lineages of the order. Various azaphilone producers in the *Hypoxylaceae* have not been sequenced so far and we expect to find a much larger diversity of azaphilones BGCs based on the structural features of their products (*e.g.* daldinins E/F, lenormandin A, cohaerins) once the respective genomes become available (research is currently ongoing). With the discovery of azaphilone BGCs in the *Hypomontagnella* species it also became clear that formation of azaphilones is not always associated with stromatal development in the *Hypoxylaceae*. Therefore, it appears likely that additional BGCs in other unsequenced family members that are devoid of stromatal azaphilones will be found, further extending the diversity of azaphilone pathways.

#### DHN-derived polyketides

Besides azaphilones, binaphthyl and benzo[*j*]fluoranthene derivatives are the most prevalent pigments accumulated in the stromata of *Hypoxylaceae* (Stadler & Fournier 2006). In particular the simplest congener 1,1'-binaphthalene-4,4',5,5'-tetrol (BNT) is common across various family lineages and can be found as either sole detectable secondary metabolite of stromatal extracts as observed for some *Annulohypoxylon* species like *A. purpureonitens* and *A. violaceopigmentum* where it is responsible for the intense violet KOH reaction, or BNT is accompanied by chemical related and/or unrelated compounds as reported for various *Daldinia, Hypoxylon* and *Jackrogersella* species (Stadler *et al.* 2014, Kuhnert *et al.* 2017b). Those related compounds are particularly important as chemotaxonomic markers to distinguish between closely related species, including daldinone A, truncatone A–D, hypoxylonols, urceolone and hinnulin A (Kuhnert *et al.* 2017b). While these pigments mainly occur in the stromata of the *Hypoxylaceae*, they were sporadically also reported from cultures such as hinnulin A–D from *Nodulisporium hinnuleum* (now *Hypoxylon hinnuleum*) and daldinones B, C, H and J, as well as hypoxylonol C from an endophytic *Annulohypoxylon* sp. (Schlingmann *et al.* 2011, Liu *et al.* 2017a). All aforementioned examples are dimeric naphthalene derivatives, but monomeric forms such as 8-methoxy-1-naphthol which is produced by most *Daldinia* species in cultures are frequently found as well (Bitzer *et al.* 2008). Additionally, a mantis gut-associated *D. eschscholtzii* strain was able to form a range of immunosuppressant enantiomeric trimers, named (±)-dalesconols A–C and (±)-daeschol A (Zhang *et al.* 2008).

All these structures likely share a common biosynthetic origin derived from the dihydroxynaphthalene (DHN)-melanin pathway (Stadler & Fournier 2006). This correlation in the *Hypoxylaceae* was so far only proven for the dalesconols, where a PKS (designated PKSTL) in the producer organism *D. eschscholtzii* with high structural similarity to other known tetrahydroxynaphthalene (T_4_HN) synthases (T_4_HN is the precursor of DHN) was knocked out abolishing the production of dalesconols and leading to formation of albino mutants. The latter observation also proved that the dark, often greenish pigmentation of mycelia in many *Daldinia* species requires the activity of the PKS enzyme. Tailoring genes involved in the processing of T_4_HN to DHN namely the T_4_HN reductase, scytalone dehydratase and T_3_HN reductase were not found in the proximity of the PKS, which is a common observation in fungi where these genes are usually highly conserved but also frequently not clustered. Addition of tricyclazole to the fermentation broth, an inhibitor of T_4_HN and T_3_HN reductases, significantly reduced production of dalesconol A and B demonstrating the importance of these proteins for the biosynthesis of these compounds. A laccase gene located directly next to the PKS in the same organism was proven to be involved in radical coupling of DHN and its precursors probably *via* the intermediate BNT establishing the polycyclic structure of dalesconols and daeschols (Fang *et al.* 2012).

**Fig. 8:**
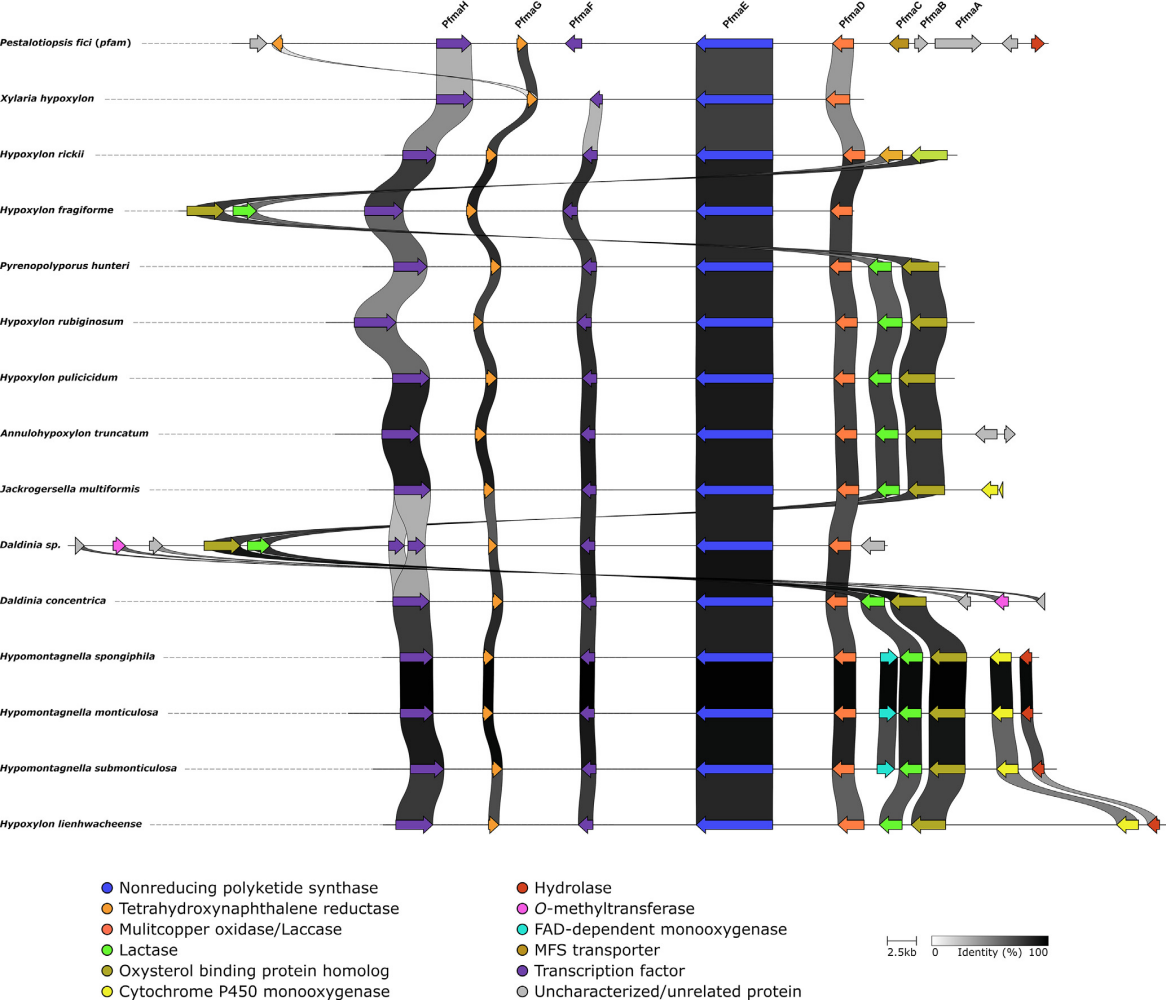
Comparison of the characterised biosynthetic gene cluster involved in the biosynthesis of dihydroxynaphthalene (DHN) from *Pestalotiopsis fici* and those identified in *Xylaria hypoxylon* and various *Hypoxylaceae* species.

Some of the genome-sequenced species in this study are known producers of BNT including *D. concentrica*, *H. pulicicidum*, *J. multiformis* and *P.* *hunteri* (Stadler & Hellwig 2005, Bills *et al.* 2012). In addition, *A. truncatum* contains truncatone A in its stromata (Kuhnert *et al.* 2017b) suggesting that a DHN pathway is located in the genomes. Screening of all *Hypoxylaceae* genomes and the *X. hypoxylon* genome based on the PKSTL (tetrahydroxynaphthalene synthase from *D. eschscholtzii*) protein sequence similarity revealed the presence of homologs with 80.0–93.0 % protein similarity in all species. Analysis of the genetic loci showed that the PKS is part of a BGC with highly conserved architecture. Additional genes encode a T_4_HN reductase, a laccase (multicopper oxidase) and two transcription factors, homologs of which were already reported from a DHN cluster (*pfma*) in the distantly related fungus *Pestalotiopsis fici*. Gene knock-out studies in the latter demonstrated that the BGC is part of the DHN pathway and is also important for conidia pigmentation and development (Zhang *et al.* 2017). A synteny analysis between the *pfma* BGC, *X. hypoxylon* BGC and *Hypoxylaceae* BGCs confirmed their high similarity (Fig. 8) indicating that they are likely responsible for DHN production and derivatives thereof. Other enzymes involved in DHN biosynthesis, *i.e.* scytalone dehydratase and T_3_HN reductase, were found encoded in the genomes of all investigated strains, but similar to most other fungal genomes located separately on different contigs.

**Fig. 9:**
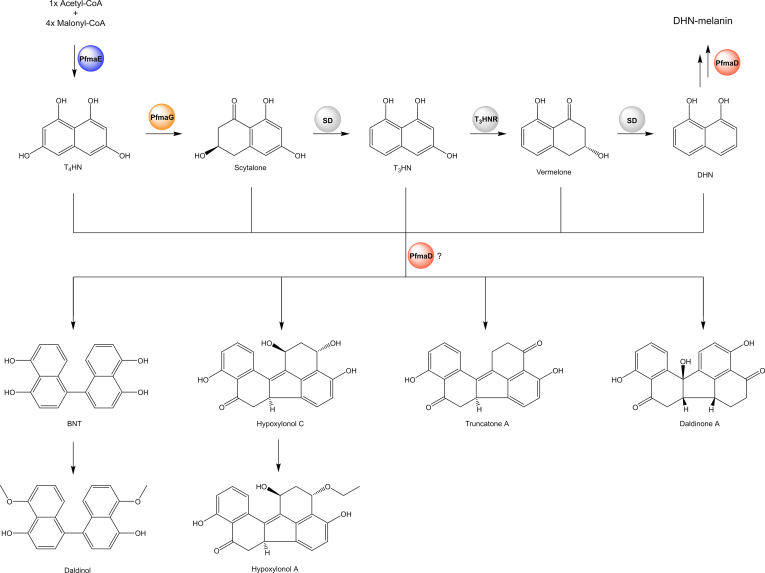
Biosynthetic scheme for the formation of dihydroxynaphthalenes (DHN) and related dimeric structures in the *Hypoxylaceae*. Binaphthyl and benzo[*j*]fluoranthenes diversity is probably determined by the selectivity of the laccase PfamD, which supposedly can accept DHN but also various pathway intermediates for dimerisation. AT: acyltransferase, MT: methyltransferas, PfmaE: nrPKS, PfamG: tetrahydroxynaphthalene (T_4_HN) reductase, SD: scytalone dehydratase, T_3_HNR: trihydroxynaphthalene (T_3_HN) reductase.

Based on the structure of the different families of binaphthyl and benzo[*j*]fluoranthenes observed in the stromata of the *Hypoxylaceae*, it is likely that DHN as well as its intermediates such as T_3_HN and vermelone but also pathway shunts can serve as substrate for dimerisation. The mechanism for compound dimerisation has been described for various fungal natural products and usually involves the action of either laccases or P450 monooxygenases (Hüttel & Müller 2021). While a single C-C bond formation in the case of binaphthyls such as BNT can be likely attributed to the activity of the PfmaD (laccase) homolog in the BGCs, it is more difficult to predict the responsible enzyme for dimerisation during benzo[*j*]fluoranthene biosynthesis where two C-C bonds are established. It is possible that the laccase is also able to catalyse the formation of both bonds in a consecutive fashion or that other enzymes are involved (Fig. 9). Interestingly, all *Hypoxylaceae* DHN BGCs are extended by a lactase and an oxysterol binding protein encoding genes next to either the laccase or the second transcription factor. Homologs of both enzymes were also located in the *X. hypoxylon* and *P. fici* genomes but distantly located from the *pfma* BGC. In some *Hypoxylaceae* genomes other putative tailoring genes encoding for either a P450 monooxygenase, FAD-dependent monooxygenase, *O*-methyltransferase or hydrolase were encoded in the proximity of the BGCs. None of these enzymes can be directly correlated with structural features of known naphthalene products from the producer, except for the *O*-methylation in 8-methoxy-1-naphthol, a chemotaxonomic marker for *Daldinia* species. The presence of an *O*-methyltransferase in the *D. concentrica* and *Daldinia* sp. BGC could be related to the product formation. This observation is consistent with the usual absence of 8-methoxy-1-naphthol in cultures of non-daldinoid species.

The high conservation of the DHN pathway between all analysed members of the *Hypoxylaceae* and also to *X. hypoxylon* strongly indicates the importance of the BGC. Melanin is a crucial structural component of the cell wall or associated structures of various cell types where it likely functions as protective layer against UV light, temperature stress, free radicals and desiccation. Additionally, its production has been also linked to fungal virulence in various phytopathogens where melanin for example accumulates in the appressoria of *Pyricularia* *oryzae* to sustain turgor pressure (Ryder & Talbot 2015, Cordero & Casadevall 2017). Disruption of related pathways in other fungi have shown that melanin is often accumulated in reproductive structures such as conidia (Al-Laaeiby *et al.* 2016, Zhang *et al.* 2017) and therefore it can be assumed that the typical light brown to dark brown pigmentation of the ascospores in the *Hypoxylaceae* is a result of the activity of the DHN BGC. Melanisation is putatively also involved during the maturation of stromata, which are black in species such as *A. truncatum*, *D. concentrica*, *Hypom. monticulosa* or *J. multiformis* or turn black in overmature specimens once the pruina or surface pigments disappears, thus providing an effective protection of the spores from various abiotic stresses. Formation of dinaphthalenes during ontogeny of stromata is likely related to melanisation as they share the same BGC and thus the dimers could also represent shunt metabolites. This would also explain why these compounds are infrequently detected in culture. Generally, BNT and most benzo[*j*]fluoranthenes produced by the *Hypoxylaceae* have been found to be devoid of specific antimicrobial activity, but some compounds like the truncatones and viridistratins showed moderate cytotoxicity (Sudarman *et al.* 2016, Becker *et al.* 2020). In addition, BNT and hypoxylonol C were demonstrated to protect pancreatic β-cells against apoptotic damage probably through reduction of oxidative stress indicating that these compounds might reduce levels of reactive oxygen species (Lee *et al.* 2019). Therefore, binaphthyls and benzo[*j*]fluoranthenes might provide an additional layer of protection for perithecia against certain stress conditions and predators. This is also consistent with the fact that these pigments are stored in granules which accumulate around the perithecia (Stadler & Fournier 2006).

#### Tropolones

Tropolones are compounds produced by plants, fungi and bacteria that feature a seven-membered non-benzenoid aromatic ring system that often show potent bioactivity. Fungal tropolones are relatively rare with only around 30 reported structures to date including stipitatic acid, sepedonin, malettinins and tropolone-derived xenovulene A (Bentley 2008, Guo *et al.* 2019, Schotte *et al.* 2020). In fungi, tropolones are formed by the initial action of a nrPKS which establishes a polyketide aldehyde with a methylated six-membered ring. Subsequent oxidative ring expansion by the activity of an FAD-dependent monooxygenase and a 2-oxoglutarate-dependent dioxygenase leads to the typical core structure (Davison *et al.* 2012, Schor *et al.* 2018).

**Fig. 10:**
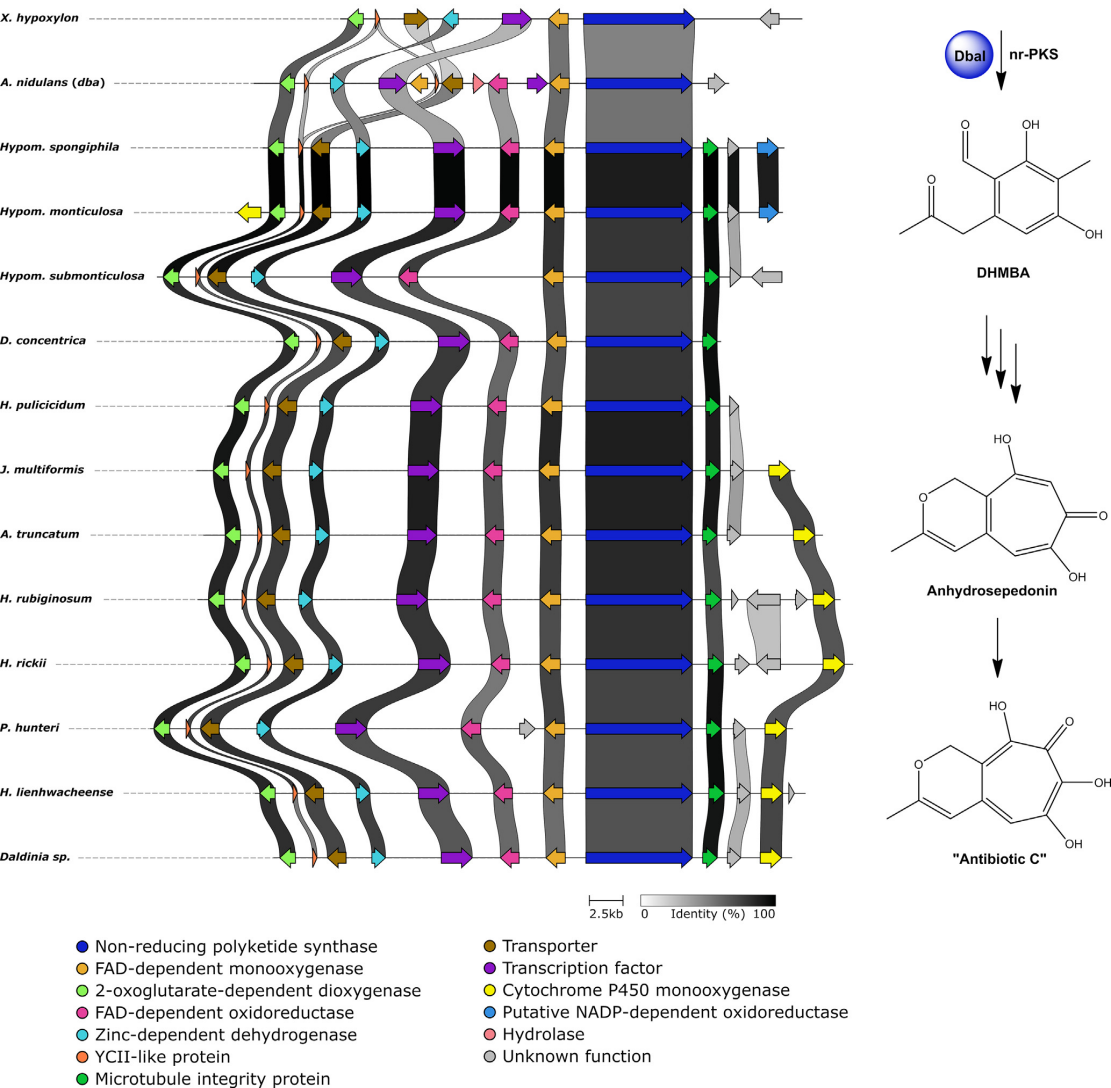
Homology analysis of *dba* biosynthetic gene cluster (BGC) from *Aspergillus nidulans* with related tropolone BGCs from various *Hypoxylaceae* species and *Xylaria hypoxylon*. Biosynthetic scheme for the predicted sepedonin-type pathway products is shown on the right.

So far no tropolone has been reported from the *Hypoxylaceae* in the literature, but we isolated anhydrosepedonin and a previously patented analogue termed antibiotic C (McDonald *et al.* 1983) from the culture broth of *Hypoxylon rickii* (unpublished data; did not appear in the present screening). Related novel structures have also been identified and isolated from *H. lienhwacheense* (currently work in progress). Furthermore, an undetermined *Nemania* species from the related family *Xylariaceae* has been found to produce a series of tropolones named nemanolones (Kornsakulkarn *et al.* 2017). Based on the knowledge about fungal tropolone biosynthesis we searched for BGCs containing an nrPKS, FAD-dependent monooxygenase and 2-oxoglutarate-dependent dioxygenase. Surprisingly, each species except *H. fragiforme* contains a single BGC with all the required genes. A homology analysis revealed a very high similarity between all *Hypoxylaceae*-derived clusters with conserved gene content and order (Fig. 10). In addition to the required core genes, these BGCs include a zinc-dependent dehydrogenase, an FAD-dependent oxidoreductase, a transcription factor, a transporter and an YCII-like protein. In all *Hypoxylaceae* tropolone BGCs, the nrPKS is also accompanied by a homolog of the essential microtobule integrity protein mal3 (Beinhauer *et al.* 1997), which however, is the only copy of this gene in the respective genomes, implying that the position of the BGCs is highly conserved. The majority of clusters also have a P450 monooxygenase in their proximity that could potentially be associated with product formation. *Xylaria hypoxylon* also possesses a highly similar cluster that is slightly rearranged and lacks the FAD-dependent oxidoreductase.

Further analysis with the cblaster tool indicated the presence of a related BGC in *A. nidulans* (*dba*) involved in the production of 2,4-dihydroxy-3-methyl-6-(2-oxopropyl)benzaldehyde (DHMBA) (Gerke *et al.* 2012). Comparison of the *dba* BGC and those from the *Hypoxylaceae* confirmed their similarity with the *dba* BGC being expanded by an additional FAD-dependent monooxygenase gene and a hydrolase gene (Fig. 10). Interestingly, despite most of the genes in the *dba* BGC being expressed only DHMBA, the product of the nrPKS *dbaI* was found (Gerke *et al.* 2012). DHMBA has already been predicted to be the precursor for sepedonin formation (Davison *et al.* 2012), therefore it is very likely that the identified tropolone BGC in the *Hypoxylaceae* and *dba* in *A. nidulans* can form sepedonin-related compounds. We are currently investigating the tropolone biosynthetic pathway in the *Hypoxylaceae* to obtain further insights into the biosynthesis of sepedonin and related compounds.

The high conservation of the putative tropolone BGC across all *Hypoxylaceae* lineages points towards a high ecological significance of the pathway product for the species. Sepedonin, anhydrosepedonin and antibiotic C show significant activity against a range of microorganisms, including Gram-positive and Gram-negative bacteria as well as various yeasts and filamentous fungi (McDonald *et al.* 1983, Nagao *et al.* 2006). Antibiotic C has even been successfully tested in mouse models with *Candida albicans* infection (McDonald *et al.* 1983). This data suggests that the compounds may be used under competitive conditions to defend against other microorganisms. The broad spectrum activity of sepedonin and congeners, in particular against fungi, also implies that a resistance mechanism has been evolved in the producer species. As the molecular target of these compounds is unknown further studies are necessary to understand the biology and chemistry of sepedonins.

### Amino acid derived pathways

#### Ergot alkaloids

Ergot alkaloids are a class of fungal natural products characterised by a tryptophan-derived tetracyclic ergoline core (Jakubczyk *et al.* 2014). Originally identified from the ergot fungus *Claviceps purpurea*, these compounds are now known from a broad range of fungal species including *Epichloë* spp., *Balansia obtecta*, *Aspergillus japonicus*, *Penicillium commune*, and *Periglandula* spp. associated with morning glories (Florea *et al.* 2017, Steiner & Leistner 2018). Ergot alkaloids became infamous in particular due to their detrimental effects on humans and livestock as they interact with serotonin, dopamine and adrenergic receptors. The toxins are usually ingested by consumption of ergot infested grain or symbiotically colonised grass (mainly with members of the genus *Epichloë*). Various pharmaceutical applications for ergot alkaloids and their semi-synthetic derivatives have been developed including treatment of migraine and Parkinson's disease (Florea *et al.* 2017). The economic importance of these compounds led to the first biosynthetic investigations more than 60 years ago (Gröcer & Floss 1998). Consequently, today the biosynthetic pathway for most ergot alkaloids is well understood and the underlying BGCs have been fully characterised.

**Fig. 11:**
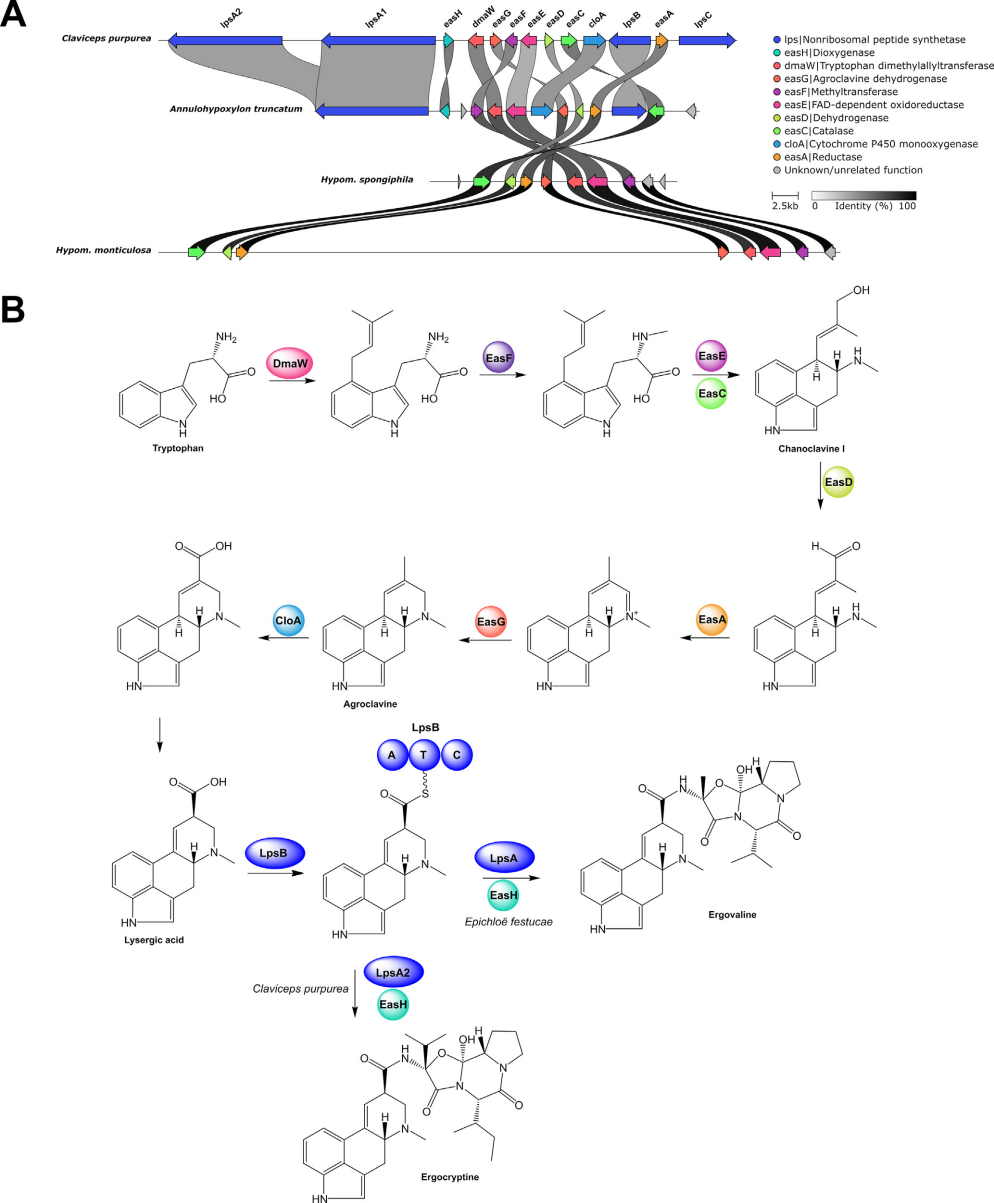
A; Synteny analysis between the ergopeptine biosynthetic gene cluster (BGC) from *Claviceps purpurea* and related cluster identified from *Annulohypoxylon truncatum*, *Hypomontagnella spongiphila* and *Hypom. monticulosa*. B; biosynthetic pathway for the production of ergopeptines in *Epichloë festucae* and *C. purpurea*. The set of identified genes in *Hypom. spongiphila* would allow for the production of agroclavine, while *A. truncatum* possess the necessary genes for the production of an unidentified ergopeptine.

Assembly of the ergot alkaloid backbone starts with prenylation of *L*-tryptophan, and a series of oxidoreductive processes establishes the ergoline scaffold. Depending on the producer, the backbone can be further modified to yield more complex pathway products such as fumigaclavines or ergopeptines, the latter of which involves the activity of up to four additional NRPS enzymes (Fig. 11) (Gerhards *et al.* 2014).

No ergot alkaloid has been reported to date from the *Hypoxylaceae*, but they have been isolated from two members of the sister family *Xylariaceae* (*Xylaria nigripes*, *Dicyma* sp.) (Vázquez *et al.* 2003, Hu & Li 2017). Despite this fact, *A. truncatum* contains a BGC which shows striking similarities with the ergopeptine cluster from *C. purpurea*. Both BGCs contain almost the same set of genes, with the *A. truncatum* BGC being reduced by two NRPS genes, one of which consists of a single module and the other of three modules (Fig. 11). The missing genes are responsible for ergopeptine diversification in *C. purpurea*, but are not essential for ergopeptine biosynthesis (Haarmann *et al.* 2008). Literature searches for related BGCs revealed that the ergovaline producer *Epichloë festucae* encodes the identical set of genes as *A. truncatum*, but was not included in the homology analysis due to incompleteness (Florea *et al.* 2017). Therefore, it seems likely that *A. truncatum* is able to produce ergovaline or congeners under the correct conditions.

Mass searches in LCMS data obtained from the culture-derived crude extracts of *A. truncatum* suggested the presence of ergovaline under our culture conditions (Fig. S9). Based on this initial finding, we screened the other *Hypoxylaceae* and *X. hypoxylon* genomes for the presence of ergot alkaloid BGCs. Only *Hypom. monticulosa* and *Hypom. spongiphila* revealed a BGC which includes the core set of genes necessary for ergot alkaloid backbone assembly. The BGC is highly conserved between the two species, but is interrupted in *Hypom. monticulosa* by a 45 kbp long stretch of non-coding DNA. The *Hypomontagnella* BGCs contain fewer genes compared to the *A. truncatum* ergopeptine BGC and do not encode NRPS proteins (Fig. 11). Additionally, no homologs of the ergopeptine NRPSs were found in other genomic loci. Based on the present set of genes and the similarity of the encoded enzymes to other known ergot alkaloid biosynthesis proteins (52–72 %, Table S4), it can be proposed that agroclavine or festuclavine are potential pathway products in both *Hypomontagnella* strains. Our screening efforts did not result in the identification of these metabolites in the respective crude extracts (from cultured material). The results of a previous study also showed that ergopeptine BGCs with eliminated late-step genes in *Epichloë coenophiala* mainly accumulated early pathway intermediates, such as chanoclavine and ergotryptamine (Florea *et al.* 2016), but none of these compounds could be detected in our crude extracts. In addition, transcriptomic data of *Hypom. monticulosa* and *Hypom. spongiphila*, which were generated in the course of a previous study (Tian *et al.* 2020), did not reveal any expression of the genes and thus the activity of the cluster cannot yet be confirmed.

Cblaster searches using the *A. truncatum* ergot alkaloid biosynthesis proteins were conducted to assess whether previously published related genomes also contain ergot alkaloid BGCs. Surprisingly, two complete ergopeptine BGCs were also found in the genomes of *Hypoxylon* sp. *EC38* (phylogenetically related to *H. pulicicidum*) and *Daldinia* sp. *EC12* (Shaw *et al.* 2015) (Fig. S10). This finding indicates that ergot alkaloid BGCs occur more commonly in the *Hypoxylaceae* than our initial analysis revealed, and that these BGCs are present in most of the family lineages. Furthermore, the high similarity of the encoded enzymes with those from *C. purpurea*, *E. festucae* and *Aspergillus fumigatus* (Table S4) suggests that the genes have remained highly conserved across the *Ascomycota* during evolution. A tblastx analysis between the *A. truncatum* and *Hypom. spongiphila/monticulosa* ergot alkaloid BGC revealed the remainders of an NRPS gene in the latter (Fig. S11). Similar observations have been made for the ergot alkaloid BGC in *Claviceps fusiformis*, where truncated non-functional homologs of the *lpsB* NRPS gene have been found (Lorenz *et al.* 2007). The authors concluded that late pathway steps for ergopeptine biosynthesis were lost during evolution, a scenario that likely also occurred in the *Hypomontagnella* lineage.

#### Compounds derived from NRPS-like enzymes

Fungal terphenyls are a widespread class of natural products with a diverse range of activities which have been isolated from basidiomycetes and ascomycetes alike (Liu 2006). They are biosynthetically derived from *L*-tyrosine or *L*-phenylalanine through the condensation of two units of identical deaminated amino acids (α-keto acids) catalysed by NRPS-like enzymes resulting in a quinolone core structure. These enzymes are characterised by their highly conserved A-T-TE domain structure, but the TE (thiolesterase) domain allows for the formation of either a furanone, quinone or even dioxolanone ring depending on its catalytic triad (Van Dijk *et al.* 2016, Geib *et al.* 2019). NRPS-like enzymes have not only been demonstrated to be responsible for terphenyl and related furanone production, but also to be involved in asterriquinone biosynthesis. Asterriquinones are bisindolylbenzoquinones that originate from *L*-tryptophan (Balibar *et al.* 2007). Associated tailoring enzymes found in the terrequinone A cluster have been shown to reduce the quinone core and catalyse prenylation (Balibar *et al.* 2007). Despite the ubiquitous presence of NRPS-like BGCs in fungal genomes, these pathways have rarely been investigated beyond the function of the core enzymes.

**Fig. 12:**
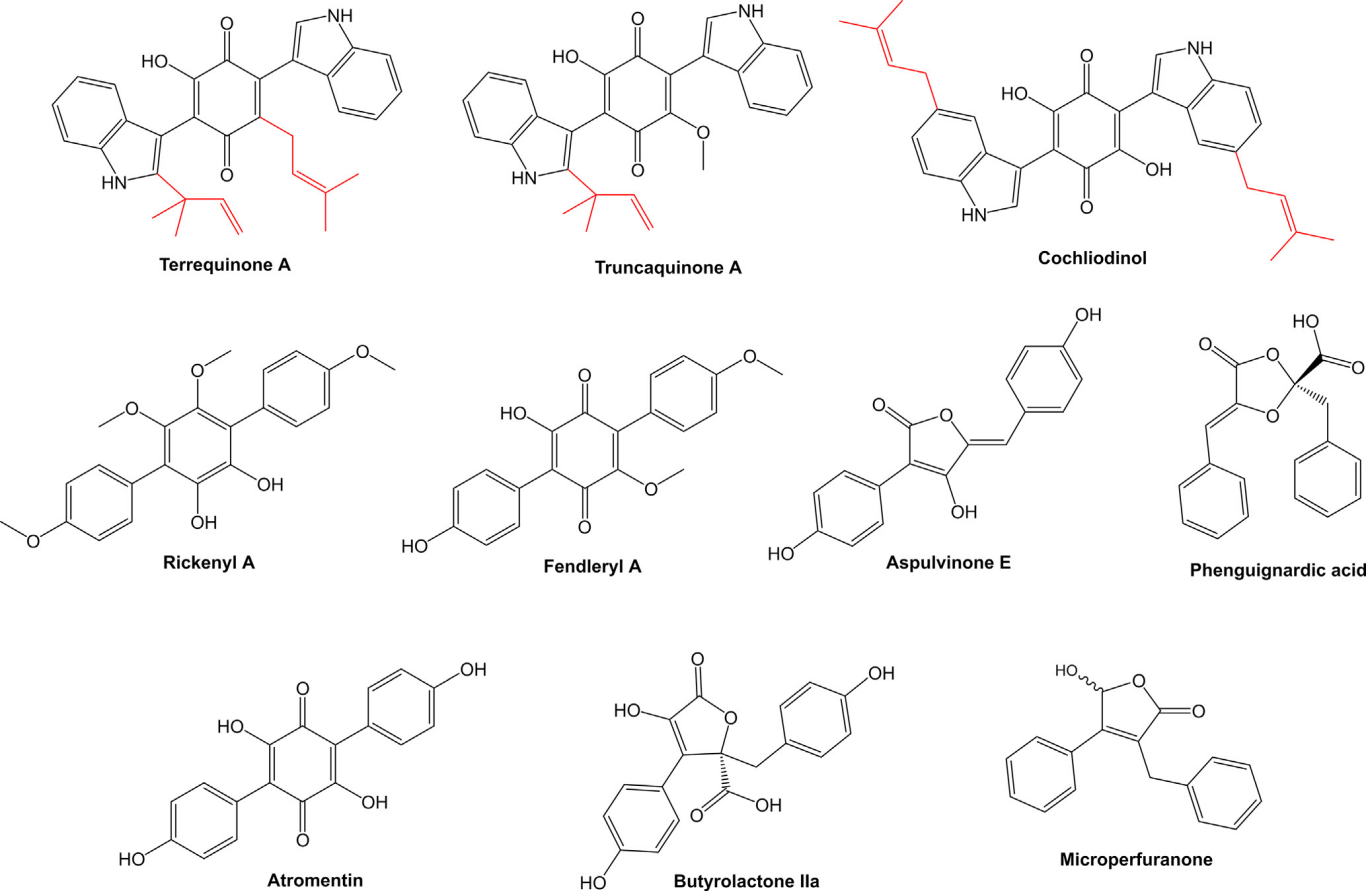
Representative structures of fungal compounds derived from NRPS-like enzymes with A-T-TE domain organisation. Prenylation patterns in the asterriquinone-type compounds are highlighted in red.

**Fig. 13:**
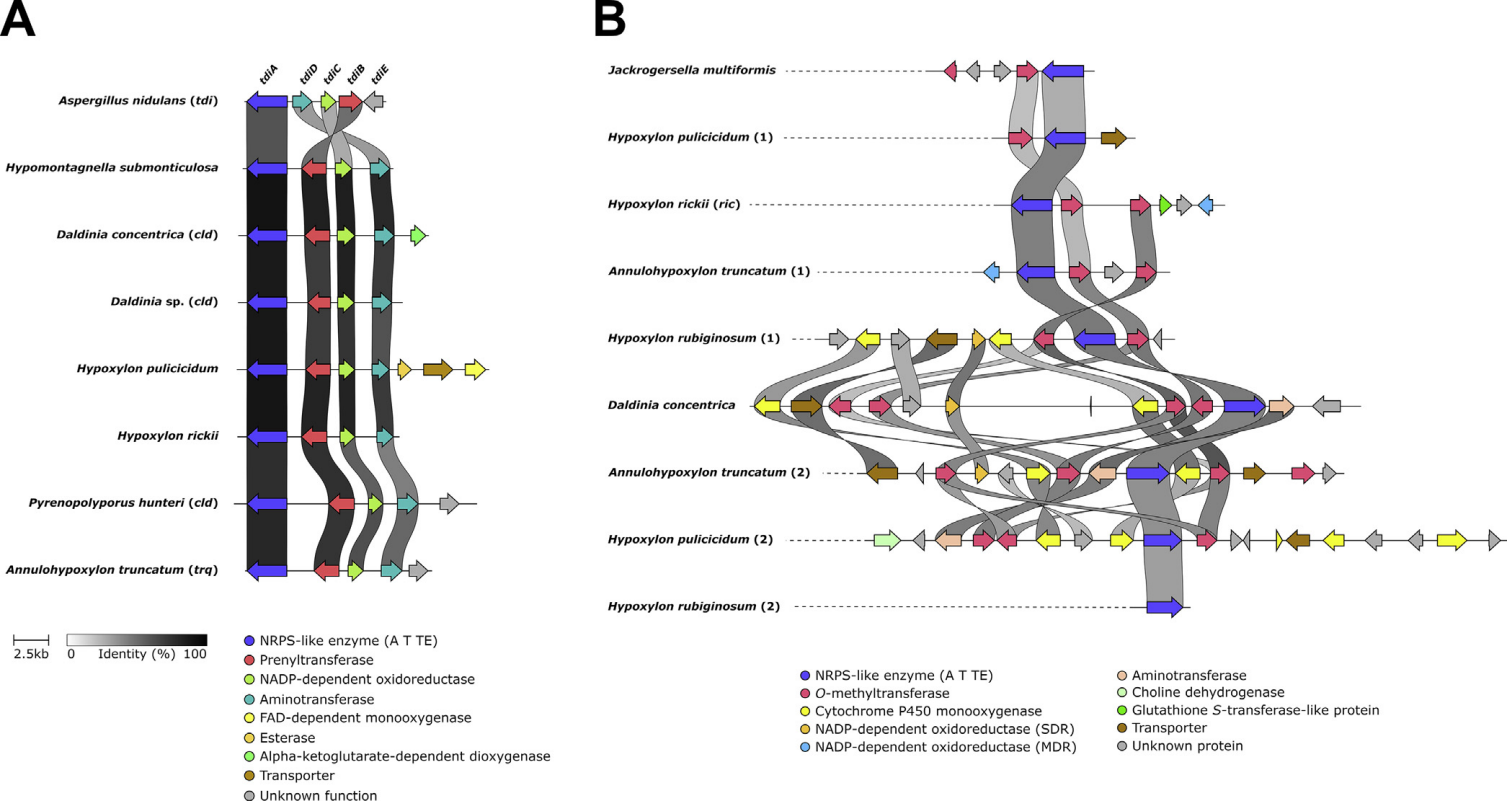
Diversity and comparison of biosynthetic gene cluster (BGC) with NRPS-like core enzymes with an A-T-TE domain structure in the *Hypoxylaceae*. Protein level similarity is visualised with the clinker tool. Numbers in brackets refer to copy numbers of NRPS-like enzymes in the phylogenetic tree of Fig. 14. A; terrequinone-type BGCs. B; terphenyl-type or other non-terrequinone-type NRPS-like BGCs.

**Fig. 15:**
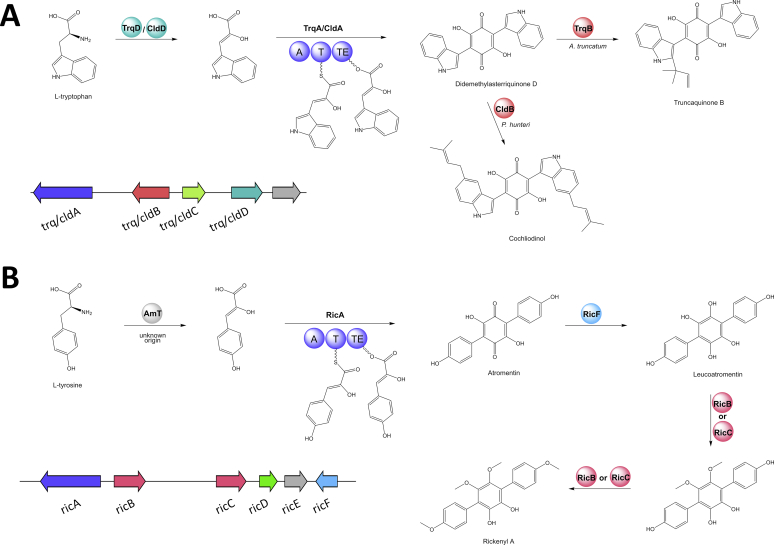
Proposed biosynthetic pathways for truncaquinones/cochliodinol (A) and rickenyls (B). Representative gene clusters from *Annulohypoxylon truncatum*/*Pyrenopolyporus hunteri* (A) and *Hypoxylon rickii* (B) are shown. AmT: aminotransferase, GST: Glutathione S-transferase-like, MT: *O*-methyltransferase, OxR: oxidoreductase, PT: prenyltransferase.

Within the *Hypoxylaceae* various related pathway products have already been identified. The rickenyls and fendleryls are known terphenyl derivatives isolated from cultures of *H. rickii* and *H. fendleri* (identity of the latter species doubtful, *cf.* Becker and Stadler 2021), respectively, while the truncaquinones obtained from *A. truncatum* stromata and cochliodinol reported from cultures of the *Pyrenopolyporus* lineage belong to the asterriquinone family (Fig. 12) (Bitzer *et al.* 2008, Kuhnert *et al.* 2015b, Surup *et al.* 2016, Intaraudom *et al.* 2017). Screening for TE domain-containing NRPS-like genes revealed 16 BGCs scattered throughout the *Hypoxylaceae* genomes. Seven of these were highly similar with a conserved core organisation of genes that encoded for the NRPS-like enzyme, an aminotransferase, a NADP-dependent reductase and a prenyltransferase (PT). A synteny analysis with the known terrequinone A cluster (*tdi*) from *Aspergillus nidulans* showed a significant similarity between the clusters suggesting that they assemble related compounds (Fig. 13A). The presence of a single copy of a terrequinone-type cluster in *A. truncatum* (*trq*) and *Pyrenopolyporus hunteri* (*cld*), the producers of the terrequinone congeners truncaquinone and cochliodinol, respectively, allows the correlation of these BGCs with the latter compounds. While the biosynthesis of cochliodinol in *P. hunteri* can be explained by the present set of genes in the *cld* BGC, an *O*-methyltransferase required for truncaquinone A biosynthesis in *A. truncatum* is missing in the *trq* BGC and its proximity. As *A. truncatum* possesses an additional BGC with NRPS-like enzymes (see next paragraph for additional details) that include *O*-methyltransferase genes, it is possible that cross-interaction between the pathways is responsible for the methylation of truncaquinone A or that the *O*-methyltransferase gene is located on a completely different locus. In contrast to the *tdi* cluster, the conserved NADP-dependent reductase does not appear to have an obvious function for the assembly of truncaquinone and cochliodinol (Fig. 15) as there is neither a tetrahydroxybenzol ring nor a prenylated quinone structure present in the final molecules. However, the enzyme could still be involved in the formation of unidentified shunt products, which however requires further investigation.

In addition to the mentioned known producers, five additional genomes carry a terrequinone-type cluster including *H. pulicicidum*, *H. rickii*, *D. concentrica*, *Daldinia* sp. and *Hypom. submonticulosa*. HPLC-MS guided screening of respective culture extracts showed that *D. concentrica* and *Daldinia* sp. are able to produce cochliodinol (Fig. S12). As the diversity of asterriquinones produced by the *Hypoxylaceae* is mainly determined by the prenylation pattern of the compounds, the respective BGCs offer a basis for engineering of prenyltransferases.

Apart from the asterriquinone-type BGCs, the production of rickenyls and fendleryls suggests the presence of other BGCs with NRPS-like core enzymes in the *Hypoxylaceae*. Genome mining of *H. rickii* quickly revealed the presence of such a cluster (*ric*), which also encodes two *O*-methyltransferases (RicB, RicC) and an NADP-dependent oxidoreductase (RicF) (Fig. 13, Fig. 15). We predict that the assembly of rickenyls is mediated via the atromentin pathway, which is well-characterised for various basidiomycetes (Schneider *et al.* 2008, Braesel *et al.* 2015). The atromentin structure is probably formed by RicA using 4-hydroxyphenylpyruvic acid as substrate and processed by RicF to afford leucoatromentin. The consecutive action of RicB and RicC will establish various *O*-methylation patterns, thus creating the diversity of rickenyl-type terphenyls (Fig. 15). Only the lack of an aminotransferase in the vicinity of the NRPS-like gene was unexpected as it is required for 4-hydroxyphenylpyruvate synthesis. We sought for homologous of the latter in the *H. rickii* genome and found at least four candidates that could fulfil the respective function, thus concluding that generation of the required phenylpyruvic acids from *L*-tyrosine (Braesel *et al.* 2015) is encoded at a distant locus, potentially also as part of primary metabolism. Furthermore, the aminotransferase located in the terrequinone-type cluster in *H. rickii* is potentially able to complement the terphenyl cluster, and thus might function as a cross acting enzyme in biosynthesis.

Based on the identified candidate *ric* BGC, homology searches among the other genomes were conducted. Five species (*A. truncatum*, *D. concentrica*, *H. pulicicidum*, *H. rubiginosum*, *J. multiformis*) contain up to two similar BGCs each, most of which contain various copies of *O*-methyltransferases (Fig. 13B). Among those, only two encode an aminotransferase showing that the presence of these genes does not follow a strict pattern. The predicted size of the BGCs varies widely (10–45 kbp) with some of them being extended by up to four cytochrome P450 monooxygenases implying heavily modified pathway products. Homology analysis of these BGCs with the clinker tool indicates that some copies of the *O*-methyltransferase genes might be the result of gene duplication events and that these BGCs have undergone significant rearrangements during speciation.

**Fig. 14:**
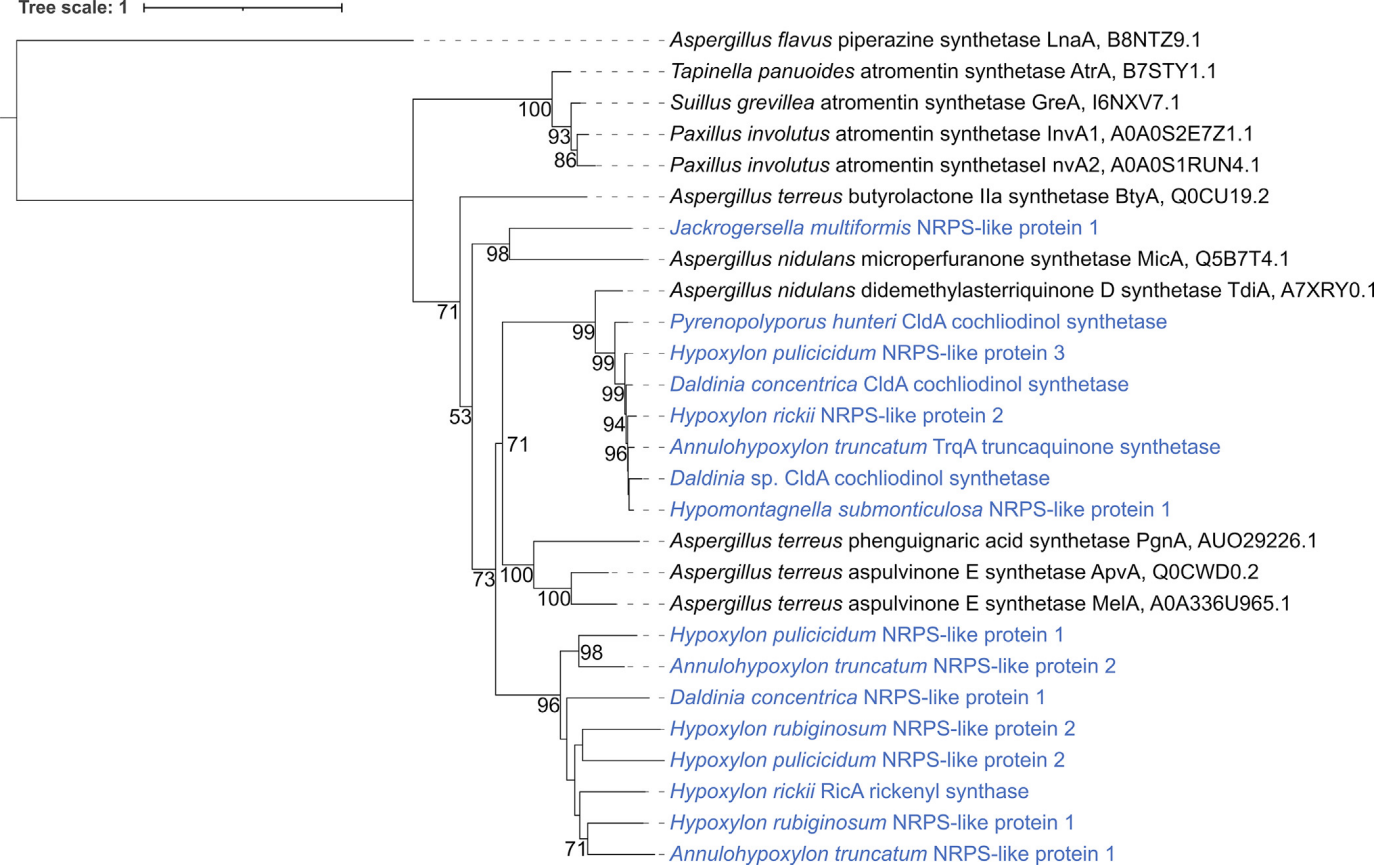
Phylogenetic analysis of NRPS-like enzymes from various origins using maximum-likelihood as optimality criterion. Ultrafast bootstrap support values of 1 000 replicates above 50 % are shown at corresponding nodes. Only support values above 95 % are deemed significant. Protein sequences from the *Hypoxylaceae* are highlighted in blue.

To deduce whether the unknown BGC products might share the same core scaffold, a phylogenetic reconstruction with known TE-containing NRPS-like enzymes and those found in the *Hypoxylaceae* was performed. As expected, one subclade contains the predicted asterriquinone-type enzymes alongside TdiA from the terrequinone pathway (Fig. 14). Only the NRPS-like enzyme identified in the *J. multiformis* genome appeared together with MicA (microperfuranone synthase from *Aspergillus nidulans*) with high bootstrap support, indicating that the products of these two enzymes might be related. As the other *Hypoxylaceae* derived NRPS-like enzymes form a monophyletic clade together with the rickenyl synthase RicA (which is supposedly an atromentin synthase), it appears possible that the products of these enzymes are all terphenyls. However, this generalisation needs to be verified by either isolating the respective products from the original fungus or systematically screening the core enzymes by heterologous expression strategies.

In general, biosynthetic pathways with TE-containing NRPS-like proteins are widespread in the *Hypoxylaceae* and occur across all major lineages. Some organisms even contain up to three homologs of the respective core enzyme. This shows that these types of pathways are highly conserved and are likely to have been derived from ancestral lineages, and that pathway duplication is common. The biological function of the pathway products therefore supposedly provides a selective advantage. Terphenyls are associated with antioxidative activities (Liu 2006, Kuhnert *et al.* 2015b), which could be related to their main function in the producer organism as oxidative stress commonly occurs in fungi through metabolic activity and external influences (Breitenbach *et al.* 2015). Conserving such metabolites might be a prerequisite for efficient distribution and adaptation, thus explaining their prevalent presence. Interestingly, no TE-containing NRPS-like genes have been discovered in the *X. hypoxylon* genome. However, Blastp searches have revealed homologs in other *Xylariaceae* species such as *X. grammica* (RWA06149), *X. flabelliformis* (TRX91999), *X. longipes* (RYC57524), *X. multiplex* (KAF2963097) and *Dematophora necatrix* [formerly *Rosellinia necatrix*, (Wittstein *et al.* 2020), GAP84749, GAP82384, GAP91480], indicating that such genes are also common in this family and that *X. hypoxylon* has lost them during evolution.

### Terpene pathways

#### Brasilanes

**Fig. 16:**
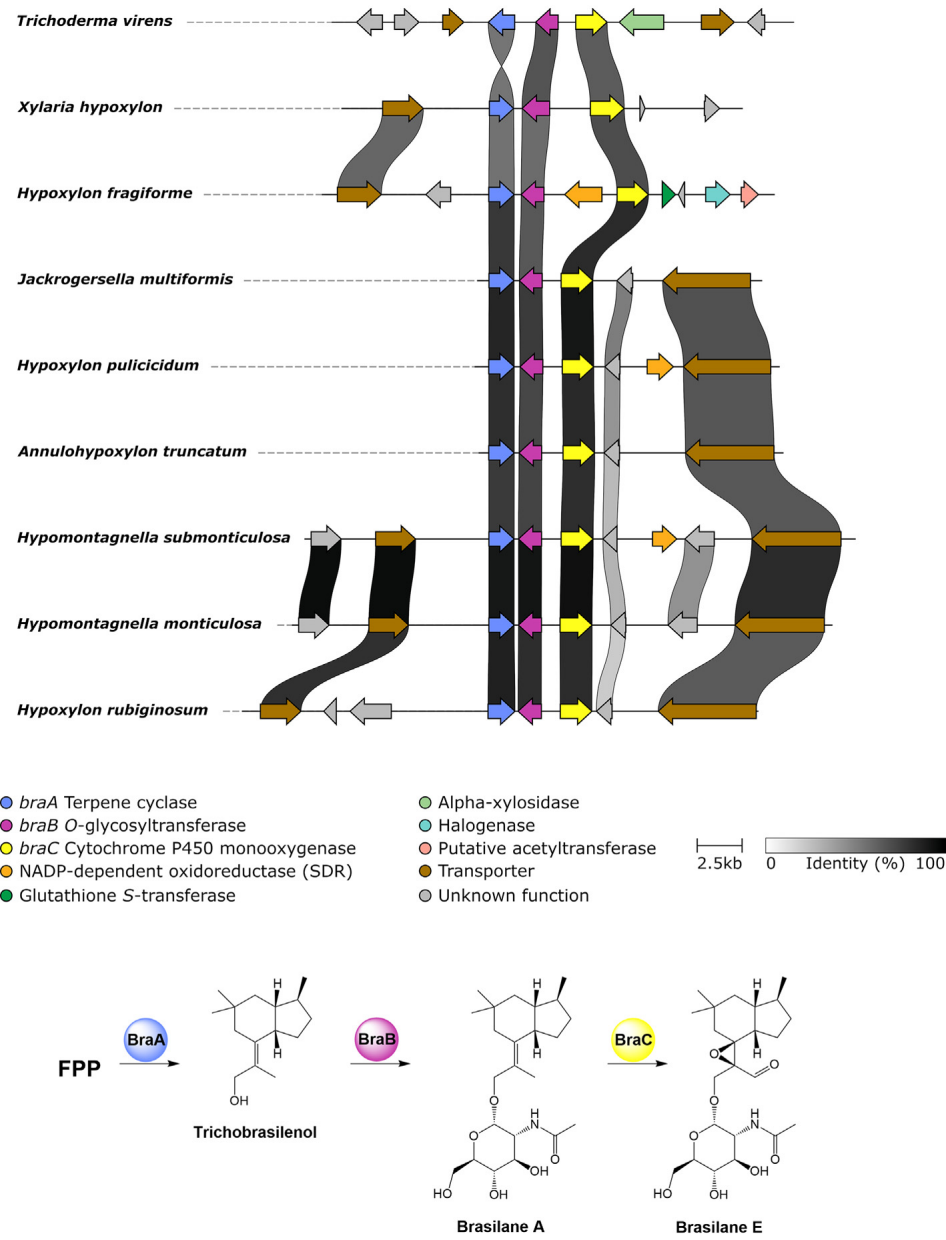
Synteny analysis of the brasilane biosynthetic gene clusters identified from various *Hypoxylaceae* species and other fungi and biosynthetic pathway for brasilane glycoside formation.

We recently reported the characterisation of the biosynthetic pathway for glycosylated sesquiterpenes of the brasilane-type in *A. truncatum*. These glycosides featured an unusual *N*-acetylglucosamine unit which is introduced by a dedicated promiscuous *N-*acetylglucosamine transferase, the first of its kind known in fungal secondary metabolism (Feng *et al.* 2020). The respective BGC (*bra*) consists of three core genes including a terpene cyclase, a P450 monooxygenase and the already mentioned glycosyltransferase. Homology searches showed that the BGC can be found in *X. hypoxylon* and various *Hypoxylaceae* species, such as *H. fragiforme*, *H. rubiginosum*, *H. pulicicidum*, *J. multiformis* and all sequenced *Hypomontagnella* species (Fig. 16). We observed the production of brasilane E in *H. fragiforme* and *H. pulicicidum,* proving that the respective homologs are functional (Fig. S13).

While the *bra* cluster architecture is highly conserved between most of the species, the brasilane BGC in *H. fragiforme* appears to be extended by various tailoring genes including a halogenase and a putative acetyltransferase. As brasilane E is produced in culture by this fungus, it appears unlikely that these additional genes are co-regulated. However, further investigations are necessary to confirm this hypothesis. We then looked for the occurrence of similar BGCs with the same set of core genes (*braA*, *braB*, *braC*) in other fungi by using the cblaster tool and found a number of hits in other *Hypoxylaceae* and *Xylariaceae* species as well as many *Trichoderma* (*Hypocreales*), *Talaromyces* (*Eurotiales*), *Monosporascus* (*Xylariales*) and *Pseudogymnoascus* (*Thelebolales*) species. As a representative, the *Trichoderma virens* BGC was included in the synteny analysis showing the high degree of conservation even across unrelated species (Fig. 16). This finding was no surprise as the respective *T. virens* terpene synthase is highly similar to TaTC6 from *T. atroviride* which has already been linked to the biosynthesis of brasilane scaffolds (Murai *et al.* 2019).

The natural function of brasilanes in the *Hypoxylaceae* has not been elucidated yet as the compounds did not show any antibacterial, antifungal or cytotoxic activity (Feng *et al.* 2020). However, the high degree of BGC conservation across this family and other fungal families in combination with an unusual glycosylation pattern of the molecules points towards an important, and as yet undiscovered, ecological role.

#### Triterpenoids

Triterpene natural products in fungi are common but few studies have correlated these compounds with the underlying biosynthetic genes. In the majority of cases, fungal triterpenes have been found to be derived from lanosterol, the first intermediate during ergosterol assembly (Schmidt-Dannert 2015). The producers sometimes carry an additional slightly modified copy of the lanosterol synthase (LSS) as shown for the biosynthesis of clavaric acid in *Hypholoma sublateritium* or helvolic acid in *Aspergillus fumigatus* (Godio & Martín 2009, Lv *et al.* 2017). More recently, it was shown that lanosterol from the primary copy of LSS can also provide the starting material for the biosynthesis of strongly modified triterpenes, such as the viridin congeners in *Hypoxylon cf. croceum* (originally identified as *Nodulisporium* sp.) (Wang *et al.* 2018, Sir *et al.* 2019). Interestingly, the underlying BGC was distantly located from this LSS, demonstrating that localisation of fungal triterpene BGCs can be challenging. We also recently reported the ubiquitous presence of a new type of fungal triterpene synthases, the squalene-hopene cyclases (SHC), which have been linked to the production of the potent antifungal compound enfumafungin in *Hormonema carpetanum* (Kuhnert *et al.* 2018).

So far, only a few triterpenes have been identified from the *Hypoxylaceae* and *Xylariaceae* including integracide derivatives from an endophytic *Hypoxylon* sp., demethoxyviridiol from *H. hinnuleum*, modified steroids from two unidentified *Xylaria* sp., and also a set of fernane glycosides named kolokosides from a *Xylaria* sp. (Deyrup *et al.* 2007, Song *et al.* 2014, Liang *et al.* 2018, Sir *et al.* 2019). Additionally, *D. concentrica* stromata are known for their significant amounts of concentricols, which constitute highly oxygenated squalene derivatives (Stadler *et al.* 2001b, Quang *et al.* 2002a). As the genome of the latter species did not contain additional copies of the squalene synthase and the primary gene did not have any biosynthetic genes in its proximity, the biosynthesis of concentricols remains obscure.

**Fig. 17:**
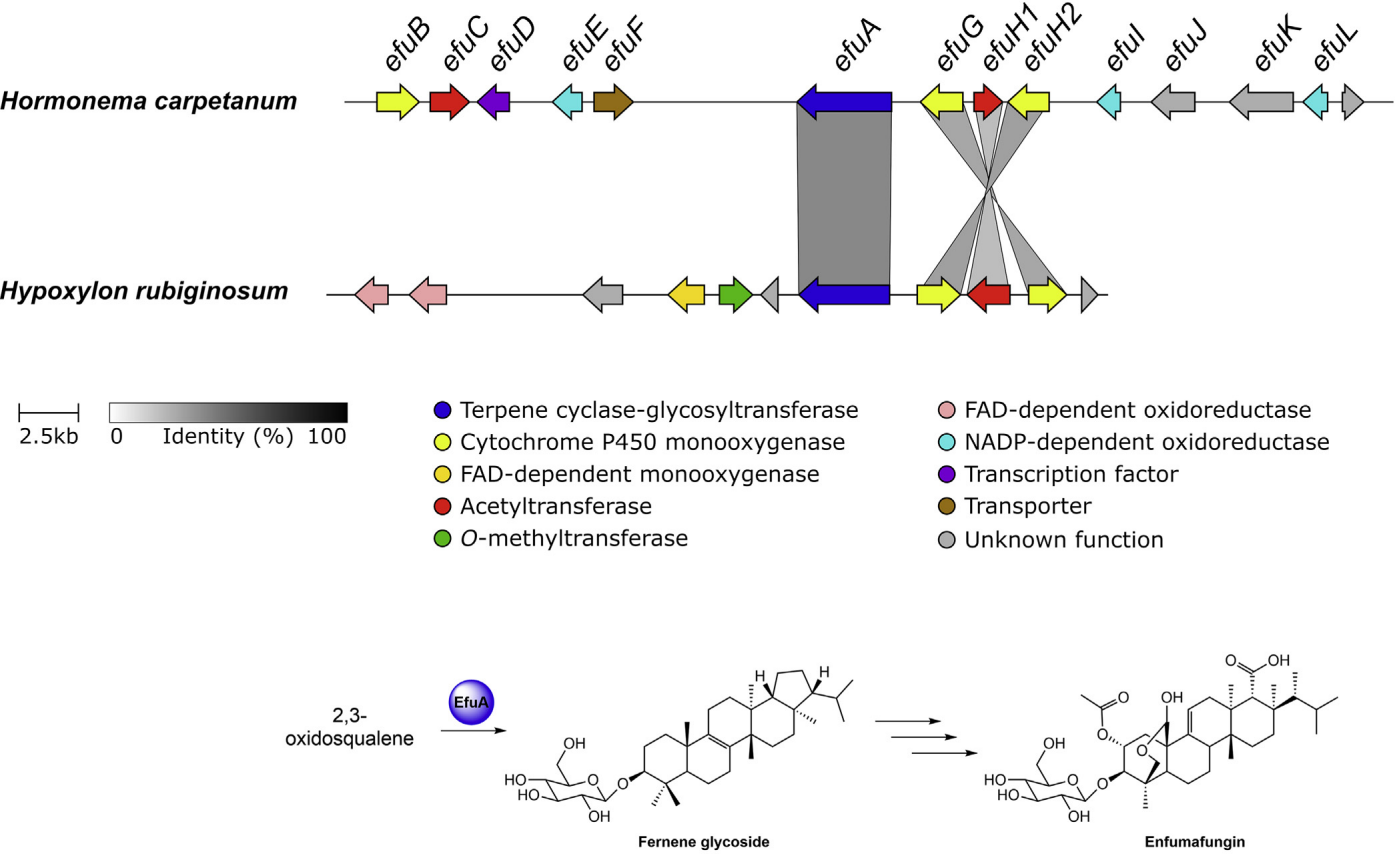
Synteny analysis of the re-annotated enfumafungin (*efu*) biosynthetic gene cluster known from *Hormonema carpetanum* and a homologous gene cluster identified in *Hypoxylon rubiginosum*. A general biosynthetic scheme for enfumafungin biosynthesis is given below (exact tailoring steps are not yet elucidated).

In contrast, similarity searches using LSS and SHC as templates revealed the presence of an SHC gene in *H. rubiginosum* which was surrounded by tailoring genes. The encoded SHC protein has similar structural features as the enfumafungin synthase and is also characterised by a glycosyltransferase domain. Therefore, it is very likely that the pathway product belongs to the fernane glycoside family, thus sharing structural similarities with enfumafungin. A synteny analysis between the *H. rubiginosum* fernane BGC and the enfumafungin BGC (*efu*) from *Hormonema carpetanum* showed that both BGCs share the same set of core genes including two P450 monooxygenases and an acetyltransferase (Fig. 17). No other gene was conserved, instead the *H. rubiginosum* BGC varies by containing an *O*-methyltransferase and an FAD-dependent monooxygenase. Two FAD-dependent oxidoreductases in the close proximity of the core genes might also be part of the BGC, potentially introducing further functional groups to the backbone. It is therefore likely that *H. rubiginosum* is able to produce novel fernane glycosides under the correct conditions. This is in particular interesting as most of the known fernanes possess potent activities mainly against fungi and therefore could be of pharmaceutical relevance (Kuhnert *et al.* 2018). This discovery also adds further information on the diversification of fernane-type metabolites in fungi.

In addition to this BGC, an LSS homologue was located in *X. hypoxylon* with similarities to the protostadienol synthase from *Aspergillus fumigatus* involved in helvolic acid biosynthesis (Lv *et al.* 2017). The core gene was clustered with genes encoding a P450 monooxygenase, an *O*-acetyltransferase and a putative epoxide hydrolase, all of which had no significant sequence similarity with tailoring enzymes encoded by the helvolic acid BGC. No further triterpenoid-related BGCs were identified in the genomes of the investigated fungi including homologues of the demethoxyviridin BGC (*vid*). The latter was originally identified from *H*. *cf*. *croceum* which is phylogenetically related to *H. pulicicidum* (Sir *et al.* 2019). The absence of the *vid* BGC in *H. pulicicidum* indicates that the biosynthesis of viridin congeners is restricted to members of the *H. croceum* complex. The rare presence of triterpenoid BGCs in the analysed genomes of the *Hypoxylaceae* correlates well with the rare observation of respective compounds in these organisms.

### Alkyl citrates

**Fig. 18:**
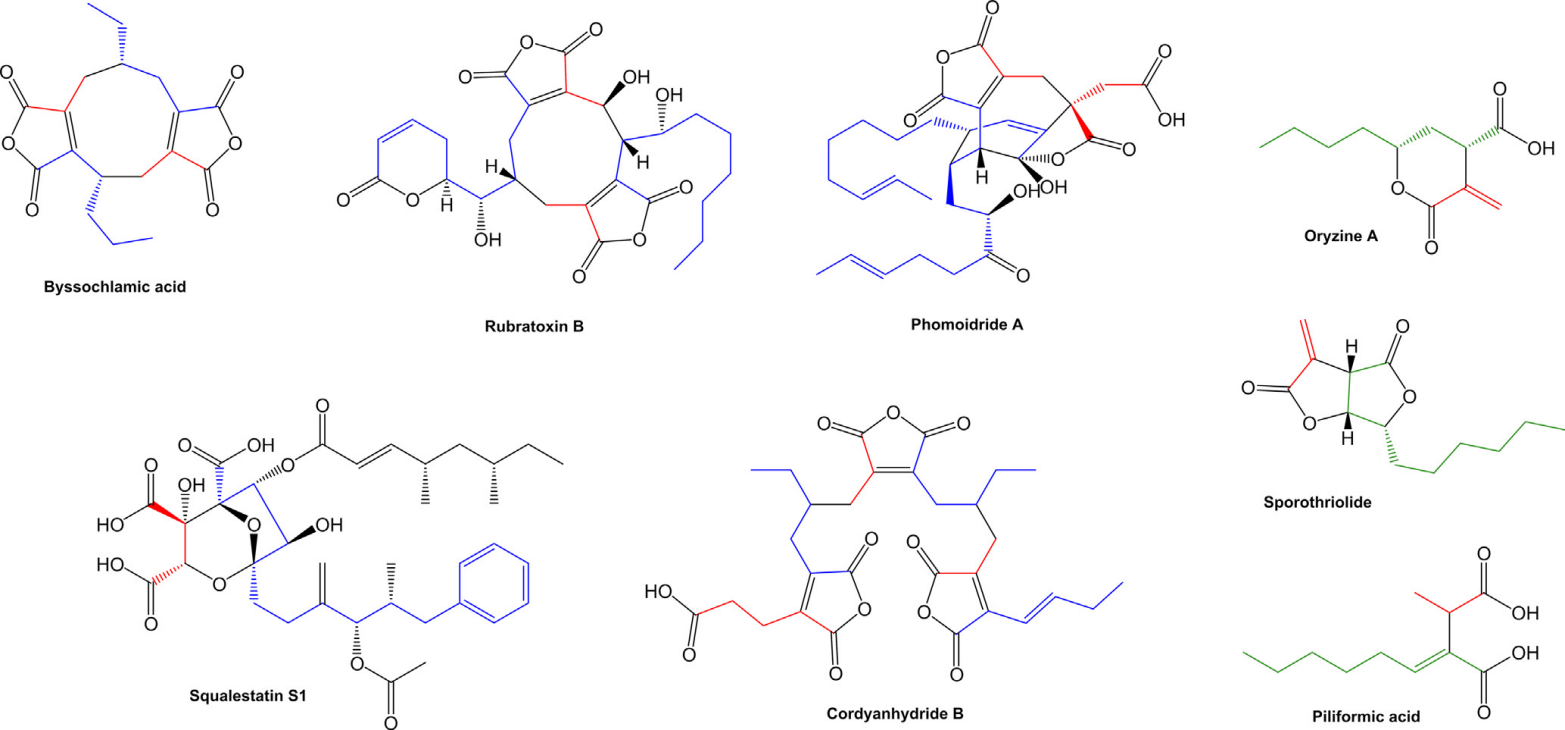
Representative structures of fungal alkyl citrates. Carbon chains derived from citric acid cycle intermediates (red), highly reducing polyketide synthases (blue) and fatty acid synthases (green) are highlighted.

Citrate synthases (CS) are important enzymes involved in the citric acid cycle where they catalyse the condensation of oxaloacetate with acetyl-CoA to yield citrate (Wiegand & Remington 1986). During the early studies on the biosynthesis of the fungal toxin byssochlamic acid it was proposed that related enzymes also play a role in secondary metabolism (Barton & Sutherland 1965). This hypothesis was later proved by heterologous expression studies of the respective pathway showing that the BGC encoded CS uses a hrPKS-derived hexenoate and oxaloacetate to form an alkyl citrate intermediate. Subsequent activity of a dehydratase establishes a maleic acid anhydride monomer that can dimerise in the presence of a ketosteroid isomerase-like protein (Williams *et al.* 2016). The involvement of CS reactions has also been reported for other fungal anhydrides such as phomoidride and rubratoxin (Fujii *et al.* 2015, Bai *et al.* 2017). In addition to compounds bearing an anhydride moiety, biosynthetic studies on the potent squalene synthase inhibitor squalestatin showed that CSs also occur in other fungal biosynthetic pathways. In the latter example, the CS uses a hexaketide as acceptor substrate (Bonsch *et al.* 2016, Liu *et al.* 2017b). We also identified a CS containing BGC in *Aspergillus oryzae* that is putatively responsible for the production of the alkyl citrates oryzine A and B (Wasil *et al.* 2018). In general, CS related to secondary metabolism have evolved different selectivity for alkyl chains and therefore are also referred to as alkyl citrate synthases (ACS) (Fujii *et al.* 2015). A selection of fungal alkyl citrates is depicted in Fig. 18.

**Fig. 19:**
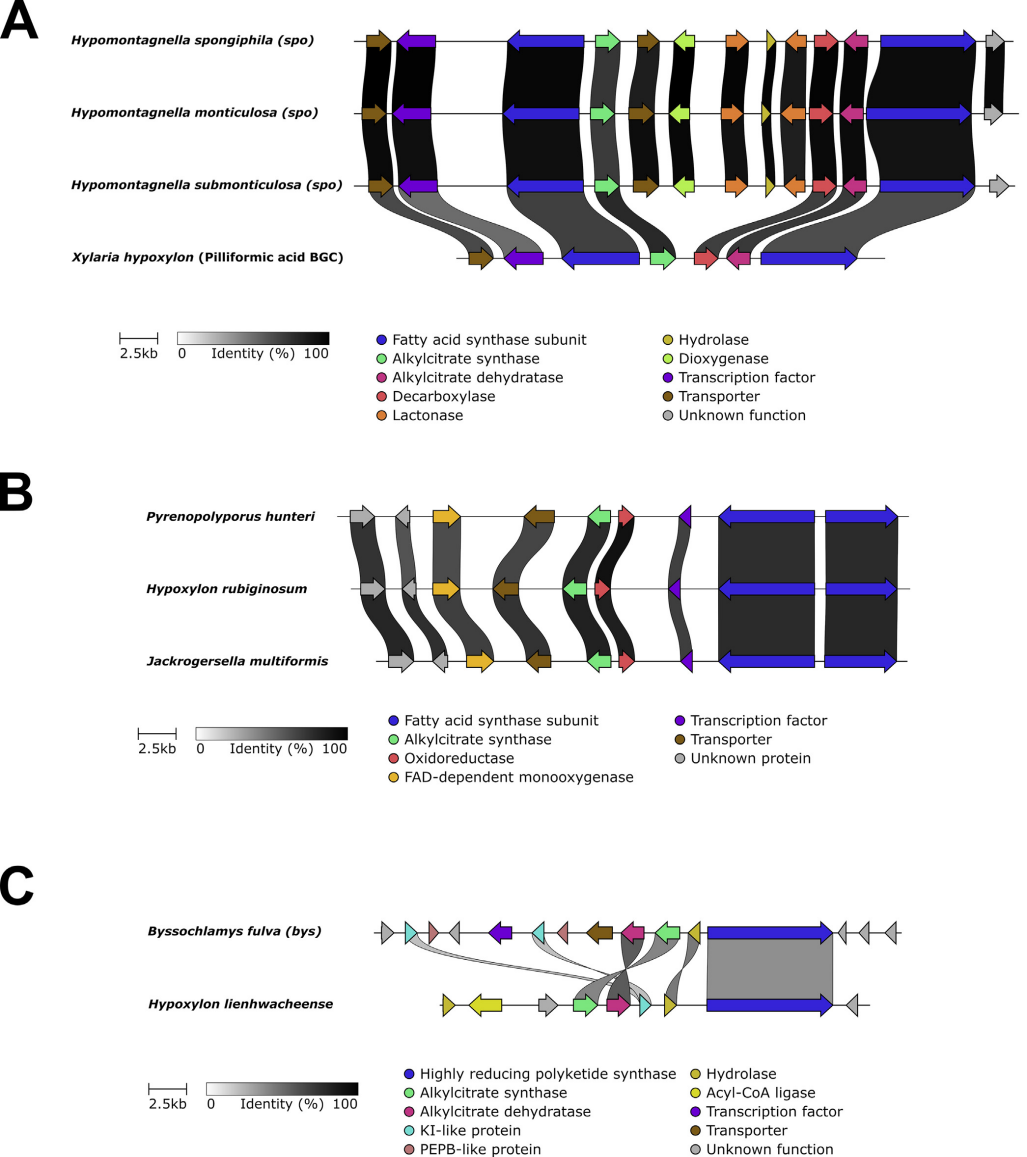
Synteny analysis of identified alkyl citrate biosynthetic gene cluster (BGC) in the analysed *Hypoxylaceae* species and *Xylaria hypoxylon*. A; sporothriolide (*spo*) and piliformic acid BGC. B; Conserved alkyl citrate BGC with unknown product represented by three species (occurs in all *Hypoxylaceae* genomes). C; byssochlamic acid (*bys*) and putative maleic acid anhydride BGC.

While fungal anhydrides and compounds related to squalestatin have not been reported from the *Hypoxylaceae* so far, the furofurandione sporothriolide discovered in cultures of *Hypomontagnella* species was suspected to require an ACS for its biosynthesis (Surup *et al.* 2014). We recently demonstrated that sporothriolide and derivatives are indeed formed by a BGC (*spo*, Fig. 19A) that relies on the activity of an ACS (SpoE). In contrast to previous reported ACSs, SpoE uses a decanoyl CoA as substrate which is derived from cluster-encoded fatty acid synthase (FAS) components (Tian *et al.* 2020). Due to the unique combination of the biosynthetic core genes, the sporothriolide pathway does not fit into any of the established BGC categories and we therefore introduce the term alkyl citrate pathways to cover all compounds that require a dedicated ACS for their formation.

Searches for ACS containing BGCs revealed that at least one related pathway is present in each analysed organism. All *Hypoxylaceae* contain a highly conserved alkyl citrate BGC characterised by genes encoding fatty acid synthase subunits, ACS, FAD-dependent monooxygenase and an oxidoreductase with protein sequence similarity to Mfr1 and Mfr2 of the squalestatin BGC (Fig. 19B) (Lebe & Cox 2019). A respective BGC is not present in *X. hypoxylon*, and cblaster analysis indicates that other members of the *Xylariaceae* lack such a BGC as well. As sporothriolides are the only reported alkyl citrates from the *Hypoxylaceae* the product of this BGC is unknown, but the high conservation across the family implies product formation under undetermined conditions. We are currently investigating the pathway by heterologous expression studies and preliminary results showed that the squalene synthase inhibitor CJ-13,982 is an intermediate (data not shown) (Watanabe *et al.* 2001).

In addition to the highly conserved alkyl citrate BGCs, all analysed *Hypomontagnella* species carry the sporothriolide BGC (*spo*) as previously reported (Fig. 16A) (Tian *et al.* 2020). Biosynthesis of sporothriolide and congeners involves the activity of a dehydratase, decarboxylase, dioxygenase and lactonases in addition to the core ACS and FAS. Homologous BGCs cannot be found in other studied members of the *Hypoxylaceae* but exist in *X. hypoxylon*. This BGC differs from *spo* by the absence of a dioxygenase and lactonase genes. *Xylaria hypoxylon* is a known producer of the alkyl citrate piliformic acid and the BGC is in accordance with its proposed biosynthetic logic (Tian *et al.* 2020). The cblaster tool also identified piliformic acid-type BGCs in other *Xylariaceae* such as *X. flabelliformis*, *X. longipes* and *Rosellinia necatrix* indicating that such a BGC is more common in the *Xylariaceae*. The *spo* BGC is also highly similar to the putative oryzine BGC in *A. oryzae* (which is also present in many other *Aspergillus* species) sharing the same set of genes (Wasil *et al.* 2018). Structural differences between sporothriolides and oryzines are attributed to the catalytic activities of the dioxygenase and lactonases (Tian *et al.* 2020).

Another alkyl citrate BGC was located in *H. lienhwacheense*. This BGC carries an hrPKS instead of FAS components and shows similarity to the characterised byssochlamic acid BGC (Williams *et al.* 2016) (Fig. 19C). We identified cordyanhydrides in the fermentation broth and stromata of the fungus (data not shown) and therefore assume that this BGC is responsible for their formation. Interestingly, a PKS-dependent alkyl citrate BGC only occurred in this single species in our dataset rendering it a supposedly rare BGC-type within the *Hypoxylaceae*. The biosynthesis of cordyanhydrides is currently under investigation in our groups.

Our investigation shows that FAS-dependent alkyl citrate pathways are very common in the *Xylariales*. This is not a surprise as CS and FAS are essential parts of the primary metabolism and duplication events of these genes are more likely to lead to new secondary metabolism pathways. Diversity of alkyl citrate pathways is probably initially driven by the specificity of the ACS that can change through mutations in the active site. It can be speculated that changes in the catalytic cavity allowed the acceptance of larger alkyl chains including fatty acids and polyketids which led to the formation of new alkyl citrates (Yin *et al.* 2021). Larger carbon skeletons will enable further modification by tailoring enzymes, thus further driving the evolution of alkyl citrate pathways.

### Hybrid pathways

#### Cytochalasans

Cytochalasans are a large class of fungal metabolites distinguished by the presence of an octahydroisoindolone core fused to a macrocycle, with often cytotoxic properties. They are derived by fusion of an amino acid with a polyketide. Their cellular target is the actin cytoskeleton, where they inhibit actin polymerisation by binding to a cleft between the actin subdomains 1 and 3, but the exact mode-of-action might differ between the different derivatives. As such, cytochalasins (in particular cytochalasin B and D) have been widely employed to study actin-dependent cellular processes (Scherlach *et al.* 2010). Biosynthetically, they are derived from hybrid PKS-NRPS synthetases which can use a wide variety of (usually aromatic) amino acids, and vary the chain-length and methylation pattern of the polyketide. To establish the typical octahydroisoindolone core, the activities of a hybrid PKS-NRPS, *trans*-enoyl reductase, α,β-hydrolase and a Diels-Alderase are required (Skellam 2017, Hantke *et al.* 2020, Zhang *et al.* 2021). Additional modifications are later introduced *via* a diverse range of tailoring enzymes, such as cytochrome P450 monooxygenases, Baeyer-Villiger monooxygenases, oxidoreductases and acetyltransferases, which are responsible for the diversification of cytochalasins (Wang *et al.* 2019a).

**Fig. 20:**
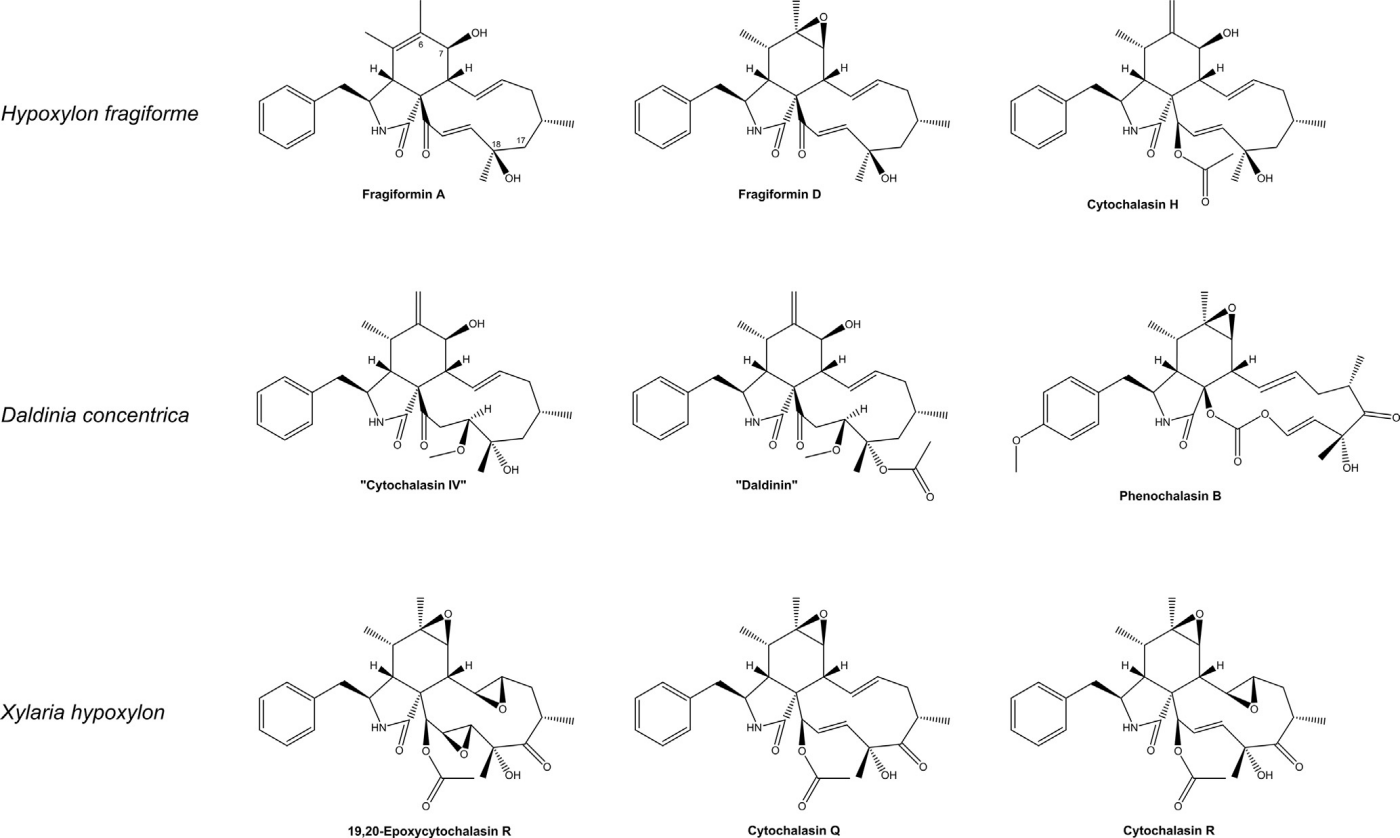
Cytochalasins reported from *Hypoxylon fragiforme*, *Daldinia concentrica* and *Xylaria hypoxylon*.

**Fig. 22:**
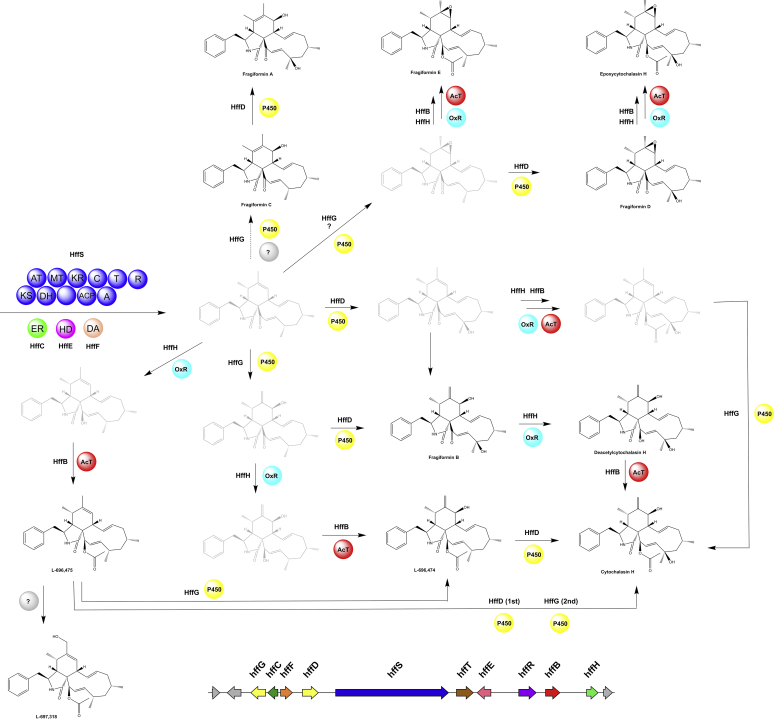
Proposed biosynthetic pathway for the formation of cytochalasins in *Hypoxylon fragiforme*. Putative intermediates are shown in grey. Function of HffD and HffG have been elucidated in a previous study (Wang *et al.* 2019b). The biosynthetic gene cluster (MN477016.1) is shown at the bottom.

There are a large number of reports about cytochalasin producing *Hypoxylaceae* and allied families. More than 30 different cytochalasins including the HIV-1 protease inhibitor L-696,474 (18-dehydroxy cytochalasin H) have been identified from *Hypoxylaceae*, all of which were obtained from *Daldinia* spp., *H. fragiforme* and members of the *H. fuscum* complex (Dombrowski *et al.* 1992, Hashimoto & Asakawa 1998, Stadler *et al.* 2001a, 2006, Qin *et al.* 2006, Yuyama *et al.* 2018, Yang *et al.* 2018, Narmani *et al.* 2019, Kretz *et al.* 2019, Van Trung *et al.* 2019, Wang *et al.* 2019b, Lambert *et al.* 2021) (Figs 20, S14). We previously reported the *hff* cytochalasin BGC from *H. fragiforme* and proved the function of its cytochrome P450 monooxygenases through a combinatorial biosynthesis approach in combination with the pyrichalasin H BGC (*pyi*) from *Pyricularia grisea* (Wang *et al.* 2019b). In addition to the core and P450 monooxygenase genes, the BGC also contains an NADP-dependent oxidoreductase and an acetyltransferase. This manifest of genes allows for a rational explanation of the biosynthesis towards cytochalasin H and other known derivatives (Fig. 22). Only the epoxidation found in most of the fragiformins between C-6 and C-7 is in disagreement with the results of the combinatorial biosynthesis study, which demonstrated that the P450 monooxygenase HffG is not able to introduce an epoxide functionality at this position on the pyrichlasin backbone (Wang *et al.* 2019b). However, as the latter is not the natural substrate for HffG, it remains possible that the enzyme can also catalyse epoxidation when other substrates are available.

**Fig. 21:**
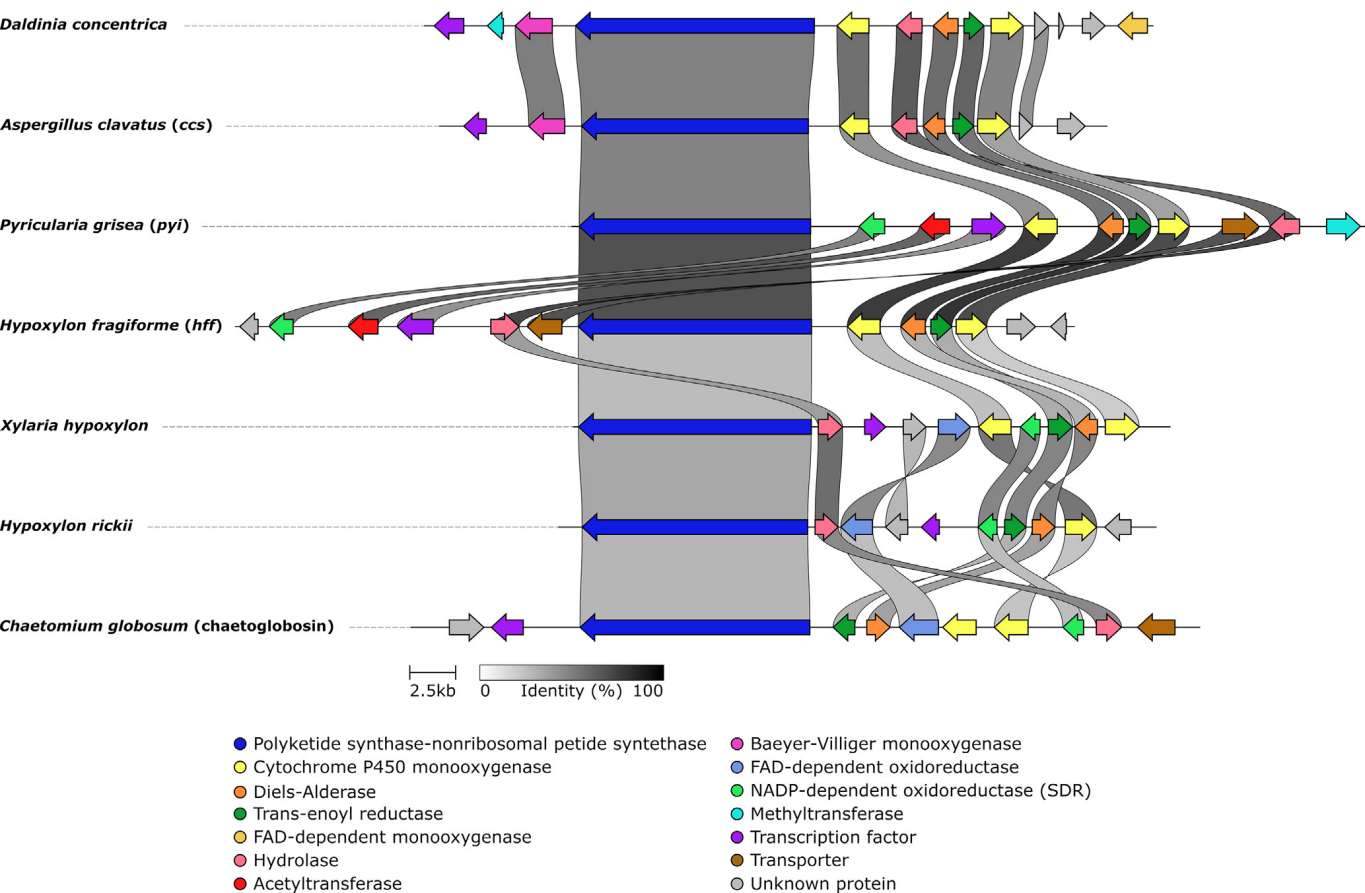
Synteny analysis of cytochalasin biosynthetic gene cluster localised in the genomes of *Hypoxylaceae* species and *Xylaria hypoxylon*. Note that the BGC from *X. hypoxylon* is located on the end of a scaffold potentially missing related genes.

To study the distribution of cytochalasin BGC across the *Hypoxylaceae* and *X. hypoxylon*, homology searches were conducted leading to the identification of three more BGCs (Fig. 21). As expected, the known cytochalasin producers *D. concentrica* and *X. hypoxylon* contained the respective BGC, but also *H. rickii*. The production of secondary metabolites in the latter has been extensively investigated in the past, but so far no cytochalasins have been identified (Kuhnert *et al.* 2015b,a, Surup *et al.* 2015, 2016, 2018a). There are various records about cytochalasins from *D. concentrica* (Stadler *et al.* 2001a, Quang *et al.* 2002b, Qin *et al.* 2006) enabling the correlation of those with the identified BGC. Four of those (cytochalasin I–IV) were only tentatively identified by HPLC-MS analysis (Stadler *et al.* 2001a), but could be explained with the present set of genes, except for the reduction of the alkene between C-19 and C-20, which might be catalysed by an enzyme encoded by one of the unknown genes (Fig. S15). Another cytochalasin incorrectly termed “daldinin” (this name originally refers to a group of azaphilones known from some *Daldinia* and *Hypoxylon* species (Hashimoto *et al.* 1994, Quang *et al.* 2004a) carries an acetate functionality at C-18 (Van Trung *et al.* 2019). There is no respective acetyltransferase encoded in the BGC, but in case of a correct identification of the producer [note that there is no reliable report of *D. concentrica* from Asia (Stadler *et al.* 2014)], an unclustered acetyltransferase might be involved in the product formation.

Furthermore, phenochalasin B was also obtained from stromata of *D. concentrica* (Quang *et al.* 2002b). The characteristic features of this molecule include methoxytyrosine as amino acid building block, a carbonate ring expansion and a carbonyl moiety at C-17. The ring expansion can be linked to the Baeyer-Villiger monoxygenase (BVMO) encoded in the BGC and the methoxytyrosine could be a result of the activity of the methyltransferase located next to the BVMO. The high similarity between the *D. concentrica* and *A. clavatus* cytochalasan BGC further implies closely related pathway products. Cytochalasin E is formed by the *A. clavatus* BGC and only differs from phenochalasin B by containing phenylalanine as building block indicating that phenochalasin B is the actual pathway product of the *D. concentrica* BGC. Whether the other reported cytochalasins from the same species are shunt-products of this pathway or are derived from strain-specific BGCs cannot be answered in the course of this study. It is noteworthy that *H. fragiforme* cytochalasins always differ from those produced by the *Daldinia* species in regard to the stereochemistry at C-18. In the absence of a hydroxyl functionality the stereochemistry at this carbon is likely determined by the trans-acting enoyl reductase (Roberts *et al.* 2017) offering the possibility to further study structure dependent stereoselectivity of trans-ERs.

The cytochalasin BGCs from *H. rickii* and *X. hypoxylon* show a high degree of similarity, except for the absence of a second P450 monooxygenase gene in *H. rickii*. Unfortunately, the *X. hypoxylon* cluster was located at the end of a scaffold, therefore additional genes might have been missed.

While cytochalasin production is commonly encountered in the genus *Xylaria* (Dagne *et al.* 1994, Abate *et al.* 1997, Zhang *et al.* 2014, da Silva *et al.* 2019) there is only one reference for the isolation of these compounds from *X. hypoxylon* (Espada *et al.* 1997)*.* The producing strain, however, was cultivated from a soil sample and the identity was not confirmed by appropriate morphological and molecular methods, therefore it can potentially represent a different species. Most of the respective reported cytochalasins from this fungus were acetylated and highly oxygenated containing up to three epoxide functionalities (Fig. 20). The set of genes discovered here would not account for such modifications, but as mentioned before the BGC might be partially incomplete. To determine if the same compounds were produced by the genome-sequenced strain, a screening was conducted. No obvious cytochalasin production was observed but mass searches of LCMS data revealed the likely presence of related compounds, one of which could correspond to cytochalasin E (Fig. S16). The biosynthesis of the latter can be partially explained by the BGC, except for the ring expansion.

**Fig. 23:**
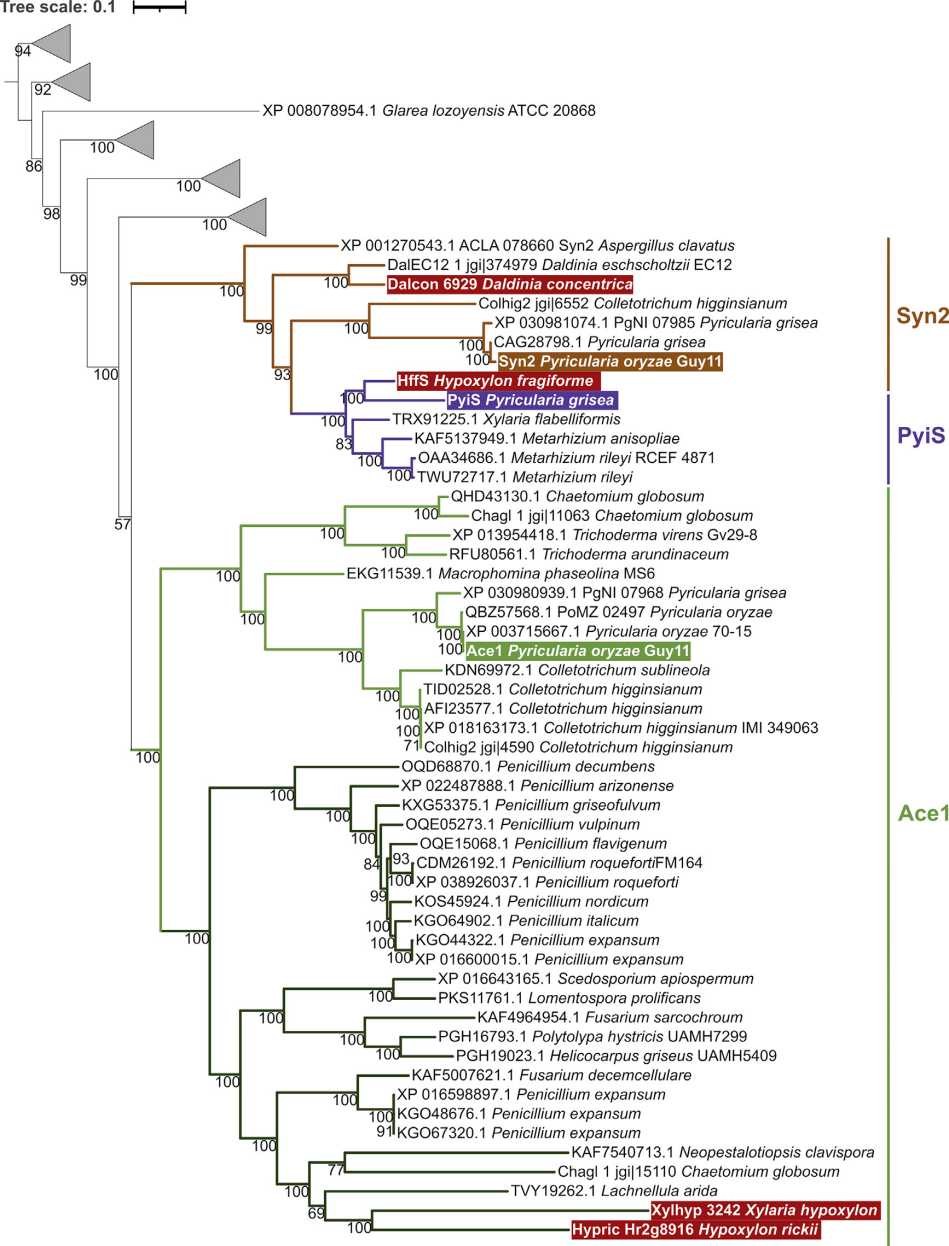
Maximum-likelihood tree of hybrid PKS-NRPS enzymes involved in cytochalasan biosynthesis, rooted with enzymes involved in biosynthesis of equisetin-like compounds. *Hypoxylaceae* homologues are highlighed in red. The paralogous Ace1 and Syn2 hybrids from *Pyricularia oryzae*, and PyiS from *P. grisea*, are highlighted in green, orange and purple respectively. Clades are coloured to highlight paralogy. The Ace1 clade can be further divided into two sub-clades of paralogues (light and dark green). Clades with distant uncharacterised homologues of cytochalasan synthases are collapsed. Branch support indicated is the result of ultrafast bootstrap. Only support values above 95 % are deemed significant.

The distribution of cytochalasin BGCs in the *Hypoxylaceae* shows that biosynthesis of the compounds is not conserved across the family but might be more prominent in certain genera such as *Daldinia* based on the number of known producers (Stadler *et al.* 2014, Narmani *et al.* 2019, Wongkanoun *et al.* 2019). As evidenced by the clinker analysis the similarity of the cytochalasin BGCs between *Xylariales* species does not reflect the actual relationship of the species (Fig. 21). This can be best observed for the BGCs identified from the very closely related species *H. fragiforme* and *H. rickii*. The cytochalasin BGC from the latter shares higher similarity with the *X. hypoxylon* BGC both in gene content as well as protein sequence identity than with the BGC from *H. fragiforme*. Interestingly, similar observation can be also made across completely unrelated species. For instance, the pyrichalasin BGC (*pyi*) from *Pyricularia grisea* contains almost the same set of genes as the *hff* BGC from *H. fragiforme* and the respective protein sequences show an identity of around 70 %, and heterologous complementation studies have shown that several *hff* genes are active when expressed in the context of the *pyi* cluster. Also, the BGC from *D. concentrica* shares a much higher similarity with the *ccs* BGC from *Aspergillus clavatus* than with other cytochalasin BGCs from the *Xylariales* (Fig. 21). Evolutionary studies of the related *ACE1* BGC in *Pyricularia oryzae* indicated that this BGC is prone to whole BGC duplication and rearrangements (Khaldi *et al.* 2008, Moore *et al.* 2014). In *P. oryzae* the *ACE1* BGC contains two hybrid PKS-NRPS genes, *ACE1* and *SYN2*, and is a rearrangement between the *ACE1* and *SYN2* BGCs after whole BGC duplication. The discrepancy observed in the *Hypoxylaceae* corresponds to the presence of either the *ACE1* or *SYN2* paralogous BGC (Fig. 23). The *pyi* BGC in *P. grisea* appears to be another paralogous BGC (Fig. 23). It will be also necessary to sequence more species of the different *Hypoxylaceae* genera to see how common these paralogous cytochalasin BGCs are in the family. In cases where transcription factors are encoded in the cluster, ectopic expression strategies are a viable approach to induce or increase the production of cytochalasans, which would be in particular useful for the identification of intermediates and new congeners (Hantke *et al.* 2019).

#### Meroterpenoids

**Fig. 24:**
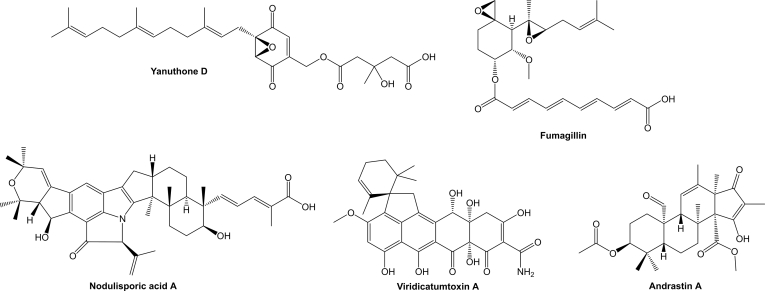
Structures of representative fungal meroterpenoids.

Meroterpenoids are often complex hybrid metabolites that consist of a terpene fused to products from non-terpenoid origins (Fig. 24). The latter can be derived from PKS (*e.g.* austinol, andrastin A, mycophenolic acid), NRPS (*e.g.* penigequinolones) or indoles (*e.g.* penitrem A, lolitrem B, nodulisporic acid A) (Matsuda & Abe 2016, Van de Bittner *et al.* 2018). The corresponding BGCs are highly diverse and usually contain a relatively high number of tailoring genes, in particular oxygenases and oxidoreductases. In most meroterpenoid biosynthetic pathways, the core reaction involves the activity of a prenyl transferase on a polyketide precursor, which later can be followed by the action of a terpene cyclase (TC) (Matsuda & Abe 2016). Alternatively, the terpene core can be connected by an intermolecular Diels-Alderase as observed during the biosynthesis of tropolonic sesquiterpenes, such as xenovulene A and eupenifeldin, or a polyketide chain is transferred to the terpene backbone by an acyl transferase as shown for the biosynthesis of fumagillin (Lin *et al.* 2013, Schor *et al.* 2018, Chen *et al.* 2019b). Due to these key chemical features meroterpenoid pathways can be traced in fungal genomes by genome mining approaches.

**Fig. 25:**
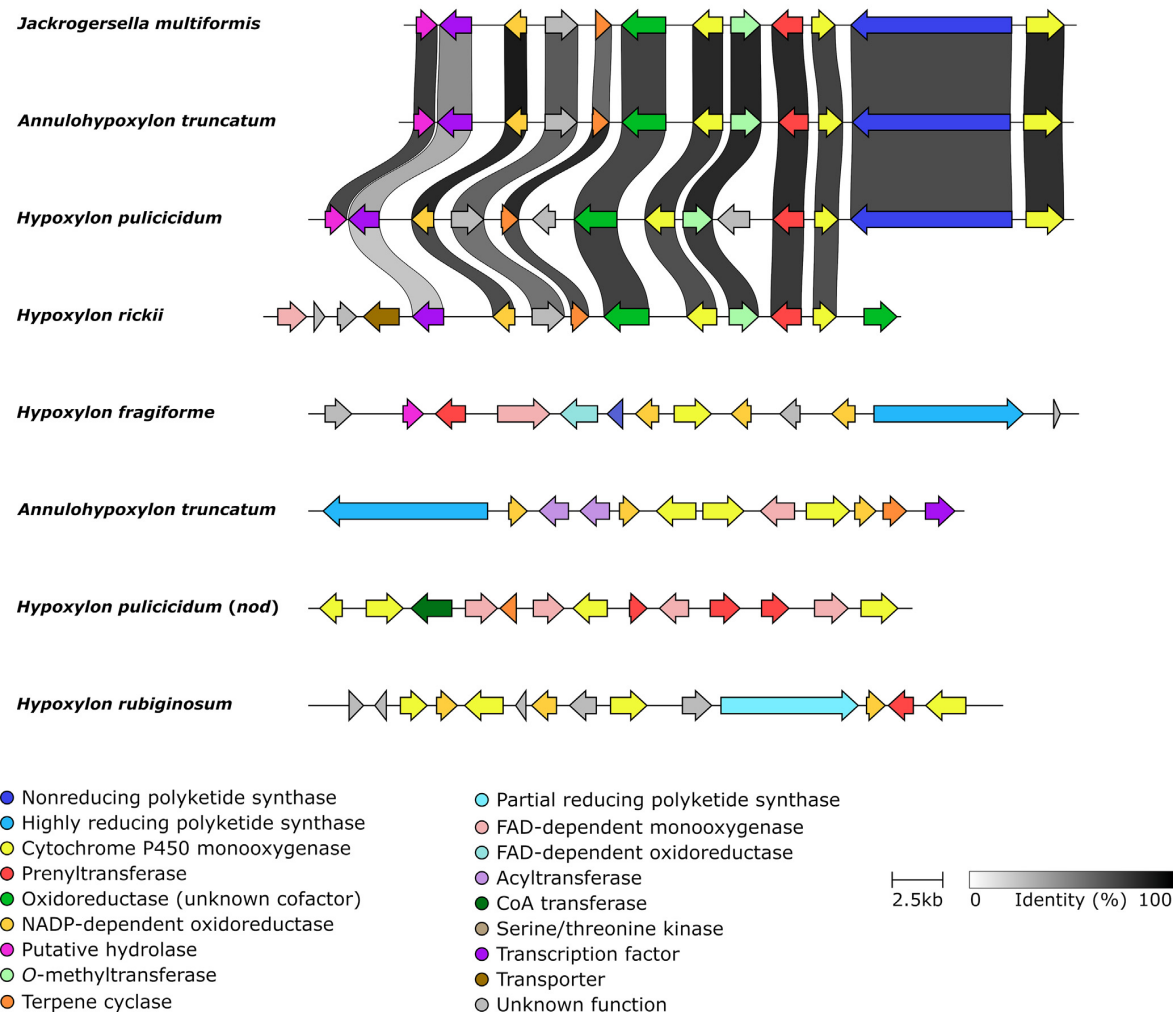
Synteny analysis executed on meroterpenoid biosynthetic gene clusters (BGC) identified in the *Hypoxylaceae*. Only cluster found in *Jackrogersella multiformis*, *Annulohypoxylon truncatum*, *Hypoxylon pulicicidum* and *H. rickii* showed significant similarity between each other. *nod*: nodulisporic acid BGC [incomplete cluster complemented by previously published sequence (Van de Bittner *et al.* 2018)].

So far meroterpenoids have been rarely reported from the *Hypoxylaceae* with the insecticidal nodulisporic acids being the only prominent representative of this class of natural products (Bills *et al.* 2012). The biosynthesis of the latter in *H. pulicicidum* has been partially elucidated and resembles those of other known indole diterpene pathways (Van de Bittner *et al.* 2018). As expected, our genome sequenced strain of *H. pulicicidum* (identical to that of Van de Bittner *et al.* 2018) contained the nodulisporic acid BGC (*nod*). Unfortunately, this BGC was incompletely assembled as parts of the BGC were located at the end of two different scaffolds (Fig. 25). Homology searches did not reveal the presence of similar BGCs in the other genomes, indicating that nodulisporic acid production might be restricted to *H. pulicicidum* or closely related species. We then further analysed the genomes to look for other putative indole-type meroterpenoid BGCs, but no related BGCs could be traced, indicating that production of indole diterpenes is rare in the *Hypoxylaceae*.

In addition to indole-type meroterpenoid pathways, we also searched for other prenyltransferase-containing BGCs and identified a variety of PKS-associated BGCs. Three of those present in *A. truncatum*, *J. multiformis* and *H. pulicicidum* show significant similarity between each other and a BGC from *H. rickii* that lacks a PKS gene. The core enzyme in the BGCs of *A. truncatum*, *J. multiformis* and *H. pulicicidum* is an nrPKS with an SAT-KS-AT-PT-ACP-(*C*-MeT)-TE domain structure (Fig. 25). The 43–44 % similarity of this PKS to the meroterpenoid PKSs MapC of the mycophenolic acid pathway and AdrD of the andrastin A pathway point towards 5-methylorsellinic acid or 3,5-dimethylorsellinic acid as the pathway precursor. Further modifications are likely established by the three encoded P450 monooxygenases, various oxidoreductases, the *O*-methyltransferase and the terpene cyclase. As there is no significant protein sequence similarity between the tailoring enzymes of known meroterpenoid pathways (*e.g.* mycophenolic acid, anditomin) and those identified in the *Hypoxylaceae,* further structure prediction is not feasible, but the BGCs likely encode the production of a compound which differs from those of related studied gene clusters.

The presence of a highly similar BGC in *H. rickii* that lacks the PKS core enzyme is remarkable in particular because all other genes have been conserved and the BGC is apparently expanded by additional genes such as an oxidoreductase, transporter and putatively an FAD-dependent monooxygenase. In theory it is unlikely that the BGC by itself is able to form a product (unless the prenyltransferase accepts substrates such as tyrosine), but it might cross-act on other PKS pathway products. There is no protein with significant protein level similarity to the meroterpenoid nrPKS from the related cluster encoded in the genome of *H. rickii*, but the fungus is, for example, able to produce orsellinic acid as part of the azaphilone BGCs (see previous section), which could be a suitable substrate for the meroterpenoid pathway. This also aligns well with the absence of orsellinic acid synthases in the genomes of *A. truncatum*, *J. multiformis* and *H. pulicicidum*.

A further putative meroterpenoid BGC was located in the genome of *H. fragiforme*. This BGC is characterised by the rather unusual combination of a prenyltransferase and highly reducing PKS (hrPKS) alongside various oxidoreductases, a P450 and FAD-dependent monooxygenase (Fig. 25). So far, hrPKS have been rarely identified in meroterpenoid pathways and were either accompanied by an nrPKS (Zhang *et al.* 2018) or an acyltransferase instead of the prenyltransferase as seen in the biosynthesis of fumagillin (Lin *et al.* 2013). Whether the prenyltransferase acts on the PKS product or is only coincidentally located in its proximity cannot be answered without transcriptomic data and identification of the pathway product. However, this BGC in *H. fragiforme* could potentially represent a new type of meroterpenoid BGC.

We also found a further putative meroterpenoid BGC in *A. truncatum*, which structurally resembles the fumagillin BGC, but does not show any significant protein level similarity to the latter. It encodes an hrPKS, a terpene cyclase with 66 % protein similarity to the aristolochene synthase involved in PR-toxin formation (Brock & Dickschat 2013), two acyltransferases, three P450 monooxygenases, an FAD-dependent monooxygenase and three oxidoreductases (Fig. 25). The presence of two acyltransferases in this BGC is in particular intriguing as it could potentially lead to a multi-acylated terpene core.

**Fig. 26:**
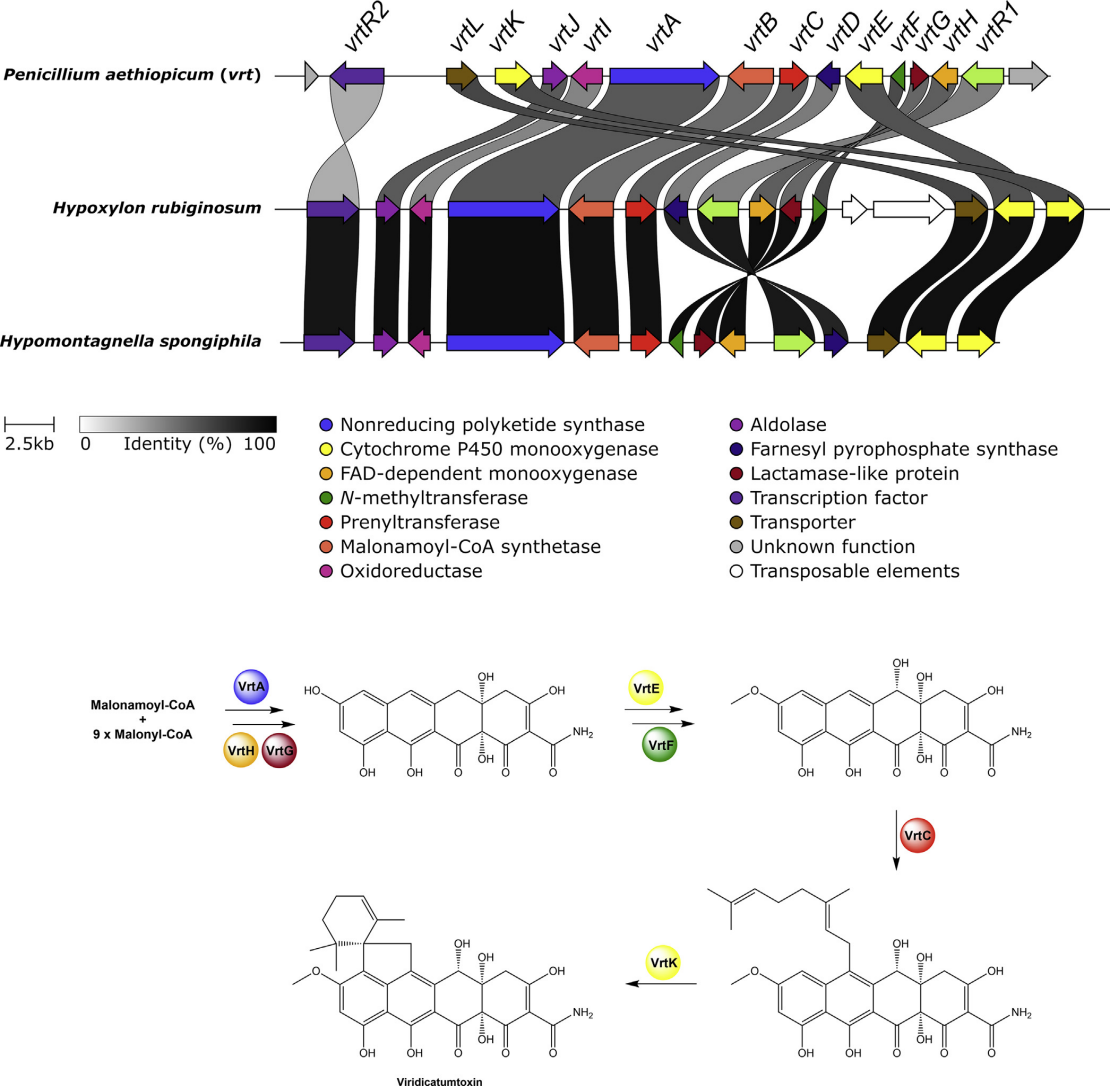
Synteny analysis of the viridicatumtoxin biosynthetic gene clusters from *Penicillium aethiopicum* (*vrt*), *Hypoxylon rubiginosum* and *Hypomontagnella spongiphila*. A biosynthetic scheme towards the formation of viridicatumtoxin is given below (in accordance with Matsuda & Abe 2016)

In addition to the meroterpenoid BGCs with unknown products in the *Hypoxylaceae*, the *H. rubiginosum* and *Hypom. spongiphila* genomes revealed the existence of a complete viridicatumtoxin BGC (*vrt*). This finding was surprising as viridicatumtoxin has not been reported from either species (and was not detected by us despite extensive screening efforts), and is so far only known from *Penicillium* and *Aspergillus* species (Frisvad *et al.* 2004, Drott *et al.* 2020). Beside some minor rearrangements of the genes the *vrt* BGCs of the *Hypoxylaceae* and *Penicillium aethiopicum* (Chooi *et al.* 2010) show high synteny (protein sequence similarity 60–70 %, Fig. 26). Interestingly, two additional genes were located in the *H. rubiginosum* BGC, one of which was similar to a retrotransposable element. These genes flank a locus inside the *vrt* BGC consisting of five genes which are inverted in comparison to the *vrt* BGC from *Hypom. spongiphila*. As the order of the remaining genes is identical between *H. rubiginosum* and *Hypom. spongiphila,* the transposable elements are potentially involved in cluster reorganisation. The identification of the *vrt* BGC outside the *Aspergillaceae* shows that the pathway might be more common within the *Ascomycota* than expected.

**Fig. 27:**
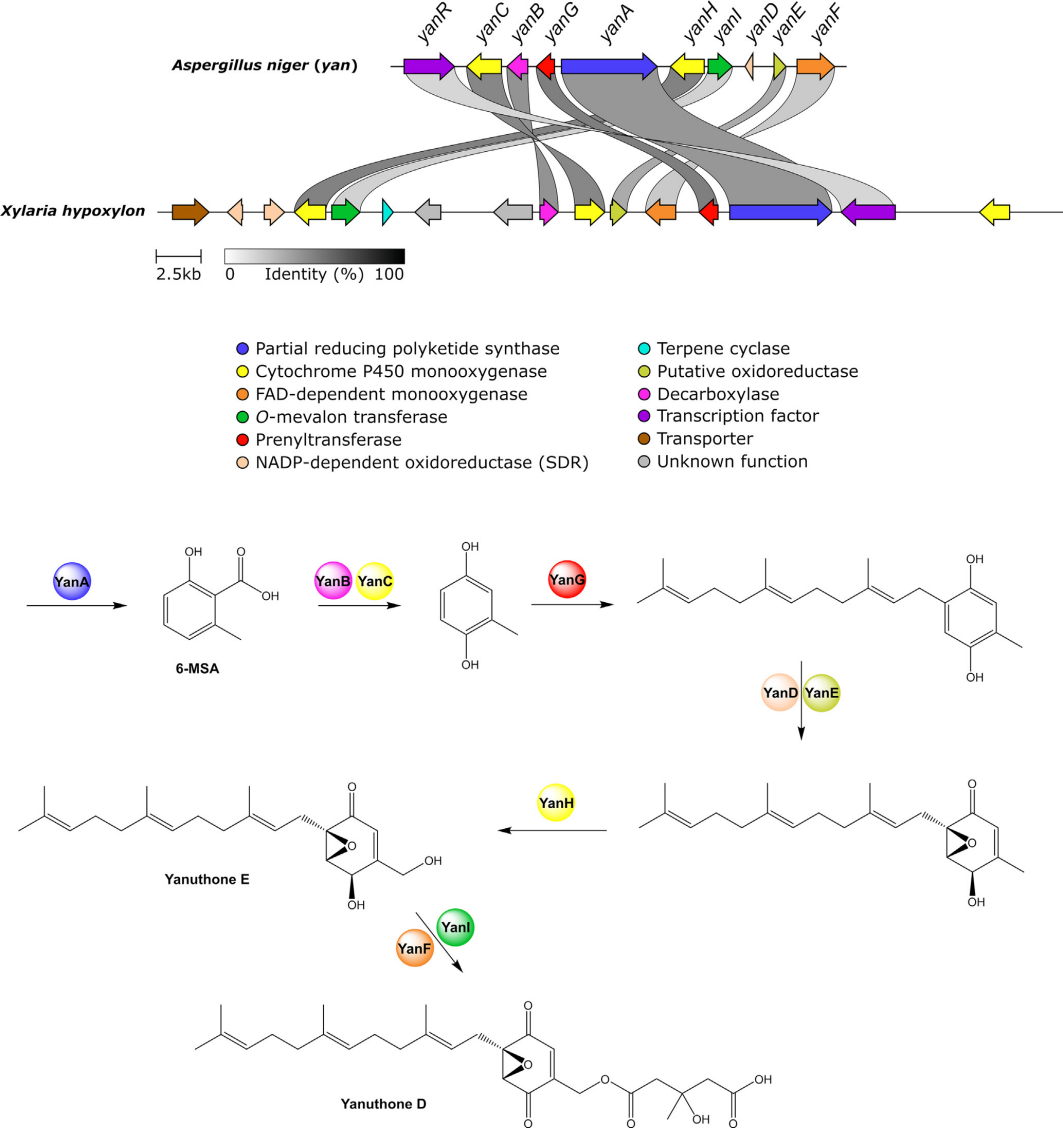
Synteny analysis of the yanuthone biosynthetic gene cluster (BGC) from *Aspergillus niger* and a homologous BGC from *Xylaria hypoxylon*, and proposed biosynthetic scheme for the formation of yanuthone D according to Matsuda and Abe (2016).

We also mined the genome of *X. hypoxylon* for meroterpenoid BGCs. A single BGC fulfilled the criteria for putative meroterpenoid production and Blastp analysis of the encoded proteins indicated similarity with the yanuthone BGC from *Aspergillus niger* (Holm *et al.* 2014)*.* A synteny analysis between these BGCs showed a conservation of all genes known to participate in yanuthone biosynthesis (Fig. 27). Interestingly, the *X. hypoxylon* BGC is slightly expanded by a terpene cyclase, oxidoreductase and P450 monooxygenase gene which potentially introduce further modifications to the yanuthone structure such as the cyclisation of the terpene chain. So far, there has been no report about meroterpenoids from *Xylaria* spp. and related genera, which could be correlated to this BGC leading to the assumption that the BGC product is at least new on family level.

In total, we located seven different putative meroterpenoid BGCs in the *Hypoxylaceae* and *X. hypoxylon* genomes, with only one appearing in more than one species. Except for the nodulisporic acid and *vrt* BGCs, the products of these pathways can only be partially or not predicted at all due to the lack of characterised homologous BGCs. The presence of a significant number of meroterpenoid BGCs in these organisms compared to the very few reports of related structures indicate that meroterpenoids might be more common than previously known. Consequently, investigation of these BGCs by ectopic or heterologous expression studies is a promising approach to reveal new natural product scaffolds.

#### Swainsonine

**Fig. 28:**
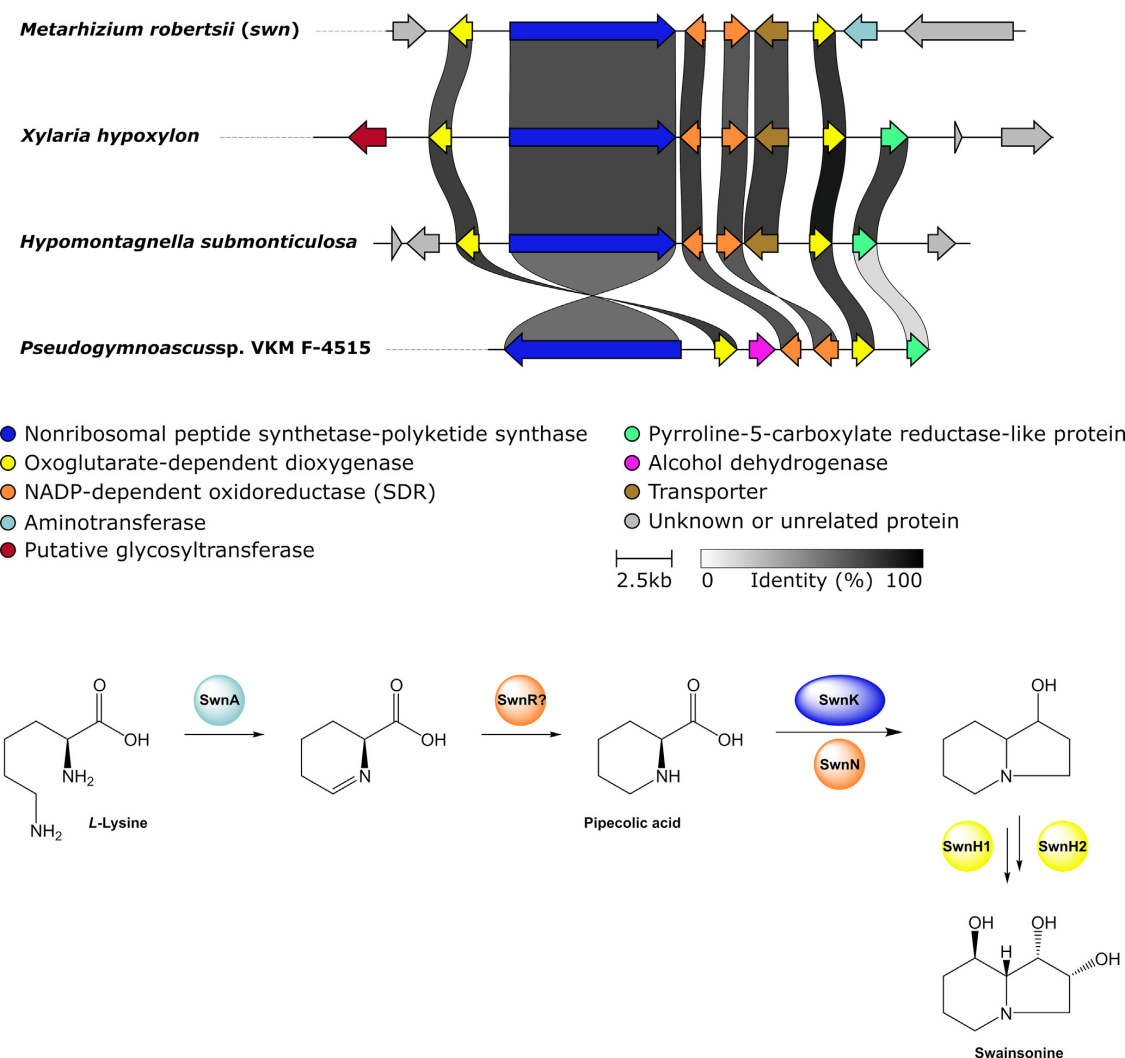
Synteny analysis of the swainsonine biosynthetic gene cluster (*swn*) known from *Metarhizium robertsii* and those identified from *Xylaria hypoxylon* and *Hypomontagnella submonticulosa*. A simplified biosynthetic scheme for the formation of swainsonine according to Luo *et al.* (2020) is given below.

One of the rarest types of fungal megasynthases is hybrid NRPS-PKS. These enzymes are known from bacteria and some fungi, and in the latter were associated with the production of tenuazonic acid and swainsonine (Yun *et al.* 2015, Cook *et al.* 2017, Theobald *et al.* 2019). Studies on the hybrid enzymes involved in tenuazonic acid (TAS1) and swainsonine (SwnK) biosynthesis also showed that the domain organisation of fungal NRPS-PKS can substantially vary with TAS1 and SwnK having a C-A-T-KS and A-T-KS-AT-KR-ACP-R domain architecture, respectively (Yun *et al.* 2015, Luo *et al.* 2020). During the genome mining for BGCs, these types of enzyme were also encountered in two species, namely *Hypom. submonticulosa* and *X. hypoxylon*. Both BGCs contained the same core set of genes known from the swainsonine pathway, thus a synteny analysis was carried out with the *swn* BGC from *Metarhizium robertsii* and *Pseudogymnoascus* sp. VKM F-4515 (Fig. 28) (Cook *et al.* 2017). The gene content and order was completely conserved between the BGCs from *M. robertsii*, *Hypom. submonticulosa* and *X. hypoxylon*, except for the lack of the aminotransferase gene *swnA* in the latter two species. SwnA was demonstrated to be involved in the transformation of lysine to pipecolic acid, the amino acid substrate of the NRPS-PKS SwnK. However, it is not necessarily required as alternative pathways in fungi can lead to pipecolic acid production (Luo *et al.* 2020).

The *X. hypoxylon* and *Hypom. submonticulosa* BGC share a pyrroline-5-carboxylate reductase-like (P5CR) gene that is absent from the *swn* BGC from *M. robertsii* but is present in the homologous BGC from *Pseudogymnoascus* sp. VKM F-4515 (Fig. 28). Screening of the culture crude extracts from *Hypom. monticulosa* and *X. hypoxylon* for swainsonine content did not indicate the presence of such molecules, but the P5CR-like protein might be involved in alteration of the structure preventing identification of the pathway product. Cblaster homology searches using the SwnK, SwnH1/2, SwnR and SwnN protein sequences from *X. hypoxylon* demonstrated that homologous BGCs are present in other *Xylaria* species and related fungi (*e.g. Rosellinia necatrix, X. grammica, X. multiplex*) indicating that the swainsonine-like BGC is common within the *Xylariaceae*. Cblaster analysis also identified similar unreported BGCs in a large range of fungi including *Clohesyomyces aquaticus* (*Pleosporales*), *Fusarium* sp. (*Hypocreales*), *Periconia macrospinosa* (*Pleosporales*), *Pseudovirgaria hyperparasitica* (*Capnodiales*), *Pyrenophora seminiperda* (*Pleosporales*), *Rhizodiscina lignyota* (*Dothideomycetes*), *Talaromyces rugulosus* (*Eurotiales*) and *Tothia fuscella* (*Microthyriales*).

The overall high cluster synteny between *M. robertsii* and *X. hypoxylon*/*Hypom. submonticulosa* prompted us to analyse the sequence similarity at the nucleotide level with a concatenated alignment (spanning the area between *swnH2 and swnH1* without non-coding regions) revealing that the clusters share around 73 % and 75 % nucleotide identity when comparing *M. robertsii* to *Hypom. submonticulosa* and *X. hypoxylon*, respectively. Such a high nucleotide identity suggests that this BGC is subjected to particular evolutionary mechanisms such as purifying selection or horizontal BGC transfer, which requires more detailed evolutionary analyses and additional genome sequences of each species.

Swainsonine is produced by various fungal species occupying different ecological niches including plant endophytes such as various *Alternaria* spp. and *Ipomoea carnea*. The compound is a potent cytotoxin that specifically targets the α-mannosidase II in the Golgi apparatus and therefore leads to toxicosis in animals feeding on infected plants (Cook *et al.* 2017). The occurrence of swainsonine BGCs in the *Xylariales* could putatively be correlated to the protection of the host plant against herbivores.

## Discussion

The genome mining of the analysed members of the *Hypoxylaceae* and *X. hypoxylon* revealed a high diversity of biosynthetic pathways that far outmatches the number of compounds known from the individual species (Helaly *et al.* 2018, Becker & Stadler 2021). We extensively screened the producing capabilities of these strains under various conditions including solid state fermentations, but only observed a restricted number of detectable compounds by HPLC-MS analysis (for selected results see ESI). In the case of *H. rickii* where we in addition performed large scale fermentation in a 70 L bioreactor only products of about 15 out of 55 predicted pathways were produced in detectable quantities (Kuhnert *et al.* 2015a,b, Surup *et al.* 2015, 2018a, Wiebach *et al.* 2016) indicating that the production of the majority of the compounds cannot be induced under laboratory conditions. Similar results were seen for *Hypom. montiulosa* which mainly produces sporothriolides and trienylfuranols under most conditions (Surup *et al.* 2014, Leman-Loubière *et al.* 2017, Tian *et al.* 2020) despite having more than 60 BGCs encoded.

The discrepancy between the number of predicted pathways in fungal genomes and the number of identified fungal compounds is explained by the regulation of BGC expression as the vast majority remain silent under laboratory conditions. It is a common hypothesis in the area of natural product research that activation of most of the pathways in fungi will likely depend on diverse, difficult to identify, environmental stimuli (Collemare & Seidl 2019, Keller 2019). *Hypoxylaceae* fungi usually exists as endophytes or saprotrophs (Stadler 2011), two life stages that occupy different ecological niches, which likely require the expression of different sets of genes. In these habitats *Hypoxylaceae* face diverse biotic and abiotic conditions that supposedly determine the production of secondary metabolites. We assume that some secondary metabolites play an important role in host-fungus interaction during the endophytic life stage, where they potentially modulate the host's immune response or serve as signalling molecules to induce host specific reactions. The chemical interaction of plant pathogenic fungi as well as mycorrhizal fungi with their plant hosts has been already studied to various degrees, but endophytic fungi have so far not been investigated in this context (Zeilinger *et al.* 2016).

Fungal secondary metabolites are also important for interspecific interactions. The microbiome of a tree is composed of a large number of different species, including mutualists and pathogens that are competing for space and resources (Witzell & Martín 2018). Schulz *et al.* (2019) postulated that this situation leads to a chemical warfare resulting into a balanced antagonism within the plant. The diversity of competitors could therefore require a broad spectrum of compounds to successfully defend the habitat. Alternatively, co-evolution of these diverse organisms may result in maintaining a large diversity of BGCs for very long time periods. Similarly, dead plant material is a highly competitive environment inhabited by various saprotrophic organisms (Hiscox & Boddy 2017). Under such conditions, the *Hypoxylaceae* have to deal with different organism, such as wood decaying basidiomycetes or insects. This is also the common environment for *Hypoxylaceae* to sexually reproduce. Production of secondary metabolites has been linked with reproduction and formation of survival structures (Calvo *et al.* 2002). The development of the stromata as well as survival within the dead wood therefore requires production of specific molecules for protection (Hiscox & Boddy 2017) which will to a certain extent supposedly differ from those formed during an endophytic lifestage. Consequently, it appears likely that the diversity of biosynthetic pathways in the *Hypoxylaceae* is linked to the requirements of sustaining two fundamentally different lifestyles.

In addition to the general lifestyle there is also the possibility that a high diversity of BGCs is connected to the distribution and host-specifity of a species (see Table S5 for details on the distribution and host preference of the analysed species). This means that species with a broader host range and widespread global occurrence are more likely to contain higher numbers of BGCs as they need to compete and communicate with a larger diversity of organisms. Our restricted dataset and the lack of knowledge about the preferred hosts for most tropical species (*i.e. H. lienhwacheense*, *H. rickii*, *P. hunteri*, *Hypomontagnella* spp.) prevents us to make hypotheses in this regard. However, there are supporting indicators for such a theory as for example *X. hypoxylon*, that occurs on a broad range of substrates and is frequently encountered in hardwood forests across Europe (and is verified from North America) (Peršoh *et al.* 2009), contained the highest number of BGCs whereas *H. fragiforme* with a similar distribution but high selectivity for *Fagus* spp. (Ju & Rogers 1996, Wendt *et al.* 2018) only encodes for half of the number of BGCs. In addition, the very rare species *H. lienhwacheense* (with only three confirmed records and unknown host preference), which has only been reported from South East Asia (Ju & Rogers 1996, Sir *et al.* 2019) is also indicative for a potential correlation as we only identified 24 BGCs, the by far lowest number in our dataset.

Comparison of the marine derived *Hypom. spongiphila* and terrestrial *Hypom. monticulosa* in terms of BGC composition showed little differences with only one unique BGC per species. This shows that in the case of *Hypomontagnella* adaptation to a marine environment does not rely on, or result in, the acquisition or loss of biosynthetic pathways. Our screening results did also not indicate any differences in secondary metabolite profiles between either species (Tian *et al.* 2020). It is likely that most of the metabolites produced in a terrestrial environment will not have any significance for the survival in an aquatic habitat.

The present study provides a general picture of the biosynthetic capabilities within various members of the *Hypoxylaceae* and *X. hypoxylon* including all known major biosynthetic families. In a recent large-scale genome mining study, BGCs of around 1 000 fungal genomes were identified by an automated pipeline showing that the classes *Eurotiomycetes* and *Sordariomycetes* encode among the largest numbers of BGCs on average per species (approx. 50) (Robey *et al.* 2021). Within the *Sordariomycetes* dataset (n = 198), only nine members of the *Xylariales* were included, with four of them belonging to the *Hypoxylaceae,* all of which were environmental isolates only tentatively identified to the genus level. In contrast, 128 genome sequences represented *Hypocreales* (in the cases of *Fusarium* and *Trichoderma* often with multiple strains per species), implying that the data are mainly representative for the *Hypocreales*. According to www.outlineoffungi.org (Wijayawardene 2020) the *Hypocreales* currently comprise around 5 000 described taxa, while there are around 3 500 known *Xylariales* species further highlighting that there is a huge genome coverage gap between these orders. Despite this fact, the average number of BGCs in the *Sordariomycetes* is highly similar to that which we observed for the *Hypoxylaceae,* indicating that a high diversity of BGCs was putatively inherited by ancestral lineages of the *Sordariomycetes*.

The number of herein studied *Hypoxylaceae* species still represents only a very small portion of the actual biodiversity within the family (< 4 %) and therefore the secondary metabolite biosynthetic diversity is likely to be much higher. This renders the *Hypoxylaceae* an exceptional target for the discovery of new natural products, biosynthetic pathways and biosynthesis-related enzymes. Furthermore, they are in general suitable for genetic manipulations as proven by various studies making respective investigations more feasible (Fang *et al.* 2012, Wang *et al.* 2018, Tian *et al.* 2020). We are currently exploiting this diversity of pathways and are analysing various BGCs with unusual genes or interesting products. In addition, we initiated sequencing of 50 further genomes to better cover the family for upcoming phylogenomic and biosynthesis studies.
