# Supplementary material for: Secondary metabolite biosynthetic diversity in the fungal family Hypoxylaceae and Xylaria hypoxylon
Source: Stud Mycol. 2021 Aug 26;99:100118. doi: 10.1016/j.simyco.2021.100118 (PMC8403587; doi:10.1016/j.simyco.2021.100118)
Supplement: Multimedia component 3 [file mmc3.pdf]

SUPPLEMENTARY INFORMATION

Secondary metabolite biosynthetic diversity in the fungal family *Hypoxylaceae* and *Xylaria hypoxylon*

Eric Kuhnert[1,*], Jorge C. Navarro-Muñoz[2], Kevin Becker[1,3], Marc Stadler[3], Jérôme Collemare[2], Russell J. Cox[1]

1 Centre of Biomolecular Drug Research (BMWZ), Institute for Organic Chemistry, Leibniz University Hannover, Schneiderberg 38, 30167, Hannover, Germany

2 Westerdijk Fungal Biodiversity Institute, Uppsalalaan 8, 3584 CT, Utrecht, The Netherlands

3 Department Microbial Drugs, Helmholtz Centre for Infection Research (HZI), and German Centre for Infection Research (DZIF), partner site Hannover-Braunschweig, Inhoffenstrasse 7, 38124 Braunschweig, Germany

*Correspondence: Eric Kuhnert, eric.kuhnert@oci.uni-hannover.de

Table of Contents

Supplementary tables (S1-S5) Page 2

Supplementary figures (S1-S16) Page 6

References Page 20

Table S1: Reference protein sequences used for phylogenetic reconstruction of Flavin-dependent monooxygenases involved in azaphilone biosynthesis.

Protein Acc. No.	protein name	organism	Reference
A6T923	FAD-dependent urate hydroxylase HpxO	*Klebsiella pneumoniae*	(O'Leary *et al.* 2009)
Q0D1P1	FAD-dependent monooxygenase TerC (terrein biosynthesis) - reannotated	*Aspergillus terreus*	(Zaehle *et al.* 2014)
A0A2U8U2L4	FAD-dependent monooxygenase AsL4 (xenovulene A biosynthesis)	*Sarocladium sp.*	(Schor *et al.* 2018)
A0A2U8U2L0	FAD-dependent monooxygenase AsL6 (xenovulene A biosynthesis)	*Sarocladium sp.*	(Schor *et al.* 2018)
A0A1Y0BRF9	FAD-dependent monooxygenase AdrH (andrastin biosynthesis)	*Penicillium roqueforti*	(Rojas-Aedo *et al.* 2017)
Q4WLD1	FAD-dependent monooxygenase Pyr5 (pyripyropene biosynthesis)	*Aspergillus fumigatus*	(Itoh *et al.* 2010)
Q0C9L4	FAD-dependent monooxygenase CtvC (citreoviridin biosynthesis)	*Aspergillus terreus*	(Lin *et al.* 2016)
H1VM35	FAD-dependent monooxygenase DpchE (higginsianin biosynthesis)	*Colletotrichum higginsianum*	(Tsukada *et al.* 2020)
A0A3B1EFQ2	FAD-dependent monooxygenase Str9 (strobilurin A biosynthesis)	*Strobilurus tenacellus*	(Nofiani *et al.* 2018)
Q0CJ62	6-methylsalicylic acid decarboxylase AtA (terreic acid biosynthesis)	*Aspergillus terreus*	(Guo *et al.* 2014)
n/a	FAD-dependent monooxygenase MrPigN (azaphilone biosynthesis)	*Monascus ruber*	(Chen *et al.* 2017)
G3XMC2	FAD-dependent monooxygenase AzaH (azanigerone biosynthesis)	*Aspergillus niger*	(Zabala *et al.* 2012)
A2QTE7	FAD-dependent monooxygenase Orf3 (pestalamide biosynthesis)	*Aspergillus niger*	(Wang *et al.* 2018)
QNC49734	FAD-dependent monooxygenase Hfaza1D (azaphilone biosynthesis)	*Hypoxylon fragiforme*	(Becker *et al.* 2021)
B6HN76	FAD-dependent monooxygenase SorC (sorbicillinoid biosynthesis)	*Penicillium rubens*	(Kahlert *et al.* 2020)
G0R6T0	FAD-dependent monooxygenase Sor5 (sorbicillinoid biosynthesis)	*Trichoderma reesei*	(Derntl *et al.* 2017)
Q5B8A3	FAD-dependent monooxygenase PkfD (aspernidine A biosynthesis)	*Aspergillus nidulans*	(Yaegashi *et al.* 2013)
A0A084B9Z5	FAD-dependent monooxygenase SAT7 (satratoxin biosynthesis)	*Stachybotrys chartarum*	(Semeiks *et al.* 2014)
Q5BEJ7	FAD-dependent monooxygenase AfoD (asperfuranone biosynthesis)	*Aspergillus nidulans*	(Chiang *et al.* 2009)
n/a	FAD-dependent monooxygenase CazL (chaetoviridin biosynthesis)	*Chaetomium globosum*	(Winter *et al.* 2012)
A0A4V1E8I5	FAD-dependent monooxygenase EupB (eupenifeldin biosynthesis)	*Phoma sp.*	(Zhai *et al.* 2019)
A0A2U8U2L6	Alicylate hydroxylase AsL1 (xenovulene A biosynthesis)	*Sarocladium sp.*	(Schor *et al.* 2018)
Q5AUX8	FAD-dependent monooxygenase DbaH (Derivative of benzaldehyde biosynthesis)	*Aspergillus nidulans*	(Gerke *et al.* 2012)

B8M9J8	FAD-dependent monooxygenase TropB (tropolone biosynthesis)	*Talaromyces stipitatus*	(Davison *et al.* 2012)
QNC49728	FAD-dependent monooxygenase Hfaza2D (azaphilone biosynthesis)	*Hypoxylon fragiforme*	(Becker *et al.* 2021)

Table S2: Reference protein sequences used for phylogenetic reconstruction of NRPS-like proteins with A-T-TE.

Protein Acc. No.	protein name	organism	Reference
B8NTZ9	Piperazines biosynthesis cluster protein A LnaA	*Aspergillus flavus*	(Forseth *et al.* 2013)
Q0CU19	Butyrolactone IIa synthetase BtyA	*Aspergillus terreus*	(Hühner *et al.* 2018)
Q5B7T4	Microperfuranone synthase MicA	*Aspergillus nidulans*	(Yeh *et al.* 2012)
B7STY1	Atromentin synthetase AtrA	*Tapinella panuoides*	(Schneider *et al.* 2008)
I6NXV7	Atromentin synthetase GreA	*Suillus grevillei*	(Wackler *et al.* 2012)
A0A0S1RUN4	Atromentin synthetase InvA2	*Paxillus involutus*	(Braesel *et al.* 2015)
A0A0S2E7Z1	Atromentin synthetase InvA1	*Paxillus involutus*	(Braesel *et al.* 2015)
A7XRY0	Didemethylasterriquinone D synthetase TdiA	*Aspergillus nidulans*	(Balibar *et al.* 2007)
AUO29226	Phenguignaric acid synthetase PgnA	*Aspergillus terreus*	(Hühner *et al.* 2018)
Q0CWD0	Aspulvinone E synthase ApvA	*Aspergillus terreus*	(Hühner *et al.* 2018)
A0A336U965	Aspulvinone E synthetase MelA	*Aspergillus terreus*	(Geib *et al.* 2016)

Table S3: Composition of growth media used in the secondary metabolite screening.

Name	Ingredient	Conc. [g/L]	Notes/instructions
SMYA (semi-viscous)	Maltose<br>Yeast extract<br>Meat peptone<br>agar	40.0<br>10.0<br>10.0<br>4.0	-
YMG (liquid)	Malt extract<br>D-glucose<br>Yeast extract	10.0<br>4.0<br>4.0	pH 6.3
YMG + beech chips	Beech chips	4.0	-
CYG10 (liquid)	Corn meal<br>D-glucose	50.0<br>10.0	-

	Yeast extract	1.0	
GG1 (liquid)	Glycerol<br>D-glucose<br>Yeast autolysate<br>Soybean meal<br>Tomato paste<br>Sodium citrate<br>NH_4SO_4	75.0<br>10.0<br>5.0<br>5.0<br>5.0<br>2.0<br>2.0	pH 7.5
GZ (liquid)	Oat meal<br>Corn steep liquor (liq.)<br>Soybean meal	30.0<br>10.0<br>10.0	pH 6.5
MMK2 (liquid)	Mannitol<br>Yeast extract<br>Murashoge & Skoop salts	40.0<br>5.0<br>4.3	-
MOG (liquid)	Mannitol<br>MES<br>Oat flour<br>Yeast extract<br>L-glutamic acid	75.0<br>16.2<br>15.0<br>5.0<br>4.0	pH 6.0
Supermalt (liquid)	Malt extract<br>Yeast extract<br>$FeSO_4 \times 7\ H_2O$<br>$ZnSO_4 \times 7\ H_2O$	50.0<br>10.0<br>0.02<br>0.007	-
BRFT (rice, solid)	Yeast extract<br>Sodium tartrate × 2 H_2O<br>KH_2PO_4	1.0<br>0.5<br>0.5	12 mL “base liquid” solution added to 3.3 g of brown rice
Vermiculite + YES (solid)	Sucrose<br>Yeast extract<br>$MgSO_4 \times 7\ H_2O$<br>$ZnSO_4 \times 7\ H_2O$<br>$CuSO_4 \times 7\ H_2O$	150.0<br>20.0<br>0.5<br>0.0001<br>0.001	12 mL YES added to 34 ccm vermiculite (>0.5 cm diam.)
Wheat (solid)	Disodium tartrate × 2 H_2O<br>Glycerol<br>Yeast extract<br>KH_2PO_4<br>$MgSO_4 \times 7\ H_2O$<br>$FeSO_4 \times 7\ H_2O$	10.0<br>2.0<br>2.0<br>1.0<br>1.0<br>0.5	8.25 mL “base liquid” added to 5 g of whole wheat grains

Tab. S4: Blastp analysis for the proteins encoded in the ergot alkaloid gene cluster of *Hypomontagnella monticulosa*, *Hypom. spongiphila* and *Annulohypoxylon truncatum*. The closest hit and its respective query coverage (QC) and identity (Ident) are listed.

Gene name	Closest blastp hit	QC [%]	Ident [%]
Hypomontagnella monticulosa			
easF	easF [*Trichophyton benhamiae* CBS 112371], D4AK46.1	96	63.7
easE	easE [*Epichloe festucae* var. *lolii*], A2TBU3.1	97	53.1
DMATS	DMATS [*Epichloe coenophiala*], Q6X2E2.1	99	64.8
easG	easG [*Epichloe festucae* var. *lolii*], A2TBU1.1	98	67.8
easA	easA [*Epichloe festucae* var. *lolii*], A2TBU0.1	97	71.1
easD	easD [*Aspergillus fumigatus*], D3J0Z1.1	100	72.0
easC	easC [*Aspergillus fumigatus* Af293], Q4WZ63.1	94	67.4
Hypomontagnella spongiphila			
easF	easF [*Microsporum canis* CBS 113480], C5FTN1.1	96	64.8
easE	easE [*Epichloe festucae* var. *lolii*], A2TBU3.1	97	52.5
DMATS	DMATS [*Epichloe coenophiala*], Q6X2E2.1	95	67.1

easG	easG [*Epichloe festucae* var. *lolii*], A2TBU1.1	98	66.7
easA	easA [*Epichloe festucae* var. *lolii*], A2TBU0.1	97	71.1
easD	easD [*Aspergillus fumigatus*], D3J0Z1.1	86	71.5
easC	easC [*Aspergillus fumigatus* Af293], Q4WZ63.1	94	69.7
Annulohypoxylon truncatum			
lpsA	lpsA [*Epichloe festucae* var. *lolii*], Q96V34.2	99	64.7
easH	easH [*Claviceps purpurea* 20.1], G8GV69.1	96	62.0
easF	easF [*Trichophyton benhamiae* CBS 112371], D4AK46.1	95	66.8
DMATS	DMATS [*Epichloe coenophiala*], Q6X2E2.1	94	64.1
easE	easE [*Epichloe festucae* var. *lolii*], A2TBU3.1	97	55.7
cloA	cloA [*Claviceps purpurea*], Q2PBY6.1	100	46.2
easG	easG [*Epichloe festucae* var. *lolii*], A2TBU1.1	99	63.8
easD	easD [*Penicillium roqueforti* FM164], W6QIM3.1	84	67.0
easA	easA [*Epichloe festucae* var. *lolii*], A2TBU0.1	97	67.9
lpsB	lpsB [*Epichloe festucae* var. *lolii*], A2TBU4.1	97	65.8
easC	easC [*Aspergillus fumigatus* Af293], Q4WZ63.1	96	69.0

Tab. S5: Distribution and host preferences of the analyzed *Hypoxylaceae* species and *Xylaria hypoxylon*.

**Species**	**Distribution**	**Host preference**
Annulohypoxylon truncatum	Common in Southern USA, known from Mexico	Probably *Quercus* spp.
Daldinia concentrica	very common across most European countries	Prefers *Fraxinus* but also occurs on other substrates (*Alnus* spp., *Populus* spp., *Betula alba*, *Quercus pubescens*, *Acacia cyanophylla*, *Ulmus minor*, *Carpinus* spp., *Acer* spp., *Fagus sylvatica*, *Prunus spinosa*)
Hypomontagnella monticulosa	Very common in the tropics	unknown
Hypomontagnella spongiphila	Only one record	sponge
Hypomontagnella submonticulosa	Common in the USA	unknown
Hypoxylon fragiforme	common in Europe and North America	Host specific to *Fagus* spp.
Hypoxylon lienhwacheense	Rare, only known from South-East China and Thiland	unknown
Hypoxylon pulicicidum	supposedly common in the tropics	unknown
Hypoxylon rickii	Known from Argentina, Brasil, Mexico, Caribbean	unknown
Hypoxylon rubiginosum	Common across Europe	Prefers *Fraxinus* spp. but also occurs on other substrates (*Fagus sylvatica*, *Ulmus* spp., *Populus tremula*)
Jackrogersella multiformis	common in Europe and North America	Host specific to *Betula* spp.
Pyrenopolyporus hunteri	known from various tropical countries	unknown
Xylaria hypoxylon	very common in Europe, known from the USA	No host preference, known from *Fagus sylvatica, Fraxinus excelsior, Carpinus betulus, Quercus* spp., *Populus tremula, Corylus avellana, Tilia cordata, Acer campestre*, *Picea abies, Salix* spp., *Lonicera xylosteum*, etc.

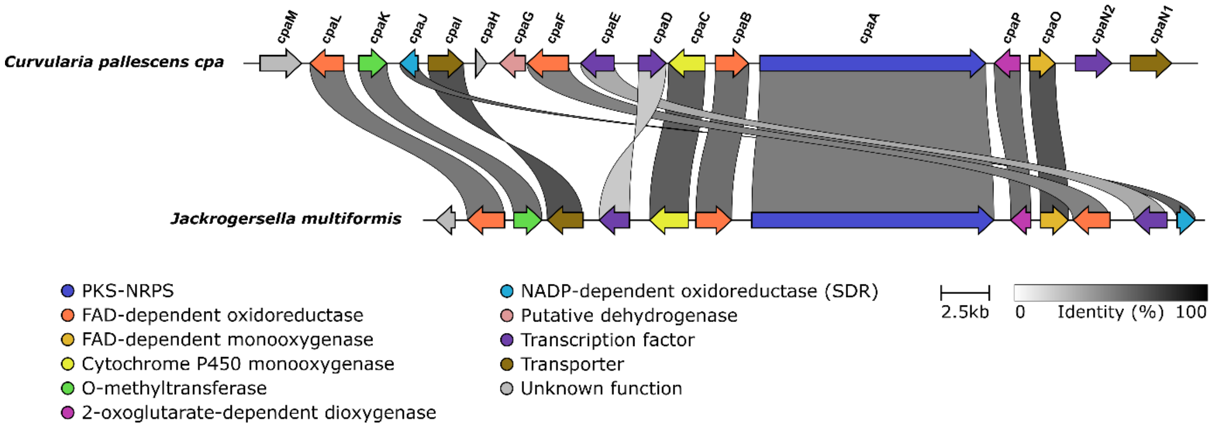

Fig. S1: Synteny analysis between the curvupallide biosynthetic gene cluster (*cpa*) of *Curvularia pallescens* and a homologous BGC from *Jackrogersella multiformis* visualized with clinker.

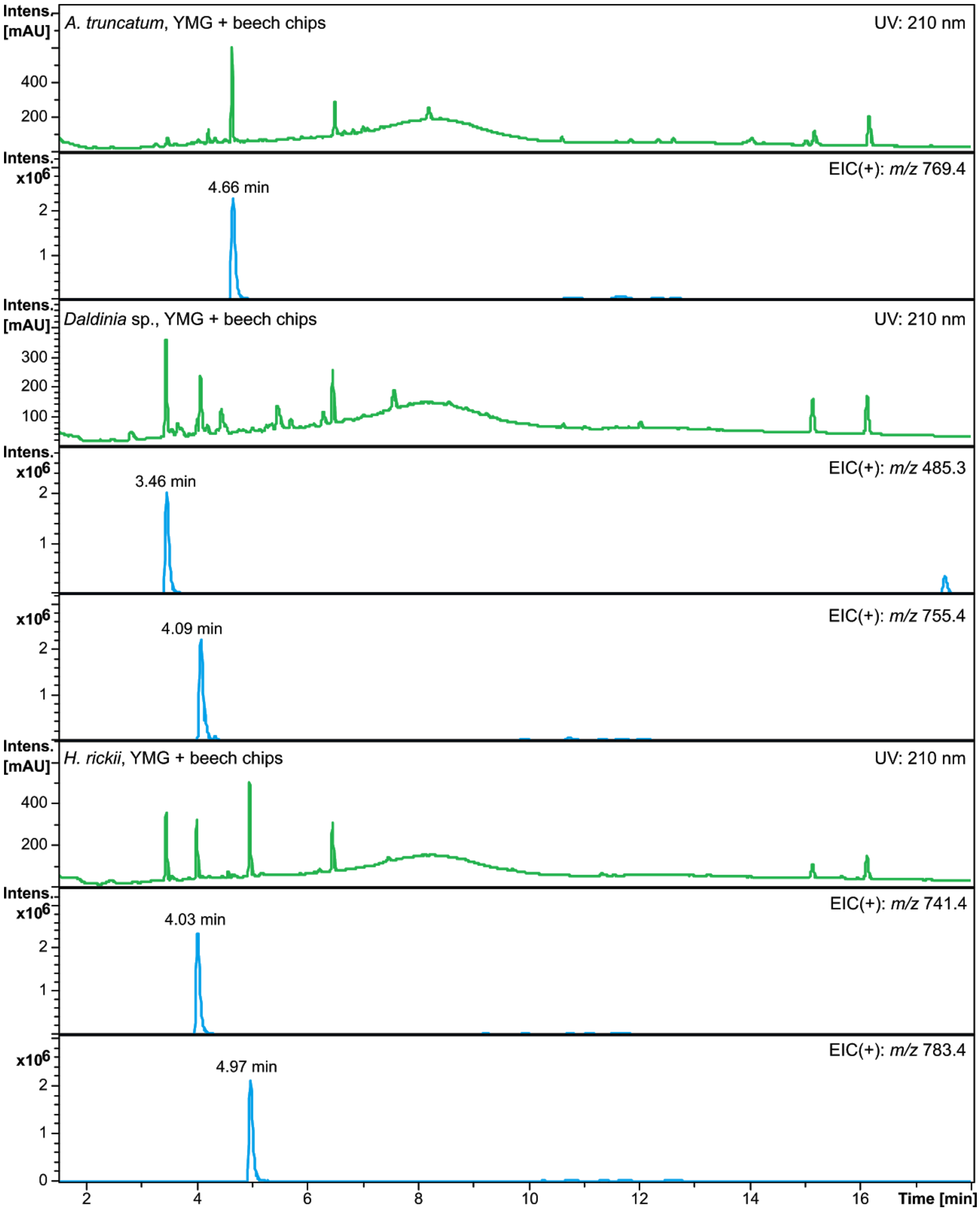

Fig. S2: HPLC-UV chromatograms of culture-derived extracts of *Annulohypoxylon truncatum*, *Daldinia sp.*, and *Hypoxylon fragiforme* (top panel) and MS spectra of peaks identified as siderophores (bottom, next page). Top: green traces, UV chromatograms; blue traces, extracted ion chromatograms (EICs) of *m/z* 769.4, 485.3, 755.4, 741.4, and 783.4, representing the $[M+H]^+$ ions corresponding to coprogen (MW 768.4), dimerumic acid (MW 484.3 Da), N^{α}-dimethylcoprogen B (MW 754.4), N^{α}-methylcoprogen B (MW 740.4 Da), and N^{α}-methylcoprogen (MW 782.4), respectively. Bottom: MS spectra of the respective siderophores .

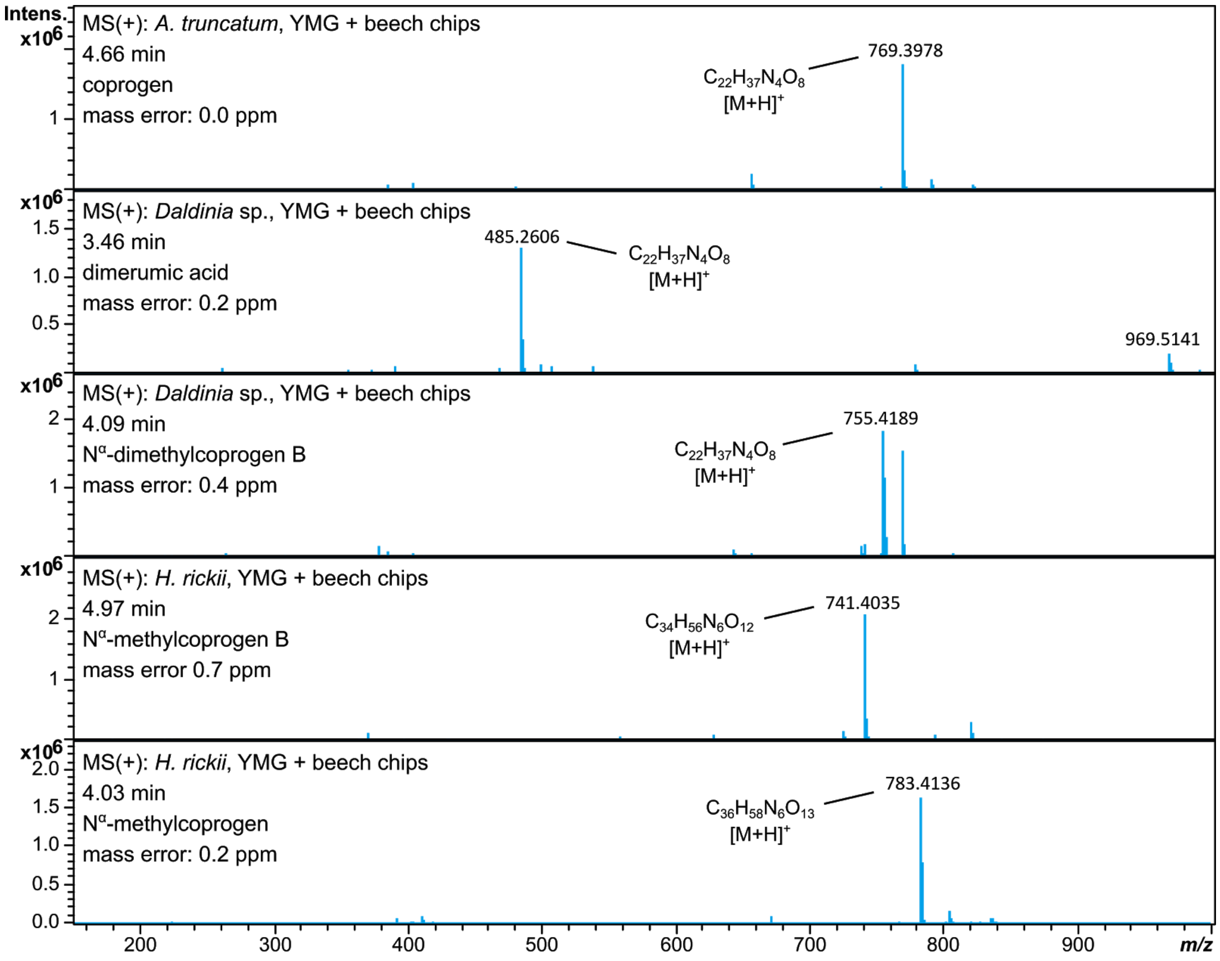

Fig. S2 (continued)

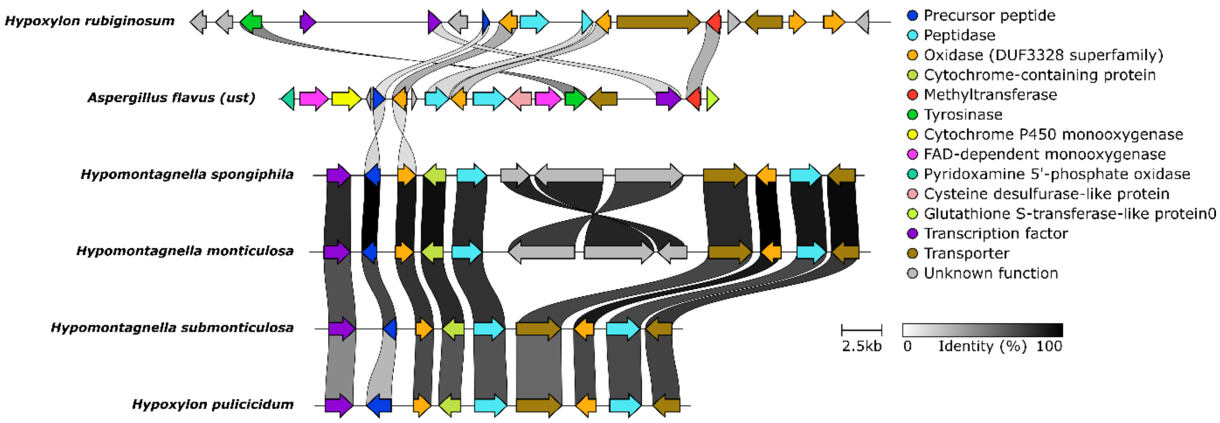

Fig. S3: Synteny analysis between RiPP biosynthetic gene cluster identified in the *Hypoxylaceae* and the ustiloxin B cluster (ust) from *A. flavus* visualized by the clinker tool.

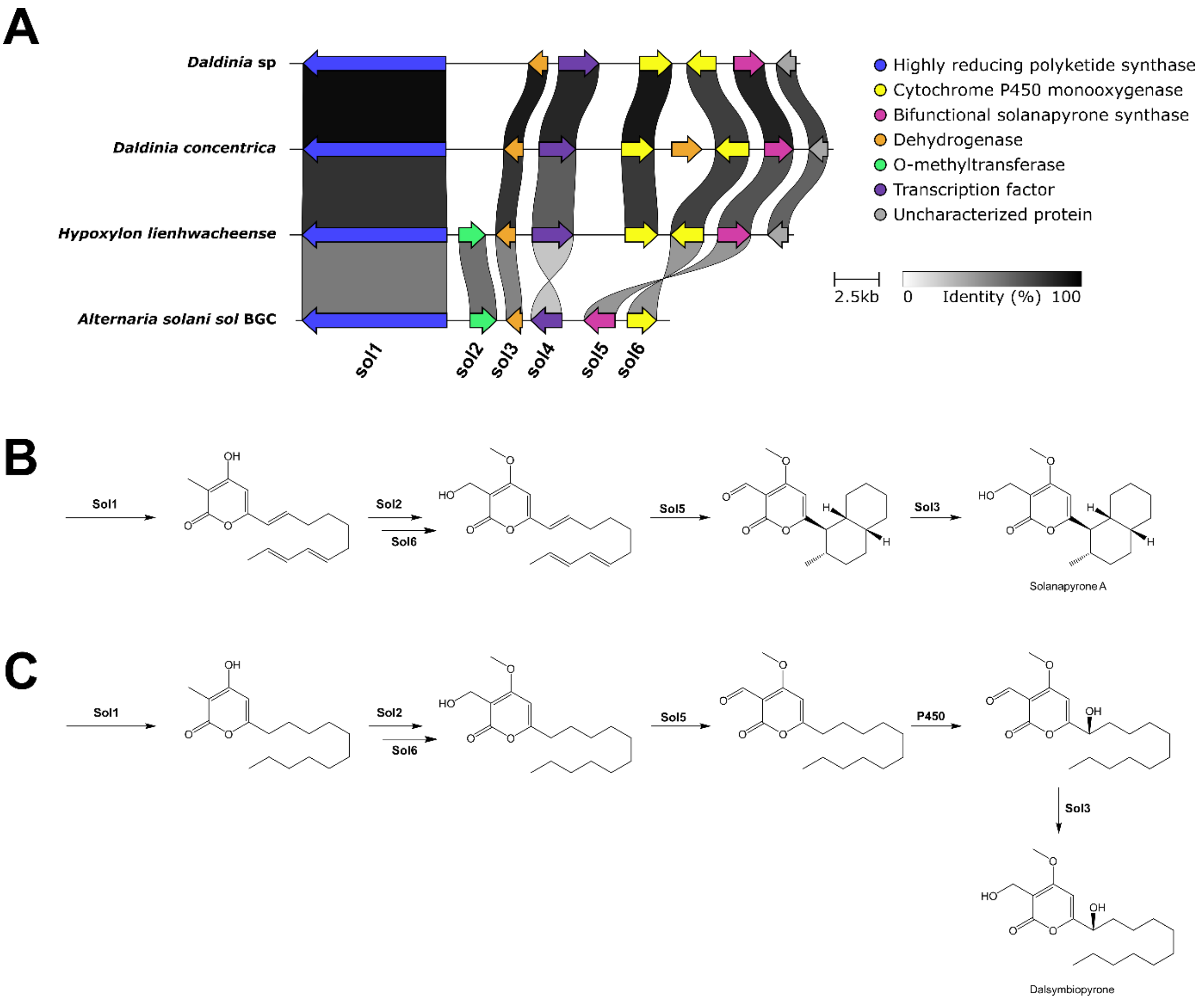

Fig. S4: A; Synteny analysis between the solanapyrone biosynthetic gene cluster (*sol* BGC) and related BGCs from the *Hypoxylaceae* visualized with clinker. B, C; Biosynthetic scheme for solanapyrone A according to literature is shown and a putative pathway for the biosynthesis of dalsymbiopyrone by *Hypoxylaceae* species is predicted. BGCs lacking a methyltransferase are predicted to produce a demethylated analog of dalsymbiopyrone.

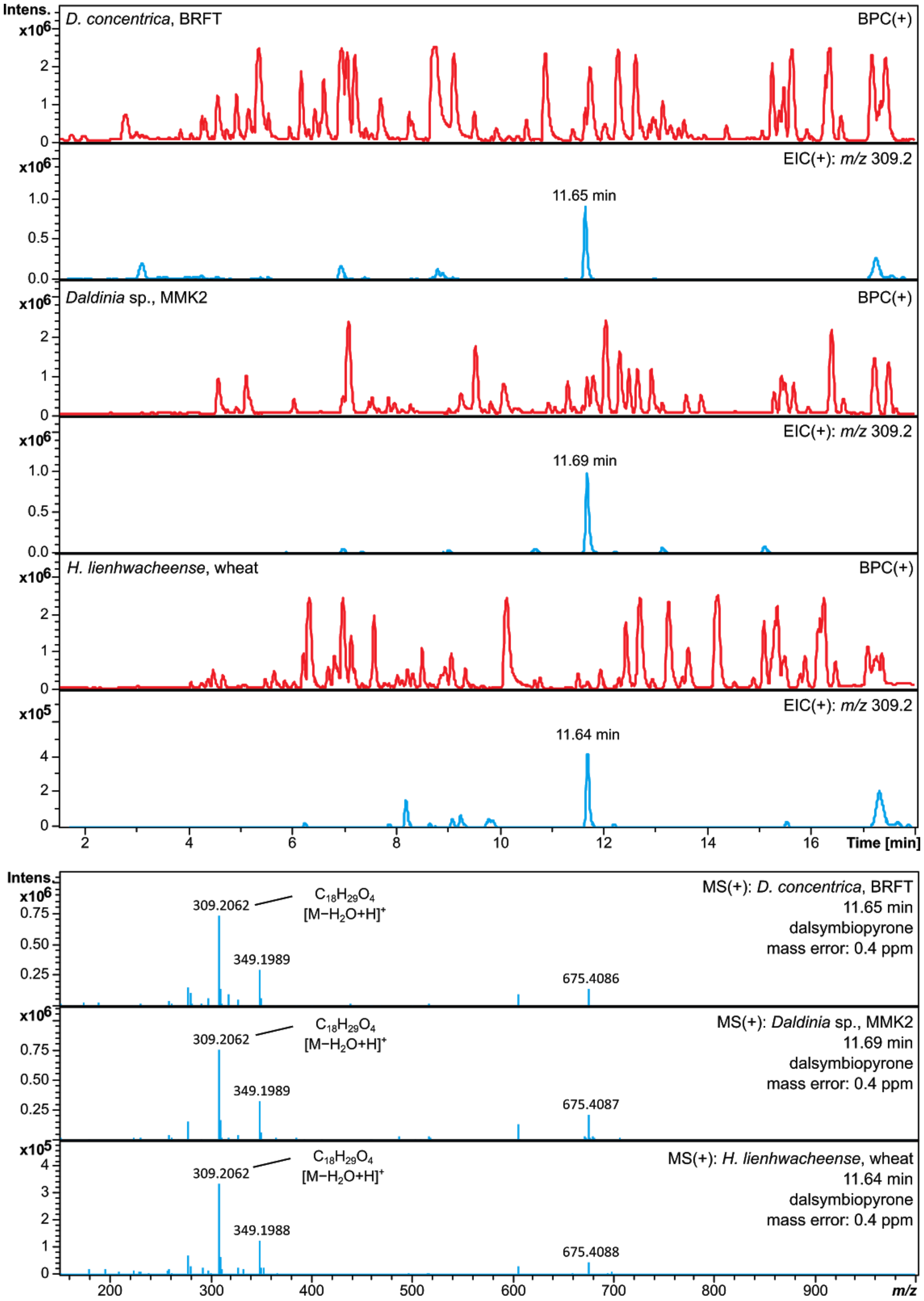

Fig. S5: HPLC-MS chromatograms of culture-derived extracts of *Daldinia concentrica*, *Entonaema liquescens*, and *Hypoxylon lienhwacheense* (top panel) and MS spectra of dalsymbiopyrone (bottom). Top: red traces, positive ion mode base peak chromatograms [BPC(+)]; blue traces, extracted ion chromatograms (EICs) of *m/z* 309.2, representing the $[M{-}H_2O{+}H]^+$ ion corresponding to dalsymbiopyrone (MW 326.2 Da). Bottom: MS spectra of dalsymbiopyrone.

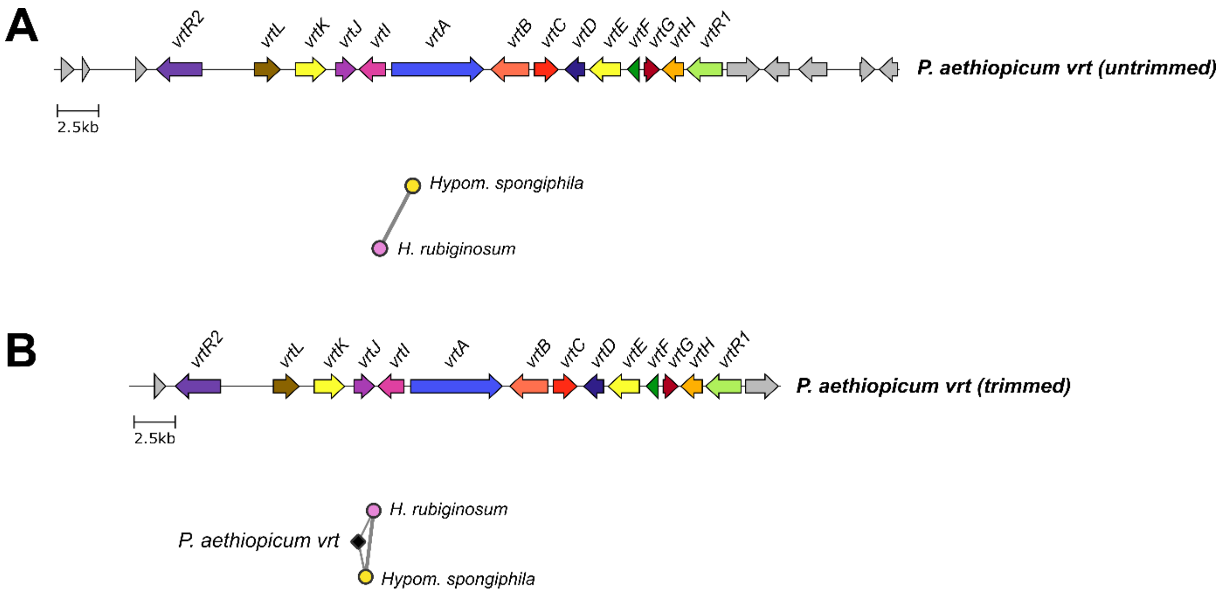

Fig. S6: Impact of uncurated reference biosynthetic gene cluster (BGC) on subnetwork formation during BiG-SCAPE analysis. A; uncurated BGC information of the viridicatumtoxin BGC (*vrt*) from *Penicillium aethiopicum* retrieved from the MIBiG repository prevents BiG-SCAPE subnetwork formation with homologous BGCs from the *Hypoxylaceae* genomes under global mode settings and a cutoff value of 0.4. B; Manual curation (trimming) of the *vrt* BGC results in subnetwork formation of the three homologous viridicatumtoxin BGCs.

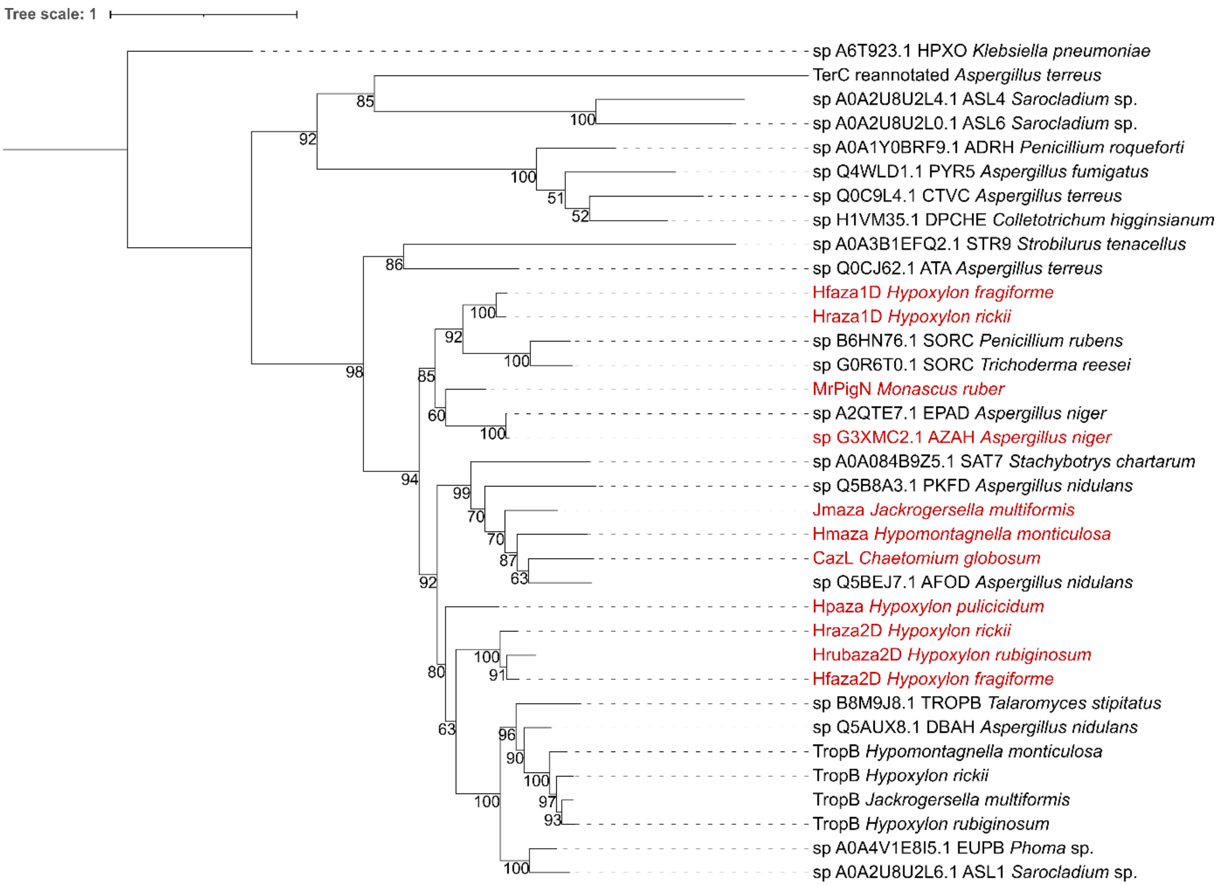

Fig. S7: Maximum-Likelihood phylogenetic analysis of FAD-dependent monooxygenases known from azaphilone biosynthetic pathways (red) and other characterized FAD-dependent monooxygenases (black). Tree is rooted with the FAD-dependent urate hydroxylase (HPXO) from *Klebsiella pneumoniae*. Branch support was determined with the ultrafast bootstrap approximation. Support values [%] above 50 % are indicated. Only support values above 95 % are deemed significant. Available GenBank accession numbers are given.

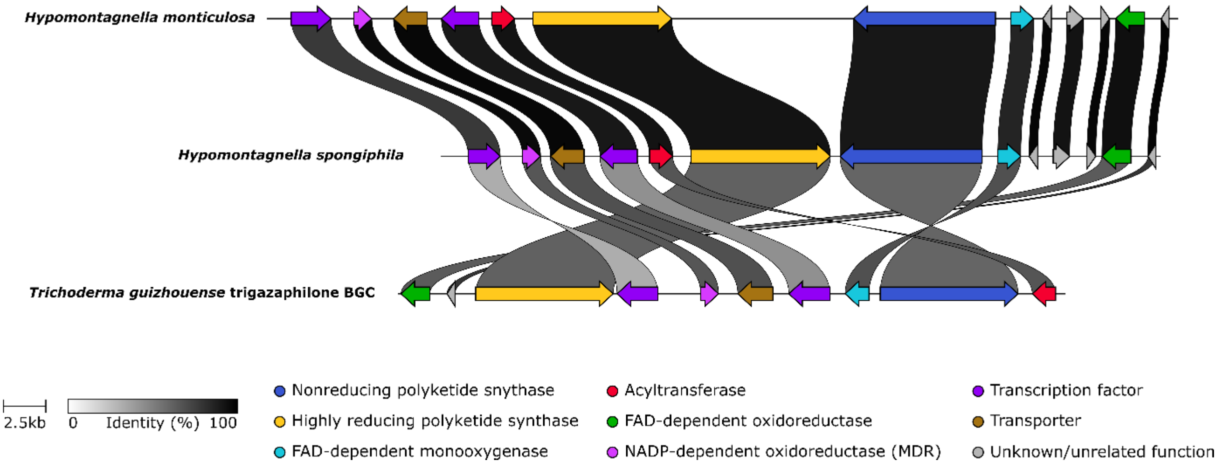

Fig. S8: Synteny analysis between the trigazaphilone biosynthetic gene cluster from *Trichoderma guizhouense* and its homologs from *Hypomontagnella monticulosa* and *Hypom. spongipila* visualized by the clinker tool.

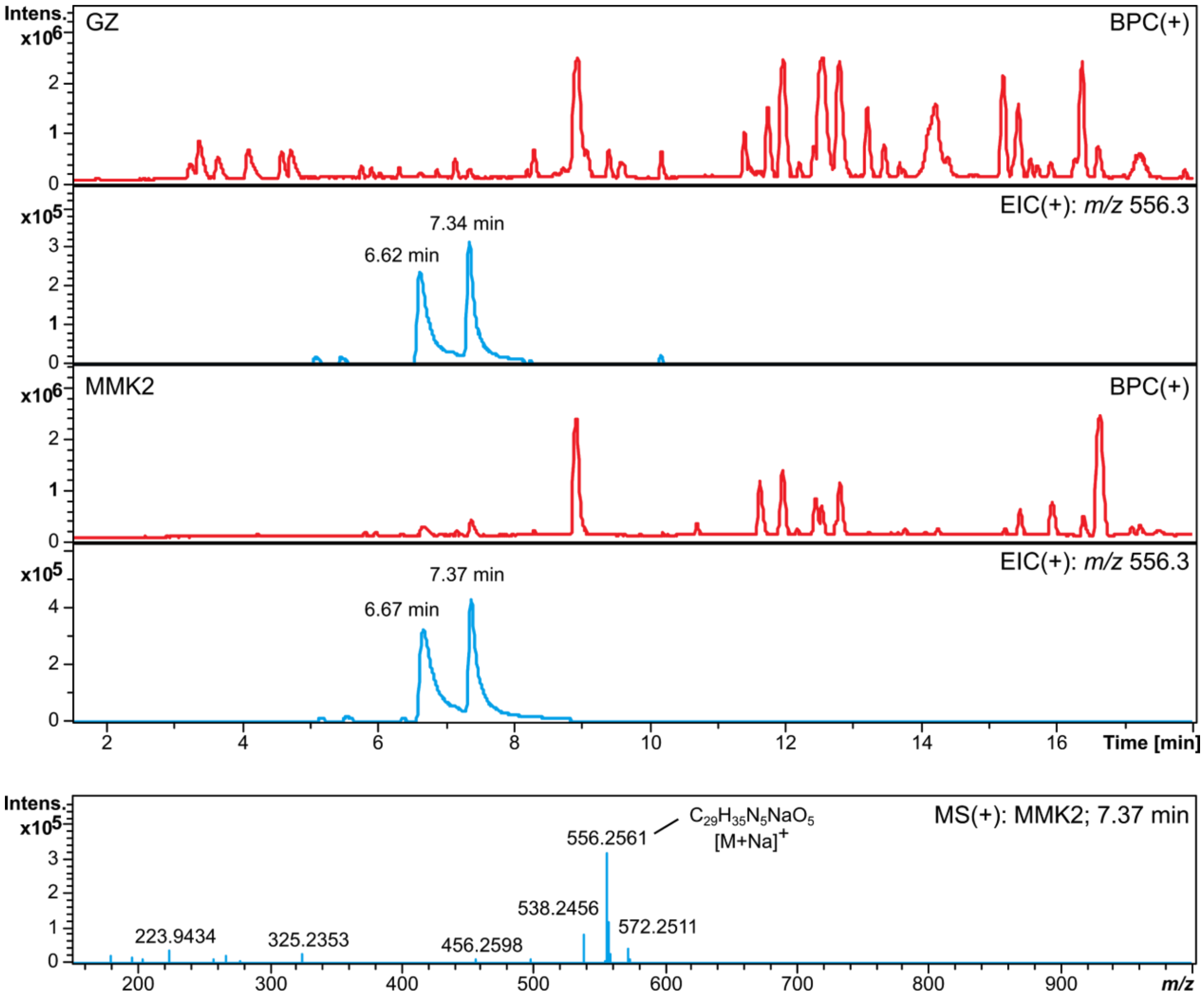

Fig. S9: HPLC-MS chromatograms of culture-derived extracts of *Annulohypoxylon truncatum* (top panel) and representative MS spectrum of a peak tentatively identified as ergovaline (bottom). Top: red traces, positive ion mode base peak chromatograms [BPC(+)]; blue traces, extracted ion chromatograms [EIC(+)] of *m/z* 556.3, representing the $[M+Na]^+$ ion corresponding to ergovaline (MW 533.3 Da). Bottom: representative MS spectrum of ergovaline (tentatively identified, ion formula accuracy: 5.5 ppm).

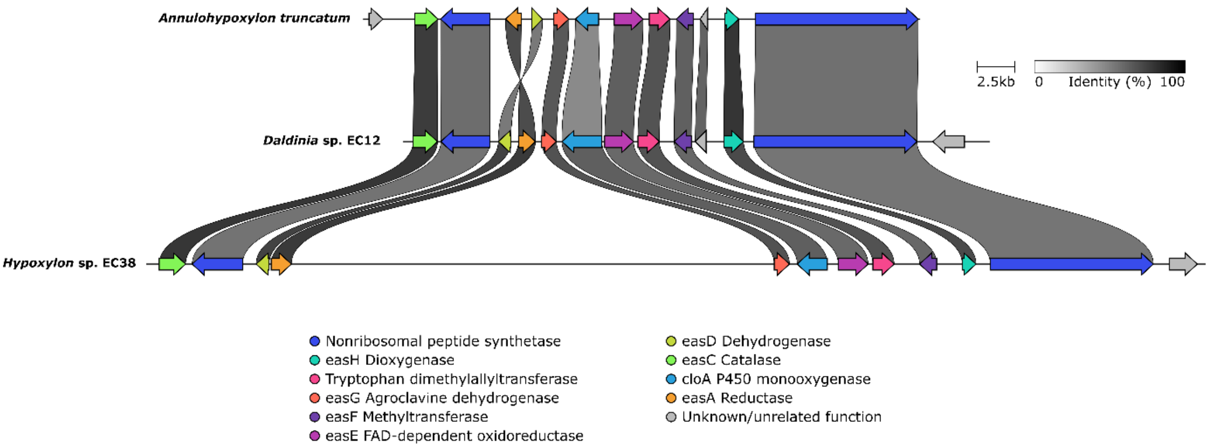

Fig. S10: Synteny analysis between the ergopeptine biosynthetic gene cluster identified from *A. truncatum*, *Hypoxylon* sp. EC38 and *Daldinia* sp. EC12 visualized by the clinker tool.

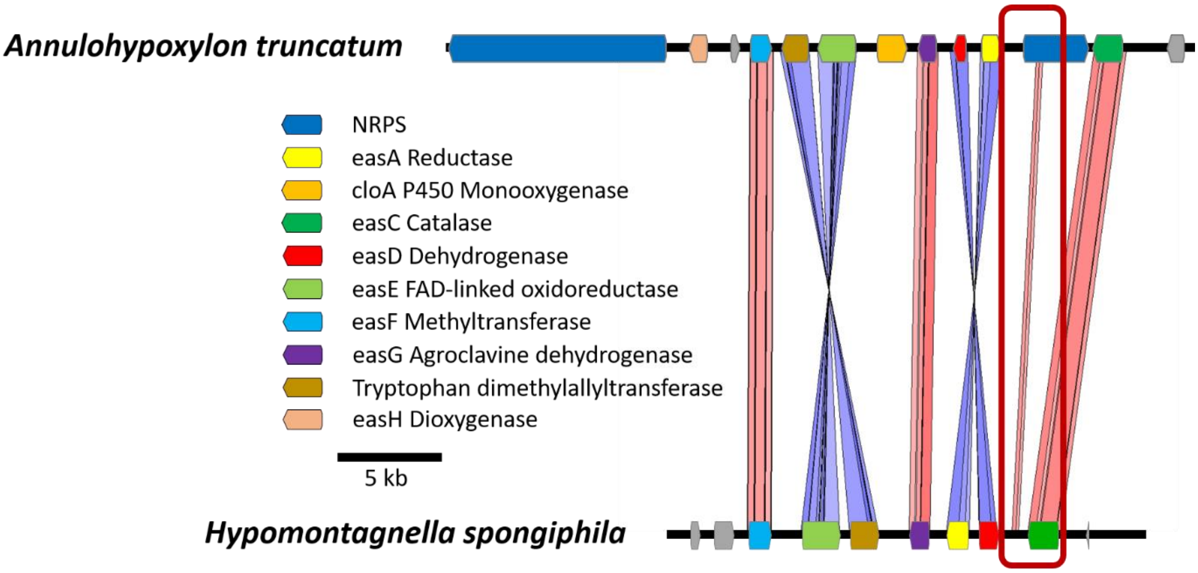

Fig. S11: Homology analysis between the ergot alkaloid biosynthetic gene cluster from *A. truncatum* and *Hypom. spongiphila* performed with the Artemis Comparison Tool (ACT). The tblastx algorithm identified the remainders of a NRPS gene in *Hypom. spongiphila* highlighted by the red frame.

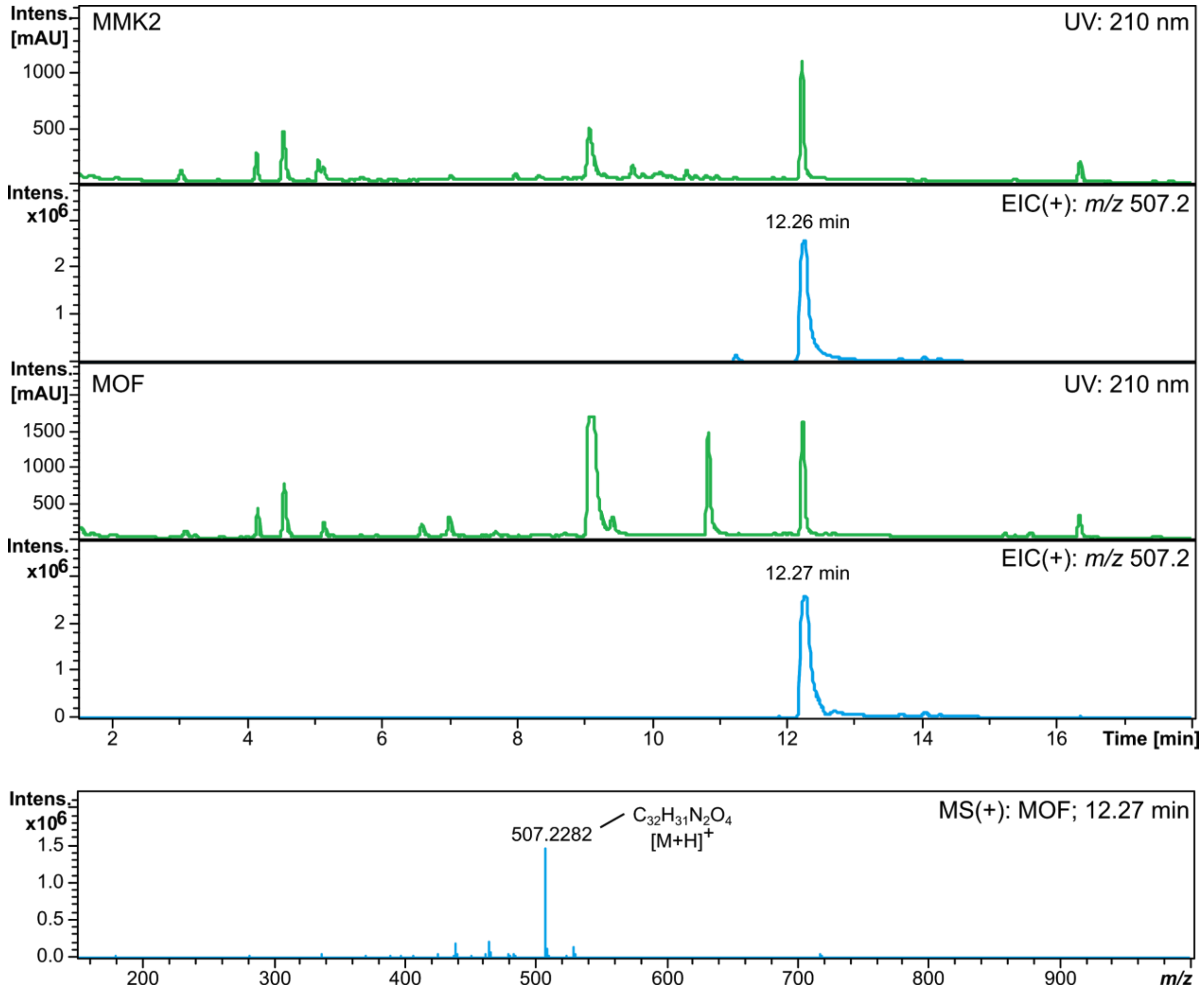

Fig. S12: HPLC-MS chromatograms of culture-derived extracts of *Daldinia concentrica* (top panel) and representative MS spectrum of cochliodinol (bottom). Top: green traces, UV chromatograms; blue traces, positive ion mode extracted ion chromatograms [EIC(+)] of *m/z* 507.2, representing the $[M+H]^+$ ion corresponding to cochliodinol (MW 506.2 Da). Bottom: representative MS spectrum of cochliodinol (ion formula accuracy: 0.8 ppm)

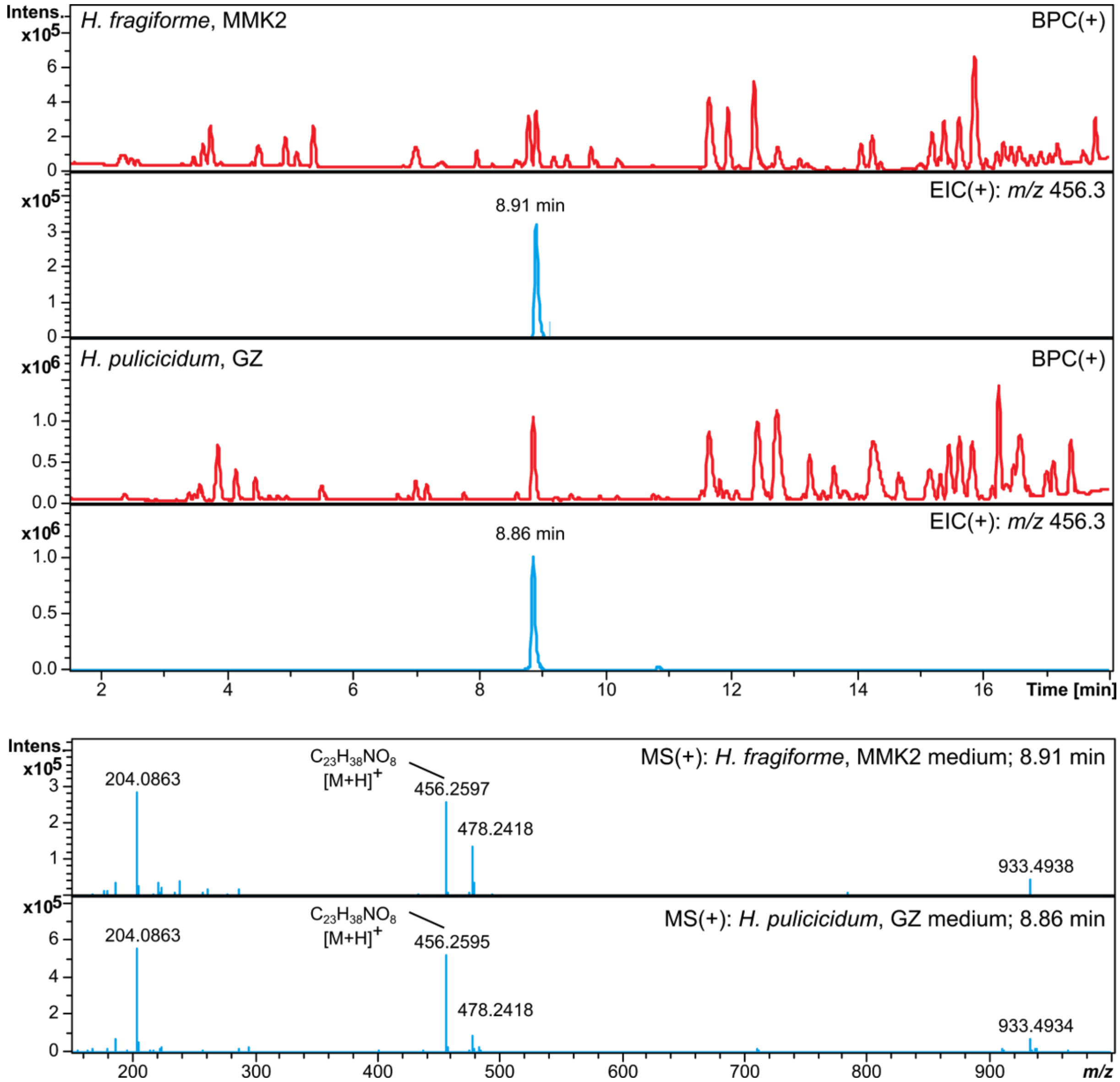

Fig. S13: HPLC-MS chromatograms of culture-derived extracts of *Hypoxylon* spp. (top panel) and MS spectra of brasilane E (bottom). Top: red traces, positive ion mode base peak chromatograms [BPC(+)]; blue traces, extracted ion chromatograms (EICs) of *m/z* 456.3, representing the $[M+H]^+$ ion corresponding to brasilane E (MW 455.3 Da). Bottom: MS spectra of brasilane E identified from *H. fragiforme* (ion formula accuracy: 1.0 ppm) and *H. pulicicidum* (0.6 ppm), respectively.

Hypoxylon fragiforme

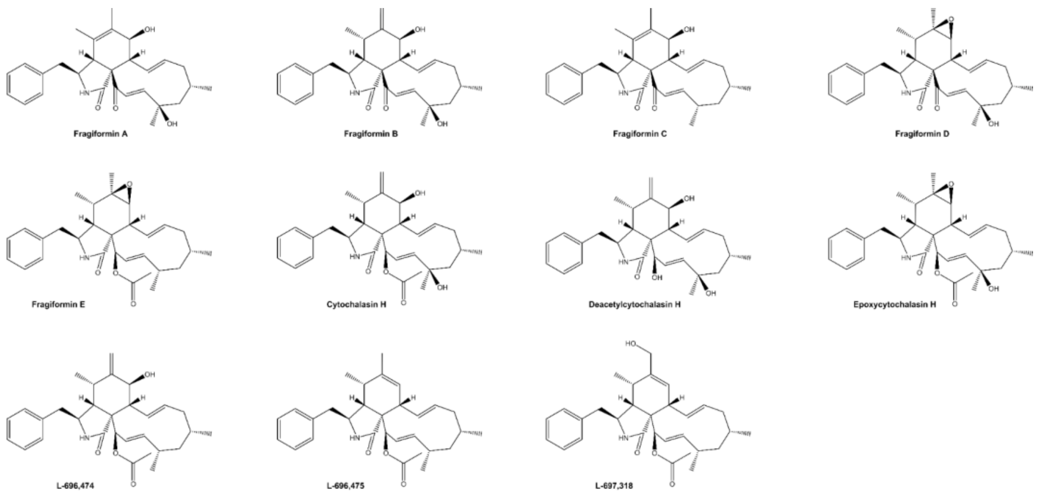

Daldinia concentrica

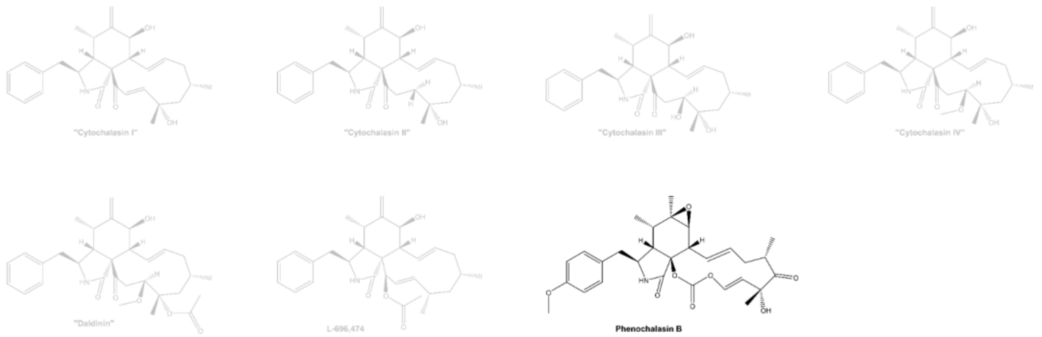

Xylaria hypoxylon

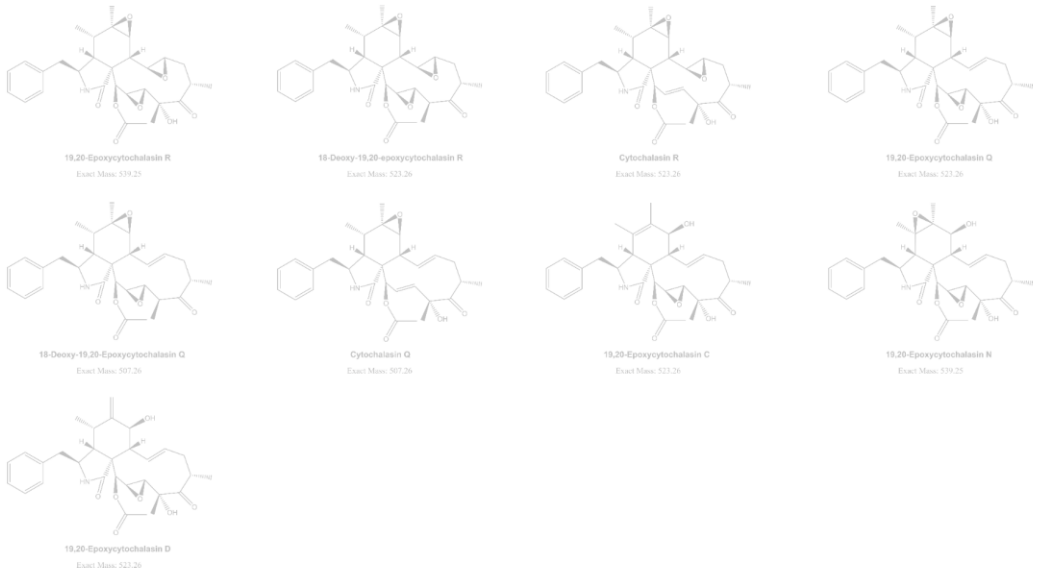

Fig. S14: Cytochalasins reported from *H. fragiforme*, *D. concentrica* and *X. hypoxylon*. Grey structures cannot be explained by the identified biosynthetic gene cluster and are likely derived from misidentified fungi or strain-specific cytochalasin BGCs.

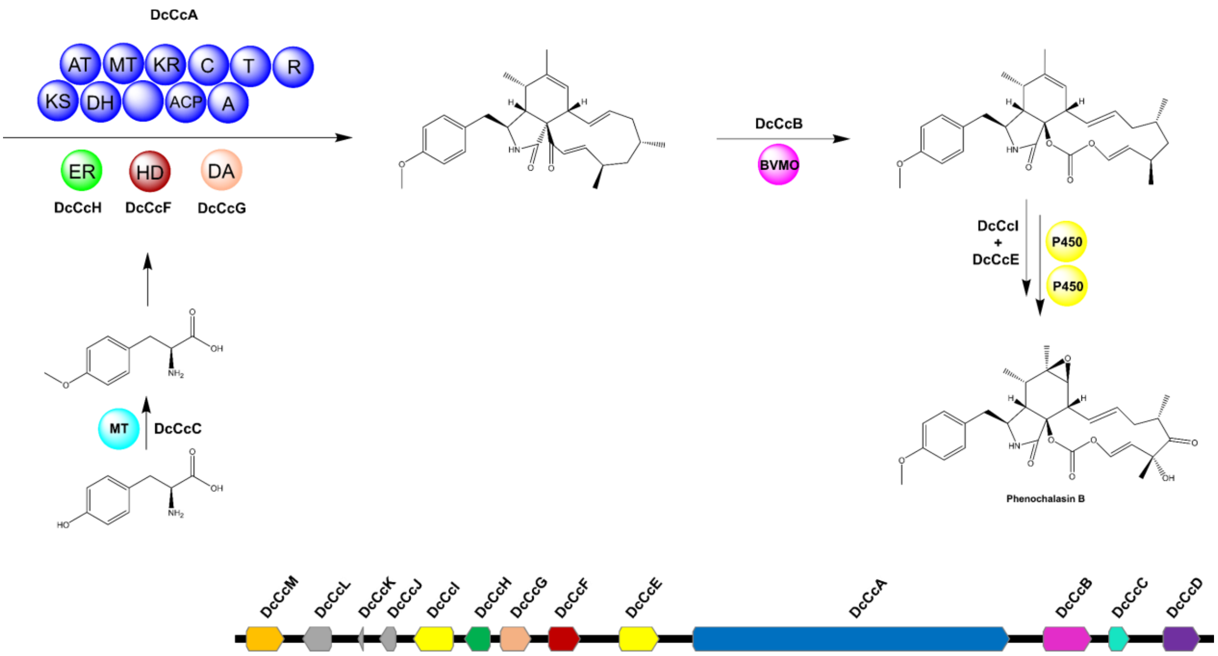

Fig. S15: Proposed biosynthetic pathway for the formation of phenochalasin B in *Daldinia concentrica*. Putative intermediates are shown in grey. The biosynthetic gene cluster is shown at the bottom.

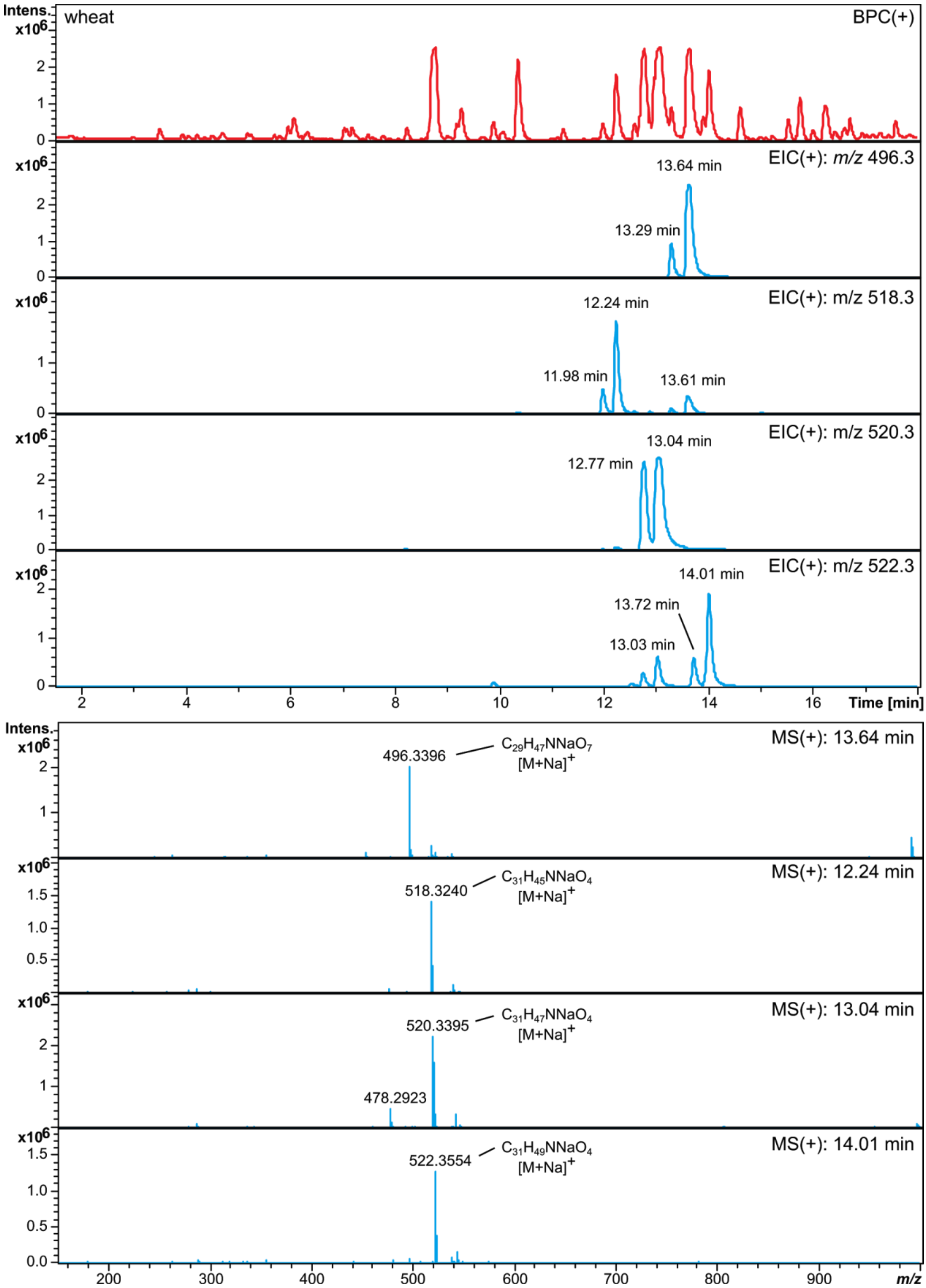

Fig. S16: HPLC-MS chromatograms of culture-derived extracts of *Xylaria hypoxylon* (top panel) and MS spectra of peaks tentatively identified as cytochalasans (bottom). Top: red traces, positive ion mode base peak chromatograms [BPC(+)]; blue traces, extracted ion chromatograms [EIC(+)], representing the $[M+Na]^+$ ions corresponding to putative cytochalasans. Bottom: MS spectra of respective putative cytochalasans (ion formulae accuracy: 0.2, 0.1, 0.0, and 0.0 ppm, respectively).

References

Balibar CJ, Howard-Jones AR, Walsh CT (2007). Terrequinone A biosynthesis through L-tryptophan oxidation, dimerization and bisprenylation. *Nature Chemical Biology* **3**: 584–592.

Becker K, Pfütze S, Kuhnert E, *et al.* (2021). Hybridorubrins A–D: Azaphilone Heterodimers from Stromata of *Hypoxylon fragiforme* and Insights into the Biosynthetic Machinery for Azaphilone Diversification. *Chemistry – A European Journal* **27**: 1438–1450.

Braesel J, Götze S, Shah F, *et al.* (2015). Three redundant synthetases secure redox-active pigment production in the basidiomycete *Paxillus involutus*. *Chemistry and Biology* **22**: 1325–1334.

Chen W, Chen R, Liu Q, *et al.* (2017). Orange, red, yellow: biosynthesis of azaphilone pigments in *Monascus* fungi. *Chemical Science* **8**: 4917–4925.

Chiang YM, Szewczyk E, Davidson AD, *et al.* (2009). A gene cluster containing two fungal polyketide synthases encodes the biosynthetic pathway for a polyketide, asperfuranone, in *Aspergillus nidulans*. *Journal of the American Chemical Society* **131**: 2965–70.

Davison J, Al Fahad A, Cai M, *et al.* (2012). Genetic, molecular, and biochemical basis of fungal tropolone biosynthesis. *Proceedings of the National Academy of Sciences of the United States of America* **109**: 7642–7647.

Derntl C, Guzmán-Chávez F, Mello-de-Sousa TM, *et al.* (2017). *In vivo* study of the sorbicillinoid gene cluster in *Trichoderma reesei*. *Frontiers in microbiology* **8**: 2037.

Forseth RR, Amaike S, Schwenk D, *et al.* (2013). Homologous NRPS-like gene clusters mediate redundant small-molecule biosynthesis in *Aspergillus flavus*. *Angewandte Chemie - International Edition* **52**: 1590–1594.

Geib E, Gressler M, Viediernikova I, *et al.* (2016). A non-canonical melanin biosynthesis pathway protects *Aspergillus terreus* conidia from environmental stress. *Cell Chemical Biology* **23**: 587–597.

Gerke J, Bayram Ö, Feussner K, *et al.* (2012). Breaking the silence: Protein stabilization uncovers silenced biosynthetic gene clusters in the fungus *Aspergillus nidulans*. *Applied and Environmental Microbiology* **78**: 8234–8244.

Guo CJ, Sun WW, Bruno KS, *et al.* (2014). Molecular genetic characterization of terreic acid pathway in *Aspergillus terreus*. *Organic letters* **16**: 5250–3.

Hühner E, Backhaus K, Kraut R, *et al.* (2018). Production of α-keto carboxylic acid dimers in yeast by overexpression of NRPS-like genes from *Aspergillus terreus*. *Applied Microbiology and Biotechnology* **102**: 1663–1672.

Itoh T, Tokunaga K, Matsuda Y, *et al.* (2010). Reconstitution of a fungal meroterpenoid biosynthesis reveals the involvement of a novel family of terpene cyclases. *Nature Chemistry* **2**: 858–864.

Kahlert L, Cox RJ, Skellam E (2020). The same but different: multiple functions of the fungal flavin dependent monooxygenase SorD from *Penicillium chrysogenum*. *Chemical Communications* **56**: 10934–10937.

Lin TS, Chiang YM, Wang CCC (2016). Biosyntheticpathway of the reduced polyketide product citreoviridin in *Aspergillus terreus* var. *aureus* revealed by heterologous expression in *Aspergillus nidulans*. *Organic letters* **18**: 1366–9.

Nofiani R, de Mattos-Shipley K, Lebe KE, *et al.* (2018). Strobilurin biosynthesis in basidiomycete fungi. *Nature Communications* **9**.

O'Leary SE, Hicks KA, Ealick SE, *et al.* (2009). Biochemical characterization of the HpxO enzyme from

Klebsiella pneumoniae , a novel FAD-dependent urate oxidase. *Biochemistry* **48**: 3033–3035.

Rojas-Aedo JF, Gil-Durán C, Del-Cid A, *et al.* (2017). The biosynthetic gene cluster for andrastin A in *Penicillium roqueforti*. *Frontiers in microbiology* **8**: 813.

Schneider P, Bouhired S, Hoffmeister D (2008). Characterization of the atromentin biosynthesis genes and enzymes in the homobasidiomycete *Tapinella panuoides*. *Fungal Genetics and Biology* **45**: 1487–1496.

Schor R, Schotte C, Wibberg D, *et al.* (2018). Three previously unrecognised classes of biosynthetic enzymes revealed during the production of xenovulene A. *Nature Communications* **9**: 1963.

Semeiks J, Borek D, Otwinowski Z, *et al.* (2014). Comparative genome sequencing reveals chemotype-specific gene clusters in the toxigenic black mold *Stachybotrys*. *BMC genomics* **15**: 590.

Tsukada K, Shinki S, Kaneko A, *et al.* (2020). Synthetic biology based construction of biological activity-related library of fungal decalin-containing diterpenoid pyrones. *Nature communications* **11**: 1830.

Wackler B, Lackner G, Chooi YH, *et al.* (2012). Characterization of the *Suillus grevillei* quinone synthetase GreA supports a nonribosomal code for aromatic α-keto acids. *ChemBioChem* **13**: 1798–1804.

Wang B, Li X, Yu D, *et al.* (2018). Deletion of the epigenetic regulator GcnE in *Aspergillus niger* FGSC A1279 activates the production of multiple polyketide metabolites. *Microbiological research* **217**: 101–107.

Winter JM, Sato M, Sugimoto S, *et al.* (2012). Identification and characterization of the chaetoviridin and chaetomugilin gene cluster in *Chaetomium globosum* reveal dual functions of an iterative highly-reducing polyketide synthase. *Journal of the American Chemical Society* **134**: 17900–17903.

Yaegashi J, Praseuth MB, Tyan SW, *et al.* (2013). Molecular genetic characterization of the biosynthesis cluster of a prenylated isoindolinone alkaloid aspernidine A in *Aspergillus nidulans*. *Organic letters* **15**: 2862–5.

Yeh HH, Chiang YM, Entwistle R, *et al.* (2012). Molecular genetic analysis reveals that a nonribosomal peptide synthetase-like (NRPS-like) gene in *Aspergillus nidulans* is responsible for microperfuranone biosynthesis. *Applied Microbiology and Biotechnology* **96**: 739–748.

Zabala AO, Xu W, Chooi YH, *et al.* (2012). Characterization of a silent azaphilone gene cluster from *Aspergillus niger* ATCC 1015 reveals a hydroxylation-mediated pyran-ring formation. *Chemistry and Biology* **19**: 1049–1059.

Zaehle C, Gressler M, Shelest E, *et al.* (2014). Terrein biosynthesis in *Aspergillus terreus* and its impact on phytotoxicity. *Chemistry & Biology* **21**: 719–731.

Zhai Y, Li Y, Zhang J, *et al.* (2019). Identification of the gene cluster for bistropolone-humulene meroterpenoid biosynthesis in *Phoma* sp. *Fungal Genetics and Biology* **129**: 7–15.
